# Physicochemical, antioxidant properties of carotenoids and its optoelectronic and interaction studies with chlorophyll pigments

**DOI:** 10.1038/s41598-021-97747-w

**Published:** 2021-09-15

**Authors:** Ruby Srivastava

**Affiliations:** CSIR-Centre for Cellular and Molecular Biology, Hyderabad, India

**Keywords:** Biochemistry, Computational biology and bioinformatics

## Abstract

The physicochemical and antioxidant properties of seven carotenoids: antheraxanthin, β-carotene, neoxanthin, peridinin, violaxanthin, xanthrophyll and zeaxanthin were studied by theoretical means. Then the Optoelectronic properties and interaction of chlorophyll-carotenoid complexes are analysed by TDDFT and IGMPLOT. Global reactivity descriptors for carotenoids and chlorophyll (Chl*a*, Chl*b*) are calculated via conceptual density functional theory (CDFT). The higher HOMO–LUMO (HL) gap indicated structural stability of carotenoid, chlorophyll and chlorophyll-carotenoid complexes. The chemical hardness for carotenoids and Chlorophyll is found to be lower in the solvent medium than in the gas phase. Results showed that carotenoids can be used as good reactive nucleophile due to lower µ and ω. As proton affinities (PAs) are much lower than the bond dissociation enthalpies (BDEs), it is anticipated that direct antioxidant activity in these carotenoids is mainly due to the sequential proton loss electron transfer (SPLET) mechanism with dominant solvent effects. Also lower PAs of carotenoid suggest that antioxidant activity by the SPLET mechanism should be a result of a balance between proclivities to transfer protons. Reaction rate constant with Transition-State Theory (TST) were estimated for carotenoid-Chlorophyll complexes in gas phase. Time dependent Density Functional Theory (TDDFT) showed that all the chlorophyll (Chl*a*, Chl*b*)–carotenoid complexes show absorption wavelength in the visible region. The lower S_1_–T_1_ adiabatic energy gap indicated ISC transition from S_1_ to T_1_ state.

## Introduction

Chlorophyll and carotenoid pigments can be used as important optical molecular probes to observe different phases of plant performances and its development^1^. Both Chlorophyll and carotenoid are biosynthesized in chloroplast. The synthesized plant carotenoids accumulate exclusively in plastids, and most importantly, chloroplast and chromoplast^2^. Carotene and xanthophylls, both the oxygenated derivatives of carotenes are health promoters and have ability to quench singlet oxygen and scavenge toxic free radicals preventing or reducing damage to living cells. Carotenoids are also used to prolong the shelf life of pharmaceuticals as they have the ability to scavenge free radicals^3–5^. Carotenoids react chemically with the free radicals and the system of conjugated double bonds is directly destroyed^6–8^. Due to their long conjugated chains, carotenoids are highly reactive. The action of free radicals and other reactive oxygen species (ROS) partly causes diseases such as cancers, cerebral thrombosis and infarction^9^. The small amount of adsorbed oxygen in the lungs is used to make harmful ROS, as hydrogen peroxide (H_2_O_2_) and the superoxide radical anion (O2^·−^), which when reacted with transition metals (Fe, Cu) in the human body, produce very reactive ROS [^·^OH] radicals, which causes harm to cells in the human body^9,10^. Seven hundred characterized natural carotenoids are synthesized by plants and microorganisms that confer the yellow, orange and red colors. Twenty carotenoids have been detected in the human blood stream and tissues^11^. Carotenoids are also used as nutritional supplements in food and pharmaceutical industries and in cosmetics due to their bright colors, nutrition and absorption of UV light. The antioxidant and prooxidant effects of carotenoids depends on various factors such as the concentration of carotenoids, molecular structure, action sites, oxygen pressure, interaction with other dietary antioxidants, and the methods used to induce oxidative stress^12^.

The function and properties of chlorophyll and carotenoid reside in their chemical structure. Chlorophylls are cyclic tetrapyrroles carrying a characteristic isocyclic five-membered ring, while carotenoid have C40-tetraterpenoid skeleton which are classified in two groups as carotenes and xanthophylls. Plant chlorins (chl*a* and chl*b*) have absorption bands around the blue and red spectral region in organic solvents. The structure of carotenoid is characterized by a linear chain of conjugated π-electron double bonds, while in oxygenic organisms, carotenoid usually contain ring structures at each end, and most carotenoids contain oxygen atoms, usually as part of hydroxyl or epoxide groups. The absorption maxima is affected by the length of chromophore, the position of the end double bond in the chain or ring and the taking out of conjugation of one double bond in the ring or eliminating it through epoxidation. Carotenoids generally have three-peaked absorption spectrum with well-defined maxima and minima though they show different optical characteristics in various solvents which depend on the polarizability of the solvent^13,14^. The introduction of a carbonyl group in conjugation with the polyene system produces a bathochromic shift and the loss of fine structure^15^, while β-carotene, cryptoxanthin and zeaxanthin all produces identical absorption spectrums as the influence of other substituents group are negligible. The biosynthesis and accumulation of carotenoids in dark-grown etiolated seedling are essential for the assembly of membrane structure and benefits the development of chloroplast when seedlings emerge into the light^16^. For photosynthetic systems, carotenoid is the associated pigment which collects light energy in the spectral region and transfers this energy to chl pigment^17,18^ as chl does not absorb this energy. Also in photoprotection, the role of carotenoid is to quench the triplet state of chl before it reacts with oxygen to form singlet oxygen species (ROS)^19,20^. Carotenoid regulates energy transfer in the light-harvesting antenna through xanthophyll cycle, to avoid over-excitation of the photosynthetic system by safely dissipating excess energy^21,22^. So understanding the relationship between structure and photophysical properties of these pigments can provide insights into a better study of how photosynthesis works at the molecular level in chloroplast.

In this work, the physicochemical and antioxidant properties of seven carotenoids: antheraxanthin (a), β-carotene (b), neoxanthin (n), peridinin (p), violaxanthin (v), xanthrophyll (x) and zeaxanthin (z) are studied by DFT method. Then the optoelectronic properties and interaction of these seven carotenoids were studied with chlorophyll Chl*a*, Chl*b*. These fourteen complexes are Chl*a-*antheraxanthin (**Chlaa**), Chl*a-*β-carotene (**Chlab**), Chl*a-* neoxanthin (**Chlan**), Chl*a-*peridinin (**Chlap**), Chl*a-*violaxanthin (**Chlav**), Chl*a-*xanthrophyll (**Chlax**), Chl*a-*zeaxanthin (**Chlaz**) and Chl*b-*antheraxanthin (**Chlba**), Chl*b-*β-carotene (**Chlbb**), Chl*b-*neoxanthin (**Chlbn**), Chl*b-*peridinin (**Chlbp**), Chl*b-*violaxanthin (**Chlbv**), Chl*b-*xanthrophyll (**Chlbx**), Chl*b-*zeaxanthin (**Chlbz**) respectively. C-DFT (Conceptual Density functional Theory) has been used to calculate the global chemical descriptors of the carotenoids. The absorption properties for carotenoid, chlorophyll and chlorophyll-carotenoid have been studied by TDDFT method in water.

## Theoretical methods

**Figure 1:**
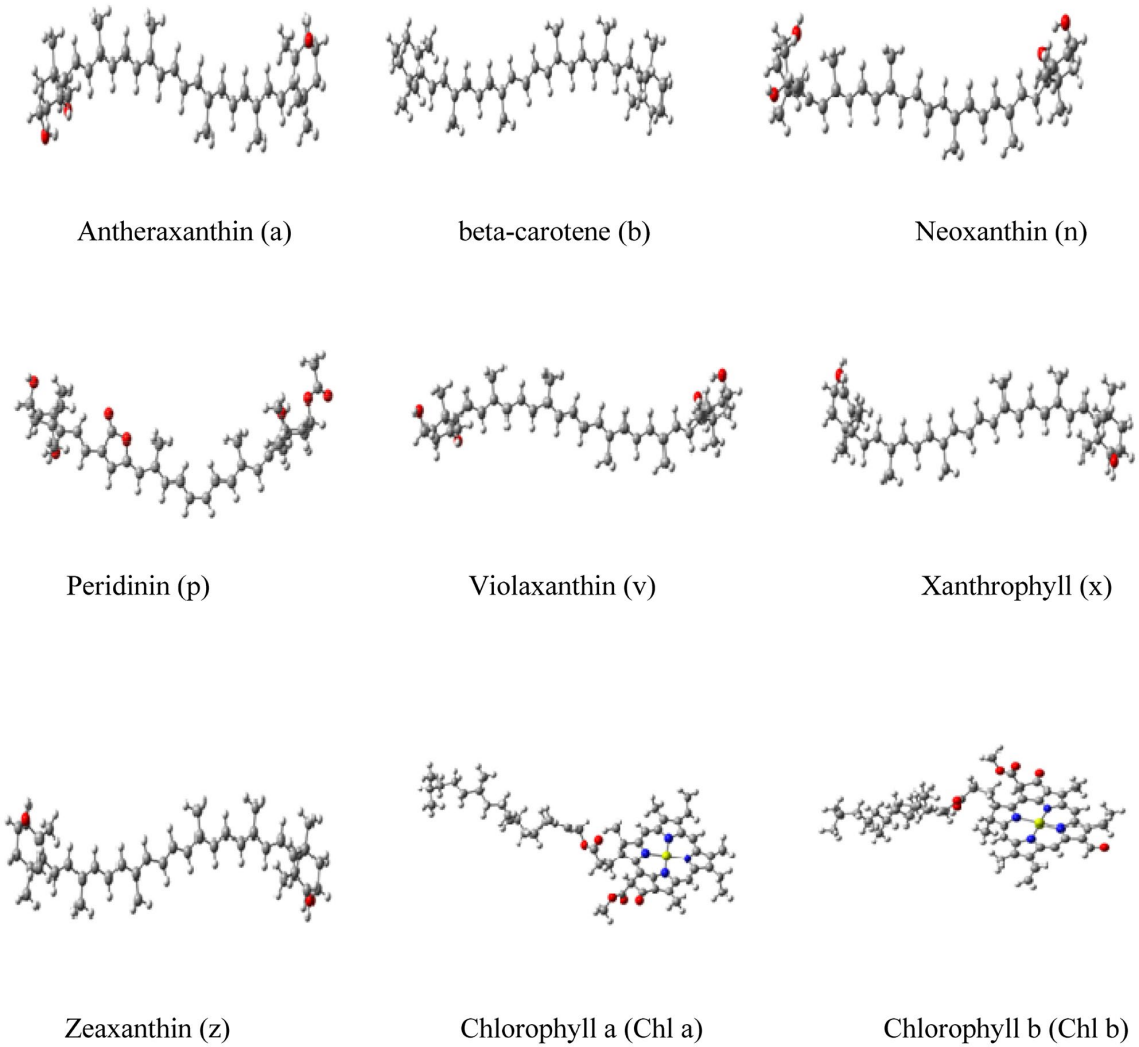
Optimized structures of carotenoids (wB97xD/6-31G**) and Chl*a* and Chl*b* (wB97xD/Lanl2dz:6-31G**) method by DFT method.

First, a conformational analysis was performed by random rotations of the freely rotating bonds in the carotenoid complexes and chlorophylls in the range of 0^◦^ to 360^◦^, generating 50 random structures for each system in this manner. Then the carotenoids are optimized by three different methods CAM-B3LYP^23^/6-31G**, wB97xD^24^/6-31G** and M062x^25^/6-31G** by G16 software programme^26^ in gas phase. As Magnesium is present in Chlorophyll, Chla and Chlb are optimized with CAM-B3LYP/Lanl2dz^27^:6-31G**, wB97xD/Lanl2dz:6-31G** and M062x/Lanl2dz:6-31G** methods with G16 software programme in gas phase. Finally the lowest minima structure by wB97xD/Lanl2dz:6-31G** method was selected to study the physicochemical studies of carotenoid and chlorophyll in gas and solvent (water) medium by integral equation formalism-polarized continuum model (IEF-PCM)^28^ according to the SMD solvation model. The pictorial visualization of Electrostatic potential (ESP) of carotenoids by Gaussview^29^ is given in Supplementary Fig. 1. The optimized structures of carotenoid and chlorophyll are given in Fig. 1.

**Figure 2:**
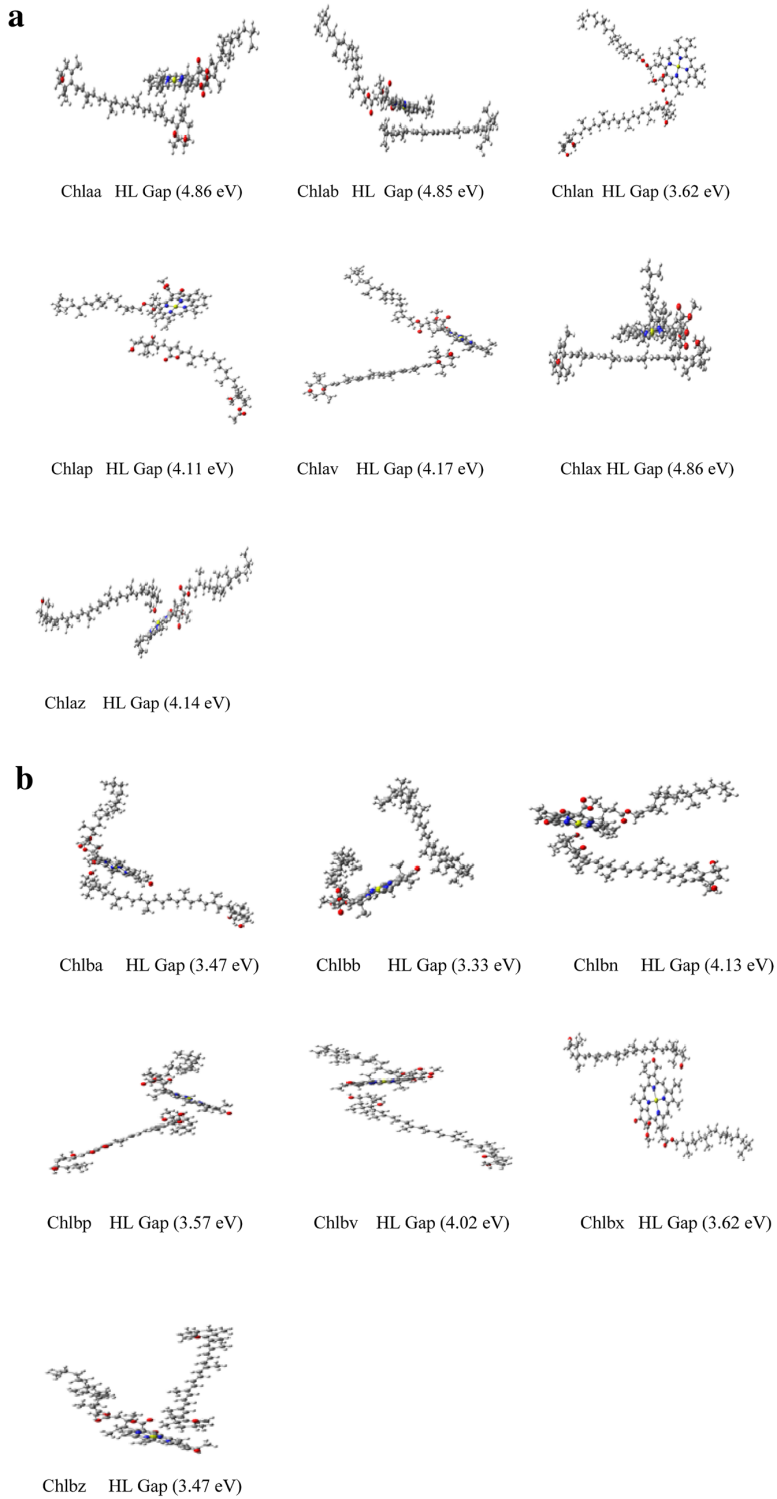
(**a**) Optimized Structures of Chylla-carotenoid complexes with wB97xD/Lanl2dz:6-31G** basis sets in solvent (water) by DFT method. (**b**) Optimized Structures of Chyllb-carotenoid complexes with wB97xD/Lanl2dz:6-31G** basis sets in solvent (water) by DFT method.

The energy values by three different basis sets are given in Supplementary Table 1. Vibrational frequency analysis has been carried out for all complexes and no negative frequency is found. Now with these lowest minima structures of carotenoid and chlorophyll, various sites have been created and all structures are optimized with wB97xD/Lanl2dz:6-31G** method in gas and water. Again the lowest minima structures have been selected for estimation of reaction rate (gas phase) and TDDFT (water phase) calculations. Vibrational frequency analysis has been carried out to check the stability of complexes and no negative frequency has been found. See Fig. 2a and b for Chla-carotenoid and chlb-carotenoid complexes respectively. These optimized structures and IGMPLOT figures are visualized by GaussView programme. The same optimized geometries have been used to generate the intermolecular and intramolecular interactions with IGMPLOT^30^.

Proton transfer reactions are of great importance in biomolecular processes of living organisms. In a gas phase environment, proton affinity (PA), is used which is defined as the negative of the enthalpy change at standard condition [i.e. temperature (298 K) and pressure]. Computational ab initio approaches can provide reliable values for proton affinities, which is important since it is difficult to determine experimentally. Chemical reactivity parameters have been used to predict the quantitative reactivity of the molecular system. The formulas used to calculate the proton affinities and global energy descriptors are given in Supplementary Information.

Antioxidants play very important role in the inhibition of the oxidative damage of the biomolecules. So quantitative analysis of the antioxidant activity of carotenoids has been obtained by the structure determination, energetics and global reactivity descriptors of carotenoid in gaseous and solvent (water) medium by DFT.

Transitivity code^31^ has been used to calculate the reaction rate constant with Transition-State Theory (TST) for reactions in gaseous phase. The graph Log K versus 1000/K were plotted for the fourteen carotenoid-chlorophyll complexes for the default temperature range 273.15 K to 4000.00 K. In this window, it is possible to estimate reaction rate constant with TST and several one-dimensional tunneling corrections, thermodynamic (∆*E*, ∆*H* and ∆*G*) and kinetic (*Eo*, d, ν‡, and α from ST model) properties of the reaction. β = 1⁄*kBT*, where *kB* is the Boltzmann’s constant. The transition state calculations for carotenoid, chlorophyll and carotenoid-chlorophyll complexes have been carried out by G09^29^ programme.

IGM (independent gradient model) represents a non-interacting system and the true ED gradient ($\left| {\nabla_{\rho } } \right|$). It can be seen as a measure of electron sharing brought by ED contragradience. This model provides an automatic workflow that extracts the signature of interactions between selected groups of atoms. Also noncovalent interaction (NCI) approach provides a visual understanding of the interactions present in chemical systems with both intermolecular and intramolecular interactions and to see the bond-by-bond picture that can be obtained from a wave function. In this way the specific interactions along reaction paths can easily monitor and the signature of inter and intra molecular interactions can be extracted. IGMPLOT is used to assess the role of non-covalent intramolecular interactions (intramolecular π–π stacking or hydrogen-bonding between two part of a single molecule along a reaction path).

TDDFT calculations for the complexes have been carried out on the ground state stable geometies with wB97xD/Lanl2dz:6-31G** in solvent (water). The absorption wavelength, oscillatory strength and transitions for carotenoids, chlorophyll and chlorophyll-carotenoid interactions are given in Table 3 and Table 4 respectively. Transitions (%) are calculated by GaussSum^32^ software.

## Results

The reactivity of seven carotenoids is studied by DFT-based global reactivity descriptors using Chemical reactivity theory (CRT). C-DFT^33–37^ used global reactivity parameters to predict structural and electronic properties of reactants and products which occur due to the chemical transformations during the reactions. It also gives a better microscopic insight to the whole interaction insight processes. To characterize the antioxidant property of carotenoids, it is important to analyse the global reactivity descriptors in detail. Chemical hardness measure the resistance to charge transfer, while the electronegativity measure the tendency to attract electrons in a chemical bond. The maximum electron flow between a donor and an acceptor is governed by the decomposition of binding energy between the atoms and it is determined by the factor electrophilicity index. Carotenoids act as electron donor rather than electron acceptor in the studied environments which is also an indication of their antioxidant activity. The calculated parameters IP, EA, global electronegativity (χ), global hardness (η), global electrophilicity index (ω) and HL gap for all complexes are given in Table 1. An study conducted on Carotenoids of Marine origin displayed higher electrophilicity scale for astaxanthin and lower for zeaxanthin using different functional and solvents^38^.

**Table 1:** Calculated IP, EA, Global reactivity descriptors—electronegativity (χ), global hardness (ƞ), global softness (S) and electrophilicity index (ω) in (eV) for studied carotenoid and Chlorophyll complexes with wB97xD/6-31G** and wB97xD/Lanl2dz6-31G** method respectively by DFT.

Complexes	IP	EA	Electronegativity (χ)	Hardness (ƞ)	Softness (S)	Electrophilicity Index ( ω)
Antheraxanthin (gas)	5.88	0.918	3.40	2.481	0.2015	2.33
(Water)	4.77	2.182	3.48	1.290	0.3864	4.67
β-carotene (gas)	5.79	0.862	3.33	2.464	0.2029	2.24
(Water)	4.73	2.164	3.45	1.280	0.3897	4.63
Neoxanthin (gas)	5.84	0.849	3.35	2.496	0.2004	2.24
(Water)	4.76	2.149	3.45	1.310	0.3830	4.57
Peridinin (gas)	6.16	1.205	3.68	2.478	0.2018	2.74
(Water)	4.99	2.670	3.83	1.160	0.4310	6.32
Violaxanthin (gas)	5.92	0.910	3.41	2.505	0.1996	2.33
(Water)	4.53	2.184	3.36	1.170	0.4263	4.80
Xanthrophyll (gas)	5.83	0.863	3.35	2.484	0.2013	2.25
(Water)	4.75	2.160	3.46	1.300	0.3861	4.61
Zeaxanthin (gas)	5.78	0.854	3.32	2.463	0.2030	2.23
(Water)	4.74	2.170	3.46	1.290	0.3891	4.64
*OH (gas)	16.32	1.77	9.05	7.279	0.069	5.65
(Water)	12.79	5.21	9.01	3.792	0.132	10.71
*OOH (gas)	12.63	0.55	6.59	6.041	0.083	3.602
(Water)	9.489	3.716	6.60	2.887	0.173	7.543
Chlorophyll (Chl) *a *(gas)	5.96	1.395	3.68	2.283	0.2191	2.96
Chlorophyll (Chl) *b *(gas)	7.26	0.665	3.96	3.298	0.1516	2.38

*OH and *OOH parameters are taken from Ref.^41^.

Both carotenoids and Chlorophyll have higher HL gap which indicated structural stability. Chemical hardness of carotenoids is higher in gas state. It was also observed that the HL gap for carotenoids and chlorophyll in solvent is more than the HL gap of these complexes in gaseous state. Our Global chemical reactivity parameters (IP and EA), energy levels and energy gap (HOMO‐ LUMO) results are consistent with the previously calculated results for β‐Carotene, Neoxanthin, Violaxanthin and Zeaxanthin in the gaseous phase, and methanol (solvent) using B3LYP/6‐31+G(d,p) method. The results show narrow HL energy gap which benefits energy transfer process for the carotenoid. The experimental value of the band gap is in the range of (1.87–1.92) eV for Chla and (1.90–1.92) eV for Chlb^39–41^. The HL gap for Chlaz is 1.883 in gas phase^42^, while the HL gap for Chlaz is 4.14 eV in aqueous medium. (See Fig. 2a). For Chla and Chlb, the ionization potentials are determined to be 4.19 eV and 4.45 eV in the PBE/DPZ level; however, it is obtained 4.96 and 5.22 by using the Octopus code, as well as 4.79 and 5.17 by applying the B3LYP/6-31G* hybrid functionals, The dipole moment of Chla (4.79 D) is larger than that of Chlb (1.36 D)^43^. Carotenoids are well-known antioxidants and they have the ability to quench singlet oxygen and scavenge toxic free radicals preventing or reducing damage to living cells. In our studies, the complexes have lower µ and ω which predicted that these carotenoids are good reactive nucleophile, also indicating their antioxidant behaviour. So we calculated proton affinities (PAs) and bond dissociation enthalpies (BDEs) for the studied carotenoids. BDE (numerical parameter) is related to Hydrogen atom transfer (HAT) mechanism and characterizes the stability of the corresponding hydroxyl group. The lower BDE value indicates that the stability of the corresponding O–H bond is lower and the corresponding O–H bond can be easily broken^44–49^. Hence, higher is the antioxidant capacity of the compound. In our studied complexes, proton affinities (PAs) are much lower than the bond dissociation enthalpies (BDEs), which indicate that direct antioxidant activity in these complexes is mainly due to the sequential proton loss electron transfer (SPLET) mechanism^50^. Lower PA of all the complexes indicated that antioxidant activity by the SPLET mechanism is a result of a balance between proclivity to transfer protons and the reaction kinetics of the conjugated base in the sequential electron transfer mechanism^46,47^. See Table 2. The electron transfer between the antioxidant and the radical can be determined from the IP and EA. A lower IP means a higher probability of losing an electron. It is clear from the trends observed in IP values that all these carotenoid molecules are capable of electronic charge transfer to the neutral ROS. This trend is compared to another theoretical studies^51^ carried out for neutral ROS, i.e. ^·^OH and ^·^OOH, the average decrease in the vertical IP in the aqueous medium is 3.334 and 3.330, respectively. The EA also has crucial influence on the electron transfer between the antioxidant and the radical: a higher EA means a higher probability of gaining an electron. EAs obtained for the studied complexes are found to be lower than those for the neutral ROS ^·^OH and ^·^OOH in water medium. The IPs of our studied complexes are lower than those of the neutral ROS and the higher EAs of the neutral ROS compared to those of these molecules support the antioxidant behavior of the carotenoids. We also find that the presence of solvent significantly influences the EA. The η values of the ROS ^·^OH and ^·^OOH are 3.792 and 2.887 eV in the aqueous medium. Hence, the presence of the solvent increases the reactivities of these carotenoids molecules as well as the ROS. Consequently, the higher electrophilicity index predicts greater propensity of the ROS to attract electrons from a generic donor molecule. In our studied complexes the electrophilicity index is lower than the electrophilicity index of ROS ^·^OH and ^·^OOH, which indicates the high propensity of the ROS to attract electrons from these carotenoid molecules. The calculated redox potential is for the carotenoids are higher than the experimental values. The electron donating ability of carotenoid is related with the HOMO energies also. The molecules with higher HOMO orbital energy have stronger electron donating abilities, which is strongly correlated to the IP values also. Thus, the thermodynamically preferred reaction pathway involved in the free radical scavenging process can be determined by the BDEs and PAs. By comparison, it is found that in the gas phase, the calculated PAs of carotenoids are significantly lower than the BDEs, and hence thermodynamically, SPLETT represents the most favorable process in the gas phase.

**Table 2:** Calculated HOMO–LUMO Gap (eV) (gas, water), Proton affinity (PA), bond dissociation energies (BDE) and redox potential (meV) for studied carotenoid complexes and chlorophyll with wB97xD/6-31G**, wB97xD/Lanl2dz:6-31G** method respectively.

Complexes	HL gap (gas)	HL gap (water)	PA	BDE	Redox potential
Antheraxanthin	4.68	4.68	1.275	1.555	591.04
β-carotene	4.65	4.64	1.278	1.529	561.87 537.2 (exp)
Neoxanthin	4.72	4.72	1.276	1.565	560.82
Peridinin	4.61	4.55	1.264	1.428	574.32
Violaxanthin	4.72	4.72	1.272	1.563	577.21
Xanthrophyll	4.69	4.68	1.277	1.546	570.44
Zeaxanthin	4.65	4.64	1.279	1.548	699.88 691.5 (exp)
Chlorophyll (Chl) *a*	4.17	4.18	1.272	2.169	–
Chlorophyll (Chl) *b*	4.39	4.50	1.105	1.962	–

**Figure 3:**
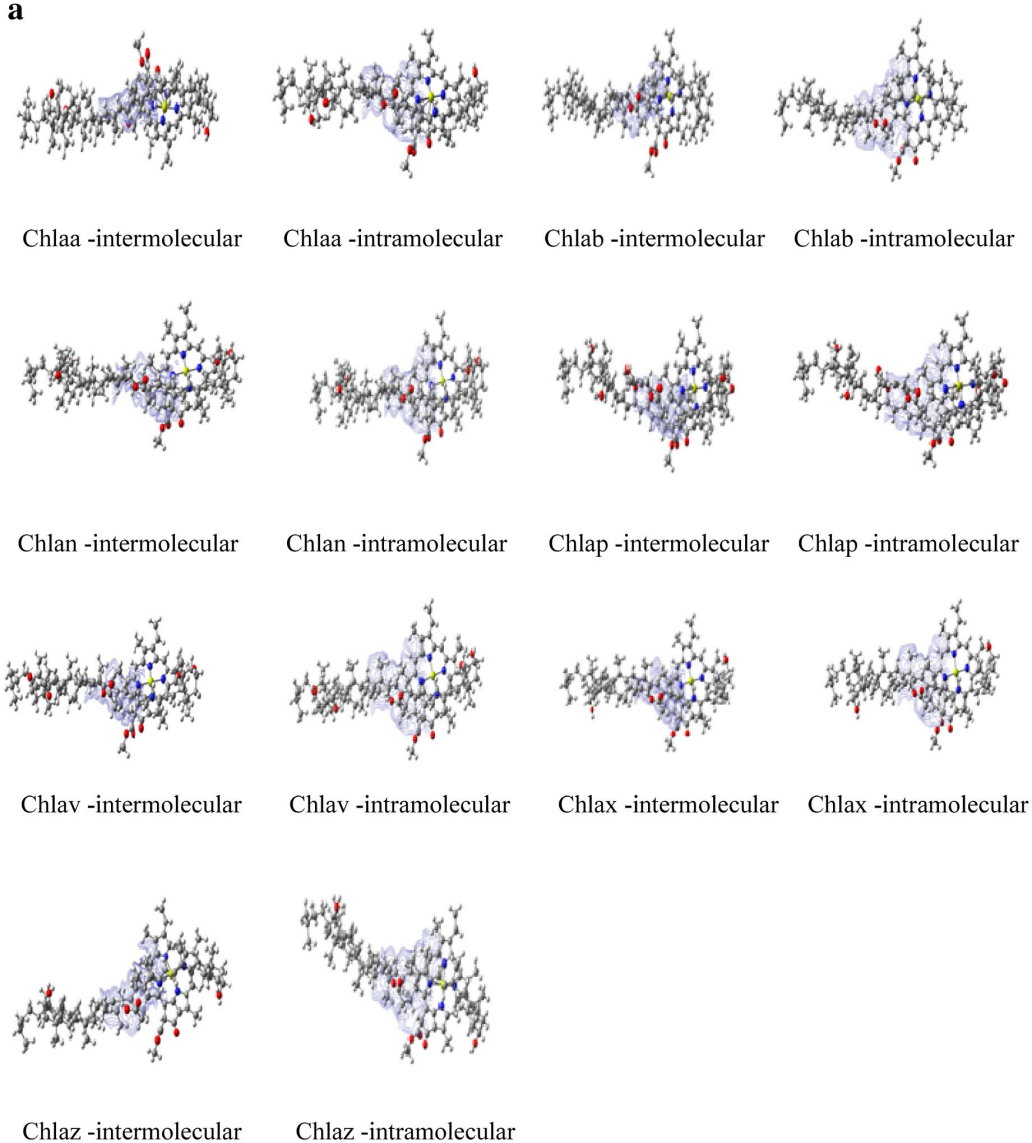

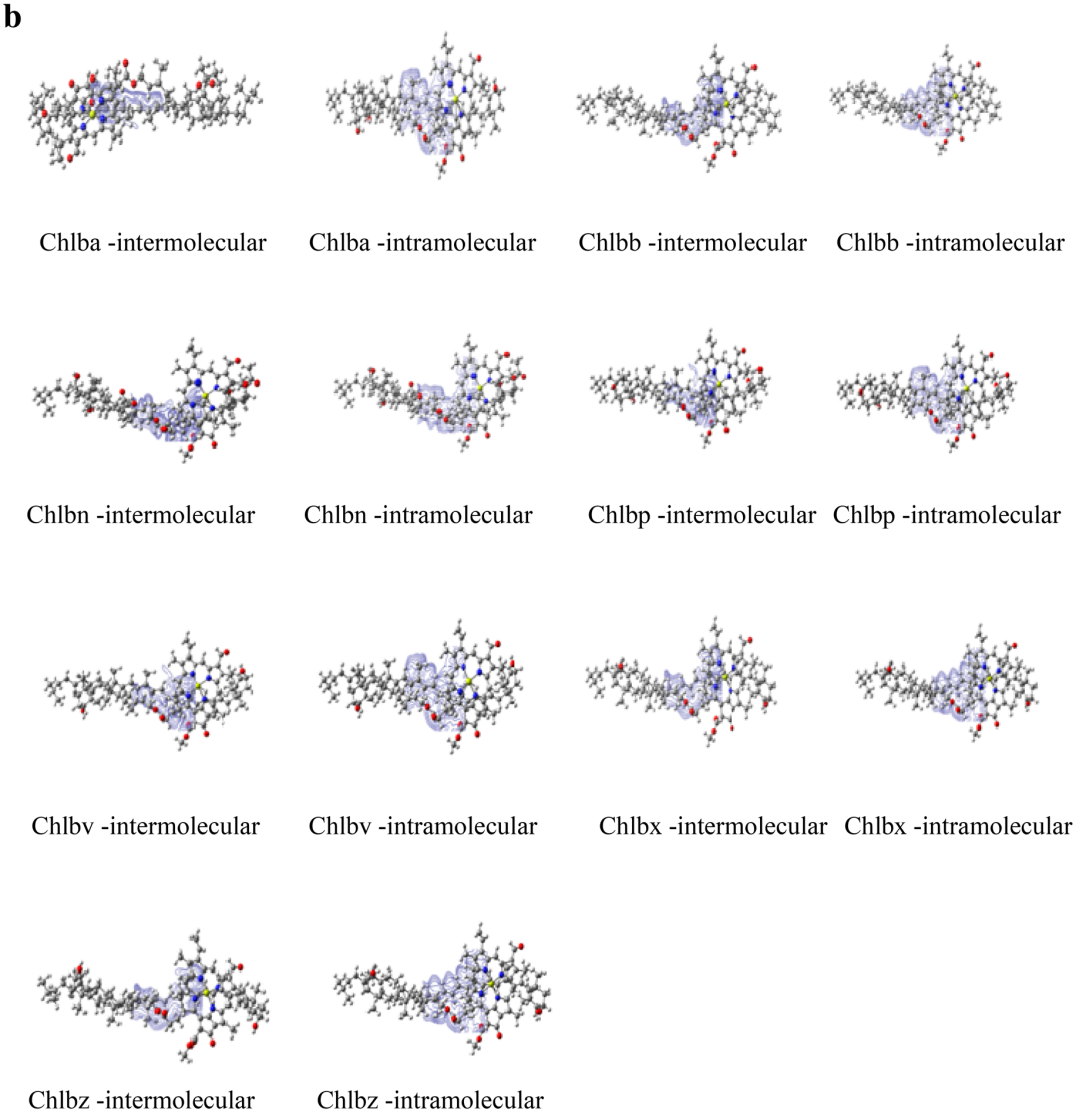
(**a**) Intermolecular and Intramolecular interactions of Chl*a*-carotenoid complexes by IGMPLOT. The visualized images have been taken from Gaussview programme. (**b**) Intermolecular and Intramolecular interactions of Chlb-carotenoid complexes by IGMPLOT. The visualized images have been taken from Gaussview programme.

Strong intermolecular and intramolecular interaction has been observed among these complexes, with blue color isosurface plots between carotenoids and chlorophylls by IGMPLOT. The covalent chemical bond is intimately linked to the electron sharing between atoms. See Fig. 3a and b.

**Figure 4:**
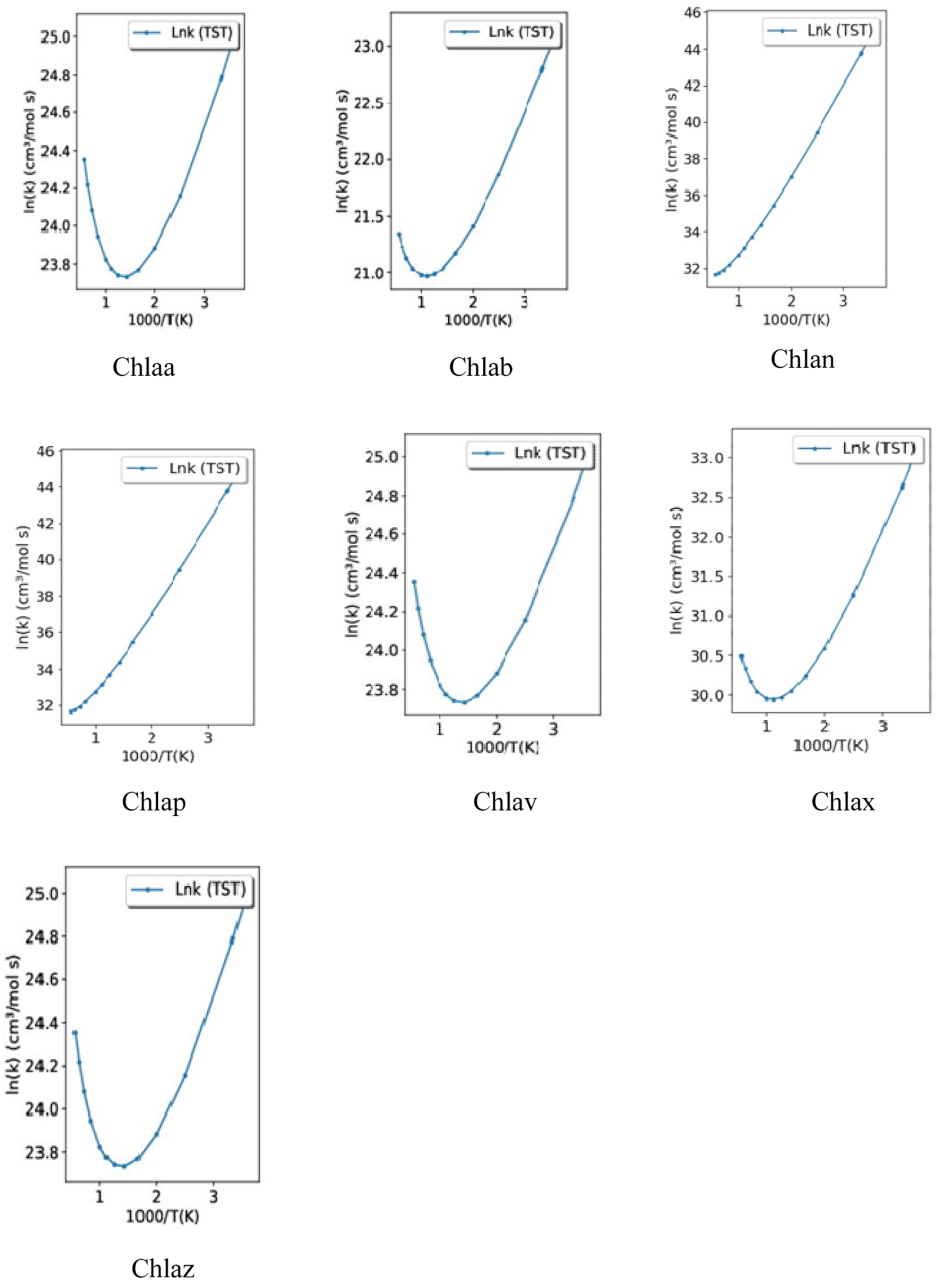

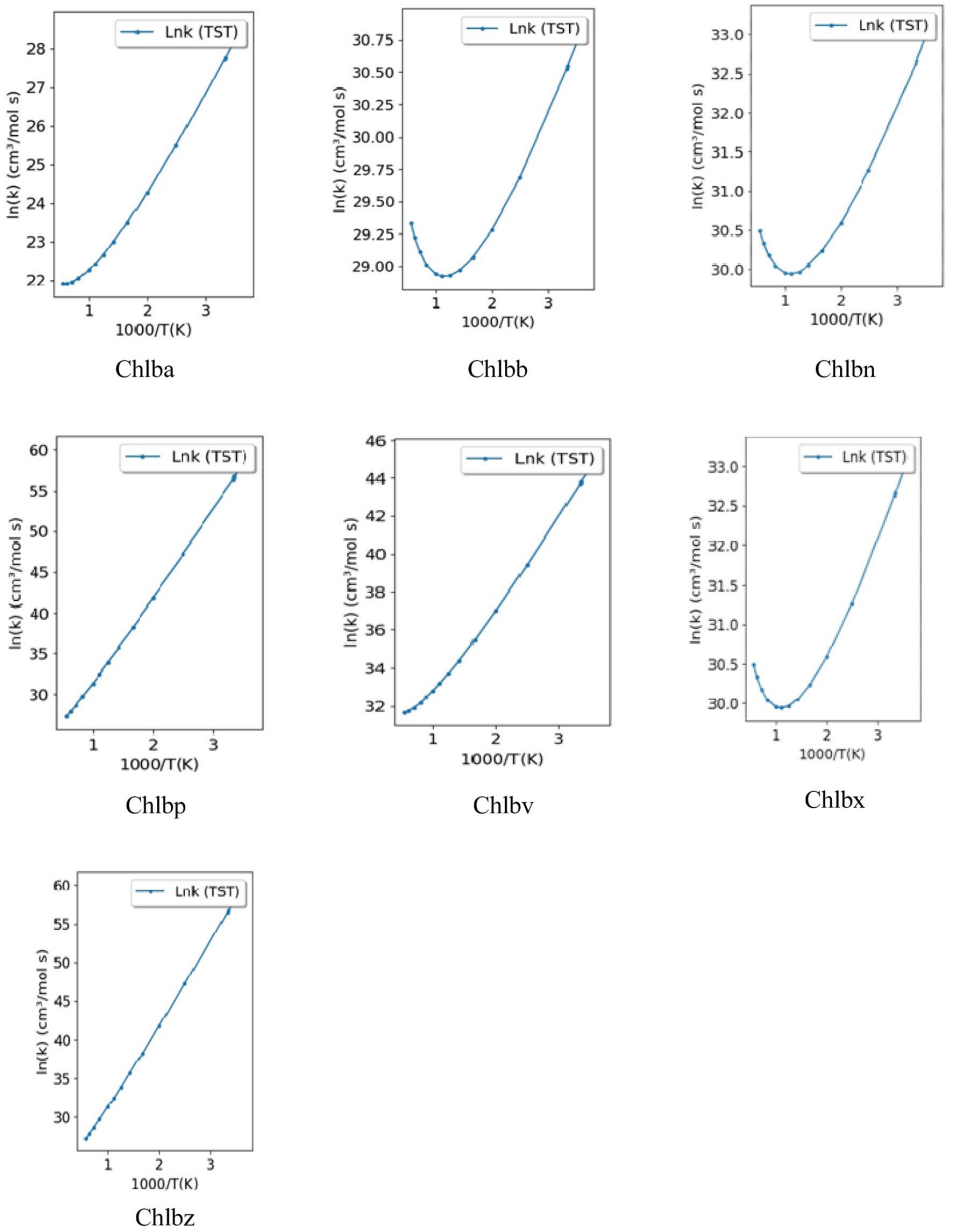
(**a**) Reaction rate constant graph for Chylla-carotenoid complexes (ln(k)) versus 1000/T(K). (**b**) Reaction rate constant graph for Chyllb-carotenoid complexes (ln(k)) versus 1000/T(K).

Transitivity code has been applied to estimate the reaction rate constant with Transition-State Theory (TST) for reactions in gaseous phase. Good correlation has been observed for all fourteen complexes in gas phase. See Fig. 4a and b. The biological activities and the spectral properties are influenced by the variation in the structures of the complexes. The biological activity in plants includes the energy transfer, photoprotection and quenching of damaging singlet oxygen and bioactivities in humans and other organisms^51^. A wide variety of computational investigations have been used for computing excitation energies and absorption profiles, ranging from time-dependent density functional theory (TD-DFT)^52–56^ and DFT/multireference configuration interaction (MRCI) to various wave function methods such as SAC-CI^57^, CC2^58^, and ADC(2)^59^. The calculated position of the first peak is about 1.85 (1.91) eV for Chl a (Chl b)^39^, which agrees well with existing experimental data^40^. In another study the absorption energies of the Qy bands of Chla and chlb in acetone, diethyl ether, or ethanol were in the range (500–700) nm with CAM-B3LYP/e6–31G* basis sets^60^, while experimental results show (672 nm) and (652 nm)﻿ for Chla and Chlb in ethanol^61^. DLPNO−STEOM–CCSD calculations predict two excited states in the Q region, at 1.75 and 2.24 eV for chla in gas phase^62^. while the absorption peaks of chla were observed in (600–700) nm range^63^. Our TDDFT calculations show that the absorption wavelengths of the carotenoids are in visible region (432–452) nm, while chl*a* and chl*b* has (356, 380) nm wavelength (water) respectively. See Table 3. The oscillatory strength of carotenoids are > 2, which is justified by the other theoretical results^64^. The wavelengths for β‐Carotene, Neoxanthin, Violaxanthin and Zeaxanthin in the gaseous phase and methanol (solvent) using TDDFT with the CAM‐B3LYP/6‐31+G(d,p) are (293–303) nm^42,65^, which is slightly lower than our TDDFT results (430–452) nm measured in aqueous solvent. In one of the studies, the calculated TDDFT Excited States Energies (eV) were (2.20–3.65) eV using SVWN, B3LYP, and CAM-B3LYP Functionals for peridinin using Quantum Monte Carlo and Many Body Green’s Function Theory^66^.

**Table 3:** Absorption wavelength (nm) (Singlet states and Triplet states), Oscillatory Strength (*f*) and transitions for studied carotenoids (wB97xD/6-31G**) and Chlorophyll (wB97xD/Lanl2dz:6-31G**) by TDDFT in solvent (water) medium.

Complexes	Singlet states			Triplet states		
	Wavelength (nm)	*f*	Transitions	Wavelength (nm)	*f*	Transitions
Antheraxanthin	436.35	4.1365	H → L (89%)	605.24	1.8395	H → L (69%)
β-carotene	440.51	4.1761	H → L (89%)	604.95	2.7927	H → L + 1 (38%)
Neoxanthin	432.08	4.2227	H → L + 1 (90%)	597.40	2.3260	H → L + 2 (34%)
Peridinin	452.09	2.8365	H → L (90%)	642.31	2.2258	H → L (33%)
Violaxanthin	431.63	4.1153	H → L (90%)	588.16	1.6288	H → L (23%)
Xanthrophyll	435.99	4.0844	H → L + 1 (89%)	596.28	2.4379	H → L + 2 (35%)
Zeaxanthin	440.49	4.1694	H → L (89%)	605.72	3.5519	H-1 → L (48%)
Chlorophyll (Chl) *a*	356.57	1.1354	H → L + 1 (73%)	750.65	0.0003	H-2 → L (68%)
Chlorophyll (Chl) *b*	380.98	1.4282	H → L + 1 (68%)	675.85	0.0098	H-3 → L (68%)

The Chla-carotenoid and Chlb-carotenoid complexes have wavelength (432–476) nm in visible region. Higher (89–90)% HOMO → LUMO transitions was observed for carotenoids, while chlorophyll has (68, 73)% transitions for HOMO → LUMO + 1. The % transitions are higher for carotenoid-chlorophyll complexes also. See Table 4. chlaz diade has absorption wavelength (340 nm) using CAM-B3LYP/6‐31+G(d,p) method^42^, while chlap diade has two absorption wavelength peaks in (440–480) nm range in aqueous state with MM/QM method^63^.

**Table 4:** Absorption wavelength (nm) (Singlet States), Oscillatory strength and transitions for Chla-carotenoid and Chlb-carotenoid complexes with wB97xD/Lanl2dz:6-31G** method by TDDFT in solvent (water) medium.

Complexes	λ_abs_	*f*	Transitions	Complexes	λ_abs_	*f*	Transitions
Chlaa	440.47	4.3868	H → L + 1 (89%)	Chlba	440.82	3.8688	H → L + 2 (89%)
Chlab	444.36	3.4976	H → L + 1 (89%)	Chlbb	447.34	4.2932	H → L + 2 (87%)
Chlan	433.25	4.4038	H → L + 1 (89%)	Chlbn	554.23	4.7344	H → L + 2 (91%)
Chlap	475.92	2.5918	H → L + 1 (89%)	Chlbp	476.46	2.7656	H → L + 1 (90%)
Chlav	432.85	4.2830	H → L + 1 (89%)	Chlbv	432.91	4.1286	H → L + 2 (91%)
Chlax	444.21	2.8298	H → L (89%)	Chlbx	442.24	4.0839	H → L + 2 (88%)
Chlaz	445.63	4.3302	H → L + 1 (89%)	Chlbz	444.76	4.4578	H → L + 2 (89%)

The first excited triplet state transitions were calculated with wB97xD/6-31G** basis sets for Chl(a,b) and carotenoids and wB97xD/Lanl2dz:6-31G** basis sets were used for the Chlorophyll-carotenoid interactions. The triplet wavelength for chlorophyll and carotenoid lies in (588–750) nm range (See Table 3) whiles the first triplet wavelength for chl-car complexes lies in (432–555) nm range. See Table 5. The results are consistent with the previous experimental results for ppLHCSR1-Vio (488 nm) and ppLHCSR1-Zea (491 nm) at pH 7. When pH is lowered to 5, the carotenoid bands slightly red-shifts to 489 nm (ppLHCSR1-Vio) and 492 nm (ppLHCSR1-Zea)^67^. In another studies on Chl (a, b), the triplet energy is 0.8 eV lower than that of the S1 state^68^. The S_1_–T_1_ adiabatic energy gap is lower for the studied complexes which is an implication on the ISC transition from S_1_ to T_1_ state. According to the energy gap law^69,70^, for electronic states with similar geometries, the smaller the energy gap, the larger is the ISC rate. So there will have a faster ISC rate for triplet formation in chl-car complexes. Certainly, carotenoids play major role in the photosynthetic apparatus for quenching of the Chl triplet state (^3^Chl) through the triplet–triplet energy transfer (TTET) mechanism^71–73^. The direct population of the carotenoid triplet state (^3^Car) by intersystem crossing (ISC) is a low probability event, due to a very short lifetime of the excited state of these molecules, that is dominated by internal conversion. Energy transfer from the ^3^Chl, which is populated with a yield of ~ 0.6 in the absence of other quenching mechanisms^74,75^, is efficient in photosynthetic systems^70,76^ because of the short average inter-pigment distances and because the ^3^Car lays at an energy level which is below that of ^3^Chl. Also it is well known that ^3^Chl is an efficient sensitiser of singlet oxygen (^1^O_2_), which is an highly reactive species and plays major role in photo-oxidative stress^71,72,77^.

**Table 5:** Absorption wavelength (nm) (Triplet states), Oscillatory strength and transitions for Chla-carotenoid and Chlb-carotenoid complexes with wB97xD/Lanl2dz:6-31G** method by TDDFT in solvent (water) medium.

Complexes	λ_abs_	*f*	Transitions	Complexes	λ_abs_	*f*	Transitions
Chlaa	793.87	0.00030	H-1 → L (42%)	Chlba	715.88	0.00000	H-3 → L (37%)
Chlab	798.17	0.03020	H → L (49%)	Chlbb	730.86	0.00000	H-3 → L (39%)
Chlan	683.07	0.0486	H → L + 3 (46%)	Chlbn	758.54	0.00000	H-3 → L (41%)
Chlap	795.63	0.00048	H → L + 2 (92%)	Chlbp	743.34	0.02130	H-4 → L (44%)
Chlav	775.77	0.00050	H-5 → L (72%)	Chlbv	742.37	0.00050	H-3 → L (42%)
Chlax	791.11	0.00000	H → L (98%)	Chlbx	734.85	0.00048	H-3 → L (40%)
Chlaz	795.09	0.00040	H-3 → L (41%)	Chlbz	791.77	0.00035	H-2 → L (54%)

## Conclusions

The chemical reactivity, antioxidant properties of carotenoids and chlorophyll has been studied by DFT. Results indicated high stability and lower reactivity for carotenoids and chlorophyll complexes. Lower proton affinities (PAs) than the bond dissociation enthalpies (BDEs) indicate that the direct antioxidant activity in these carotenoids is due to the SPLET mechanism with dominant solvent effects. Higher HL gap was observed for carotenoid in solvent (water) as compared to the HL gap in gas phase. Stabilized intermolecular and intramolecular interaction has been visualized between chlorophyll-carotenoid interactions. A good coorelation for reaction rate constant with temperature by Transition-State Theory (TST) was estimated for carotenoid-chlorophyll complexes in gas phase. TDDFT results showed that all the carotenoid-chlorophyll complexes have absorption spectra in visible region. Further the lower S_1_–T_1_ energy gap implicated ISC transition from singlet to triplet state.

## Supplementary Information

Supplementary Information.

## Data Availability

The optimized structure (CIF) coordinates of all fourteen complexes are given in Supplementary Information.
