# Supplementary material for: Physicochemical, antioxidant properties of carotenoids and its optoelectronic and interaction studies with chlorophyll pigments
Source: Sci Rep. 2021 Sep 15;11:18365. doi: 10.1038/s41598-021-97747-w (PMC8443628; doi:10.1038/s41598-021-97747-w)
Supplement: Supplementary file 1 — Supplementary Information. [file 41598_2021_97747_MOESM1_ESM.docx]

Supplementary Information

**Title: Physicochemical, Antioxidant properties of Carotenoids and its Optoelectronic and Interaction studies with Chlorophyll Pigments**

**Submitted by: Ruby Srivastava**

| 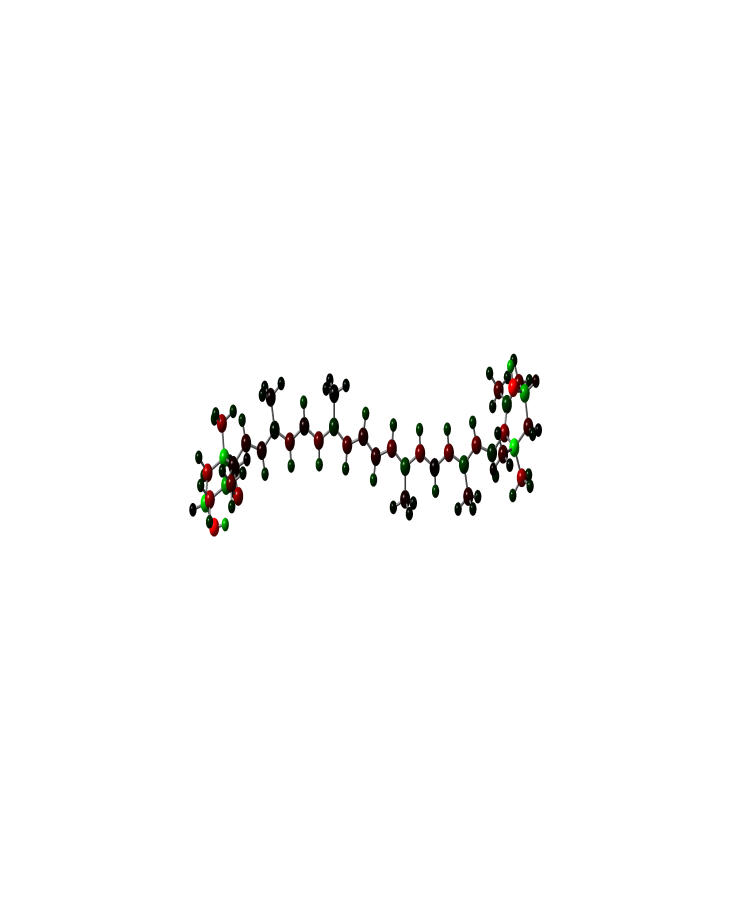 | 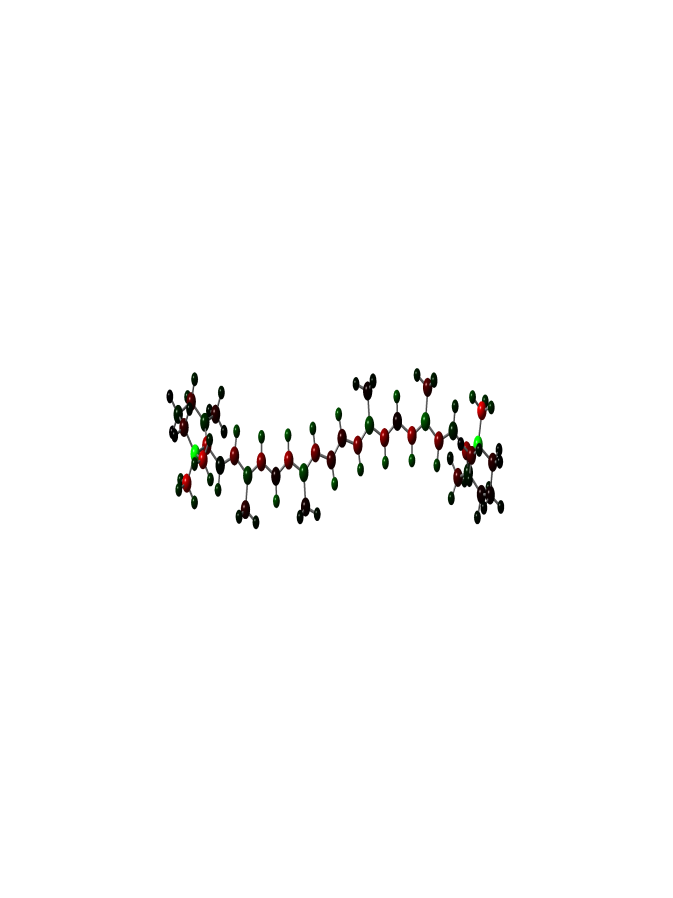 | 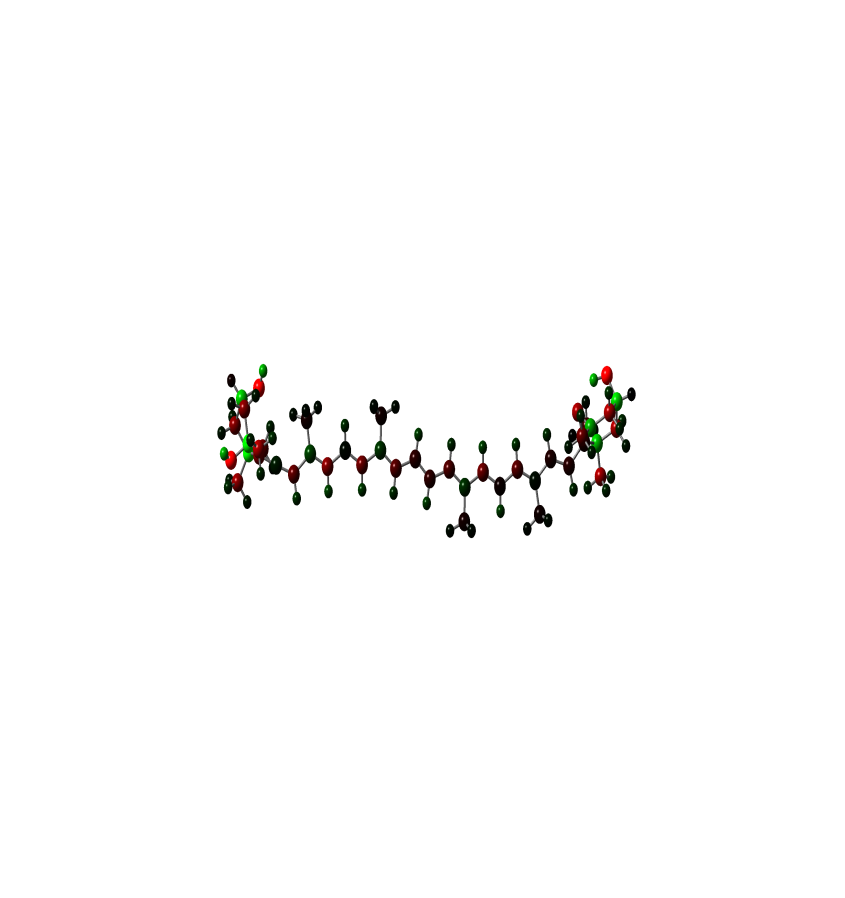 |
| --- | --- | --- |
| Antheraxanthin (a) | β-carotene (b) | Neoxanthin (n) |
| 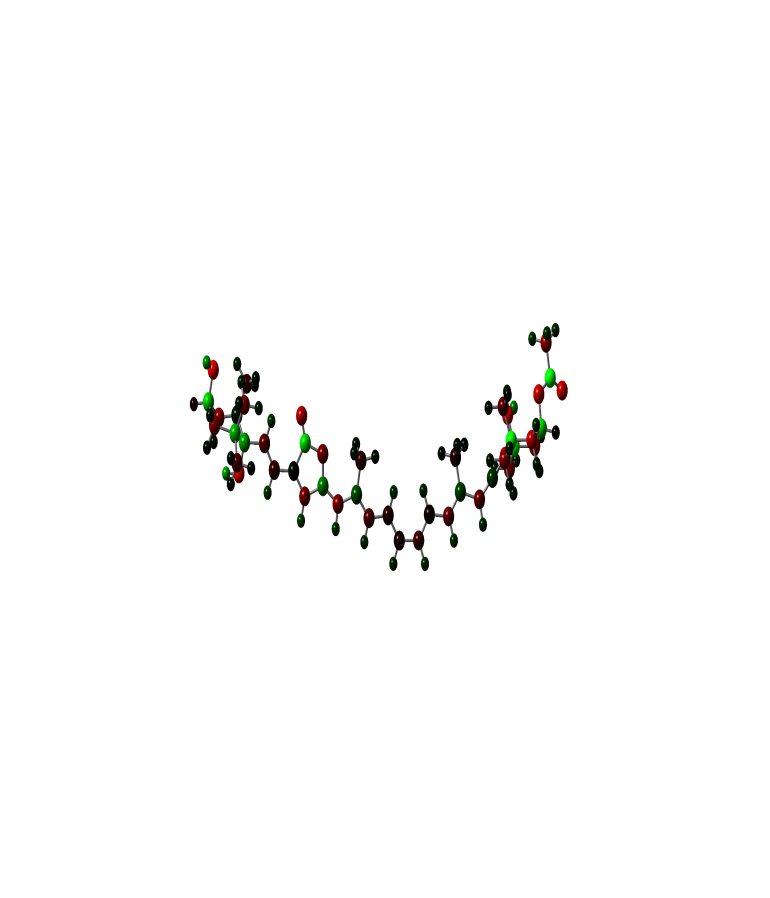 | 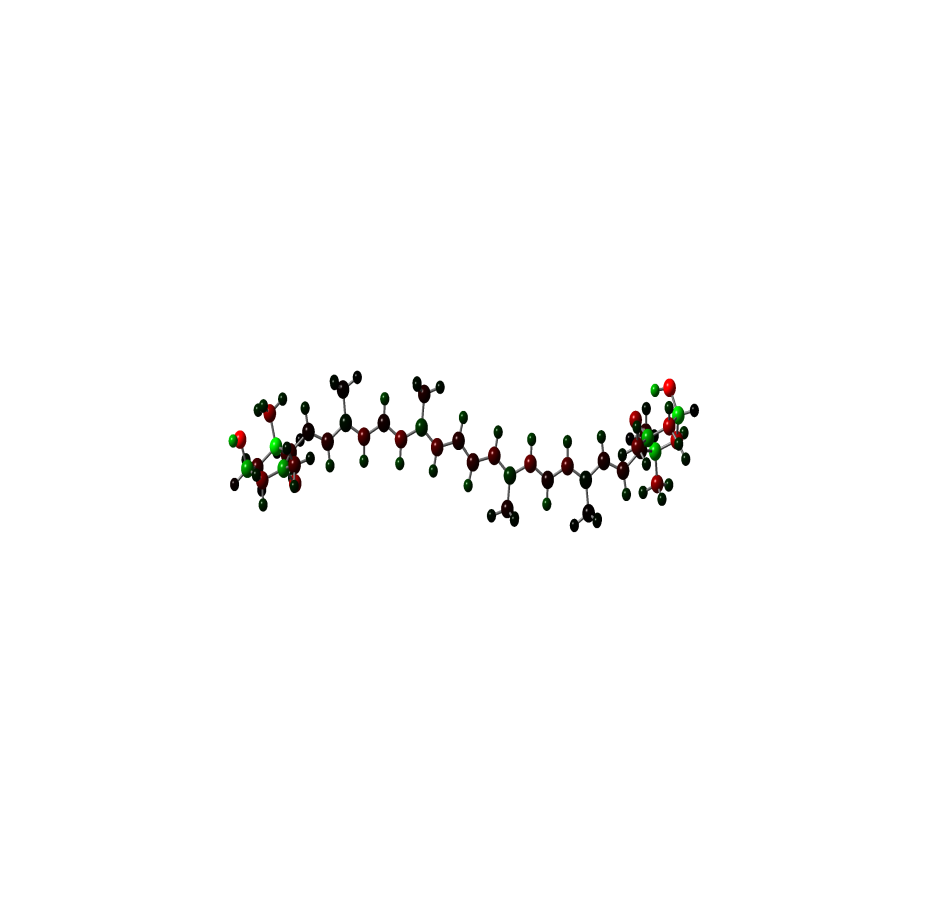 | 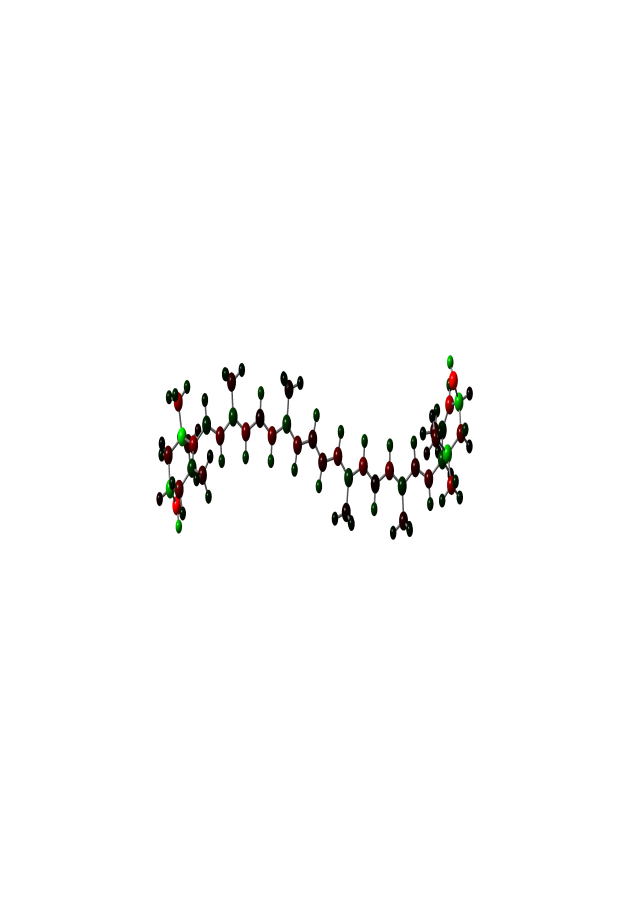 |
| Peridinin (p) | Violaxanthin (v) | Xanthrophyll (x) |
| 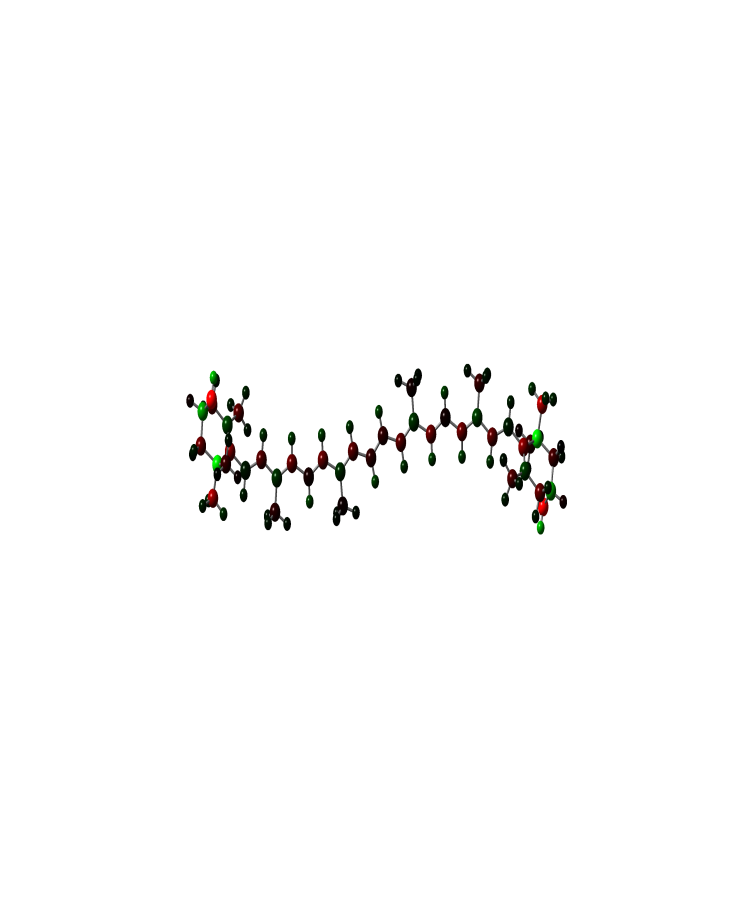 |  |  |
| Zeaxanthin (z) |  |  |

Supplementary Figure 1: Electrostatic potential (ESP) representation of Carortenoids.

|  |  |  |  |
| --- | --- | --- | --- |
| Complexes | CAM-B3LYP/6-31G** | wB97xD/6-31G** | M062x/6-31G** |
|  |  |  |  |
| Antheraxanthin (a) | -1782.218258 | -1782.583772 | -1782.520709 |
|  |  |  |  |
| β-carotene (b) | -1556.66403 | -1557.002699 | -1556.971397 |
|  |  |  |  |
| Neoxanthin (n) | -1857.384508 | -1857.756746 | -1857.688732 |
|  |  |  |  |
| Peridinin (p) | -2041.219081 | -2041.614221 | -2041.50911 |
|  |  |  |  |
| Violaxanthin (v) | -1857.392338 | -1857.774599 | -1857.693371 |
|  |  |  |  |
| Xanthrophyll (x) | -1707.031292 | -1707.379120 | -1707.336498 |
|  |  |  |  |
| Zeaxanthin (z) | -1707.039274 | -1707.387636 | -1707.342937 |
|  | CAM-B3LYP/Lanl2dz:6-31G** | wB97xD/Lanl2dz:6-31G** | M062x/Lanl2dz:6-31G** |
|  |  |  |  |
| Chlorophyll (Chl) *a* | -2733.409926 | -2933.207051 | -2799.20877 |
|  |  |  |  |
| Chlorophyll (Chl) *b* | -2807.395953 | -3007.233259 | -2907.99754 |
|  |  |  |  |

Supplementary Table 1: Optimized energy values (HF) of Seven Carotenoid, Chla and Chlb from three different basis sets.

**Formulaes:**

Proton affinity for carotenoids are calculated by,

*P(A) = -ΔE_elec_ - ΔZPE+5/2RT*

Global reactivity descriptors:

Electronegativity $x=\frac{-1}{2}\left( I+A \right)$ (1)

Global Hardness *η* = $\frac{1}{2}\left( I-A \right)$(2)

Electrophilicity *ω =* $\frac{\mu^{2}}{2\eta}$(3)

Softness S=1/2η (4)

Optimized structures (CIF)

Chlaa

C 2.00150900 -4.14041100 -2.09460200

C 3.45448700 -4.16544200 -2.10958500

C 3.87881200 -2.89984100 -1.74834100

C 2.68256800 -2.11396300 -1.51336900

N 1.55887900 -2.86942700 -1.72846800

C -2.26407200 -4.42844000 -2.13523900

C -2.40048600 -5.81316400 -2.44199800

C -1.11932500 -6.33875900 -2.60165700

C -0.22989000 -5.21810400 -2.37230900

N -0.97674900 -4.05754100 -2.08206800

C -0.55732500 0.66928000 -0.61548900

C 0.27085200 1.83309200 -0.29319500

C 1.58388800 1.43552200 -0.43717400

C 1.56411800 0.03088600 -0.86320000

N 0.23429100 -0.39545800 -0.95439100

C -2.81113300 -0.41074000 -0.87776500

C -4.32562200 -0.25245500 -0.87541700

C -4.83673200 -1.70810100 -0.97984000

C -3.59149400 -2.46503700 -1.45301300

N -2.44663800 -1.67049100 -1.26902800

C 1.16582400 -5.22473700 -2.38423100

C -3.49870700 -3.76467800 -1.89078500

C -1.96370700 0.65111300 -0.58270600

H -2.44726700 1.57808100 -0.29767000

C 2.67085900 -0.75458300 -1.11053000

H 3.64455600 -0.29627400 -0.97199700

H 1.65025500 -6.16583800 -2.62943000

Mg -0.39362200 -2.22812100 -1.52498400

C -0.26084800 3.18589400 0.07429800

H -1.23383500 3.37200400 -0.39060900

H -0.39068700 3.29413000 1.15949700

H 0.42271700 3.97522900 -0.25463100

C 5.28600400 -2.39276400 -1.61388500

H 6.01153200 -3.20029800 -1.75003100

H 5.51169200 -1.62314500 -2.36318400

H 5.46340200 -1.95050700 -0.62612800

C -0.72814400 -7.74995200 -2.91852700

H -0.13285100 -7.80355800 -3.83810500

H -0.12783200 -8.18840000 -2.11207600

H -1.61716800 -8.37055300 -3.05248100

C -4.92021100 0.51888400 0.30803900

H -4.57596600 1.55808400 0.33507900

H -6.01432500 0.53873400 0.23337900

H -4.65396700 0.04329400 1.25831800

C 4.54386900 -5.43482700 -4.01754700

C 4.30231400 -5.34674300 -2.49419300

H 5.15953000 -6.30694800 -4.26710600

H 3.59591700 -5.51736200 -4.56098300

H 5.05705700 -4.53760900 -4.38111000

H 3.83437500 -6.27712400 -2.14988800

H 5.27015300 -5.28807200 -1.98269500

C 2.81377200 2.20799300 -0.25054300

C 2.98009300 3.27614500 0.55664300

H 3.67434200 1.87265100 -0.82847600

H 3.93572000 3.78968600 0.61057000

H 2.18832700 3.65447900 1.19468800

C -3.80896800 -6.16013800 -2.43244400

C -4.59124800 -4.80965700 -2.13960100

O -4.37457100 -7.24983300 -2.61504900

C -5.44514300 -4.56349400 -3.36804500

O -5.06664500 -4.04925600 -4.42625500

O -6.71476100 -5.04203300 -3.19294200

C -7.59336600 -5.04191800 -4.36225100

H -7.74753300 -4.02092000 -4.72280200

H -7.14906500 -5.64232300 -5.15997400

H -8.52724100 -5.48371000 -4.01718900

C -7.70255000 -2.01587800 0.15504300

O -7.29393000 -2.32679900 1.28409100

O -8.60128400 -1.00003000 -0.04290200

C -7.26485300 -2.64431500 -1.14284300

C -6.14147200 -1.81093200 -1.80690700

H -6.53744000 -0.80054900 -1.96503000

H -5.93442100 -2.20786500 -2.80343900

H -8.12276500 -2.69330000 -1.81860600

H -6.93492700 -3.66213300 -0.93390400

C -9.09271600 -0.28447300 1.16012300

H -8.21538300 0.08674000 1.70236200

H -9.60122200 -1.00762000 1.80043700

C -9.97346600 0.82740600 0.68593800

C -11.28502300 0.99727200 0.95143400

C -12.12498400 0.06156100 1.79220600

H -12.59686000 0.61295300 2.61569900

H -12.93451600 -0.37066900 1.19219400

H -11.55717700 -0.76454400 2.22465500

H -9.47064400 1.56421000 0.06076000

H -5.25145000 -4.98484800 -1.28639800

C -12.02617500 2.19656000 0.38803900

C -13.08662900 1.81599100 -0.66883900

C -13.73406500 3.03617900 -1.34802500

C -14.48624700 4.01175300 -0.41583500

C -15.59803900 3.30131600 0.37455400

H -12.51498500 2.73683100 1.21120700

H -11.31054000 2.89202200 -0.06925500

H -13.86419300 1.19077100 -0.21365900

H -12.60348200 1.19896000 -1.43838400

H -12.95349500 3.59664800 -1.88451100

H -14.43877700 2.67848200 -2.11414300

H -13.76791600 4.42777300 0.30784700

H -16.33775800 2.85994100 -0.30683500

H -15.19710100 2.49653200 1.00099200

H -16.12317300 3.99773600 1.03644100

C -15.02987500 5.19635300 -1.24595800

C -15.71785200 6.31552800 -0.42793500

C -17.25632100 6.27196600 -0.48455500

C -17.96631900 7.41378400 0.27266200

C -17.71881200 7.33121000 1.79029100

C -19.47519800 7.40093300 -0.05286700

C -20.25937400 8.58482000 0.54225700

C -21.65034400 8.84729900 -0.07190200

C -22.76403200 7.79205700 0.15551500

C -22.85903600 7.34820200 1.62580500

H -14.18823900 5.62090800 -1.81102000

H -15.73469000 4.80739900 -1.99710200

H -15.37504600 6.26130600 0.61385700

H -15.39457900 7.29677300 -0.80303300

H -17.55899000 6.31143700 -1.54175000

H -17.61824600 5.30826500 -0.09816300

H -17.55260300 8.36937700 -0.09105700

H -18.12870700 6.39536700 2.19492200

H -16.65050400 7.36202600 2.02973900

H -18.19492600 8.16243000 2.32069600

H -19.89799100 6.45005400 0.30088100

H -19.59983100 7.40849200 -1.14590700

H -19.65997800 9.49675100 0.39669400

H -20.36025400 8.46105900 1.62764700

H -21.53682000 9.00621200 -1.15500200

H -22.01227100 9.80064000 0.34165800

H -23.70897500 8.30046300 -0.09222800

H -21.97781600 6.76731300 1.92413300

H -22.93705500 8.21073000 2.29956200

H -23.74103100 6.71553400 1.78392400

C -22.66742100 6.57271800 -0.78183500

H -22.60346400 6.88597700 -1.83128500

H -23.55491500 5.93618600 -0.67486500

H -21.79022300 5.95677100 -0.56150200

H -5.04372000 -2.06607900 0.04057200

H -4.59052700 0.27696800 -1.80379900

C 0.31081000 -3.18408100 3.32252000

C 1.63891300 -3.05004600 3.13002600

H -0.34814000 -2.32175600 3.27200000

C 2.34731000 -1.79586000 2.84351900

H 2.24764300 -3.95058100 3.19680000

C 3.69754800 -1.85383700 2.66327100

C 4.60193900 -0.75645700 2.36747000

H 4.16945700 -2.83459100 2.74414300

C 5.94147600 -0.93788200 2.20778100

H 4.18478200 0.24280600 2.27251800

C 6.92680000 0.10180800 1.91918800

H 6.33653900 -1.95084600 2.30545900

C 8.23620100 -0.26449700 1.77341200

C 9.36813800 0.59272900 1.48796300

H 8.47282200 -1.32443000 1.88279500

C 10.64463700 0.13026500 1.36349300

H 9.19489300 1.66061100 1.36609900

C 11.77635900 0.98602800 1.07346300

H 10.81821500 -0.93729600 1.48799800

H 11.53957000 2.04497000 0.95573400

C 13.08599900 0.61889800 0.93174300

C 14.07037500 1.65560600 0.63075800

C 15.40654500 1.46611300 0.45660800

H 13.67889400 2.67008400 0.53907700

C 16.32124500 2.55134600 0.15173000

H 15.81323500 0.46252000 0.54548800

H 15.85979100 3.53713400 0.07032700

C 17.67023100 2.47601100 -0.04269600

C 18.39798300 3.71238400 -0.35317800

C 19.73071300 3.81683000 -0.56850700

H 17.79150200 4.61634000 -0.39263000

H 20.33733300 2.91970700 -0.46712100

C 1.53588200 -0.52515500 2.75984700

H 2.15165800 0.35441700 2.56586600

H 0.98670100 -0.35376700 3.69446400

H 0.79586900 -0.59179200 1.95236600

C 6.44004100 1.52856000 1.80705200

H 7.24195200 2.22536900 1.55839800

H 5.98953900 1.85807900 2.75170500

H 5.66499400 1.61754200 1.03623100

C 13.57479700 -0.80534300 1.06762400

H 12.76891700 -1.50474400 1.29453600

H 14.05647200 -1.13998600 0.14047700

H 14.32005700 -0.88400100 1.86881600

C 18.45825500 1.18901900 0.03919600

H 19.22846200 1.25576500 0.81786200

H 18.97250500 0.98714300 -0.90858900

H 17.82959800 0.32700700 0.26786000

C -1.42589300 -6.08137300 5.38707900

C -0.39640900 -5.03210900 5.00686800

C -0.34965000 -4.49729600 3.59294400

C -1.36359200 -4.97217700 2.51956600

C -2.36194000 -6.03719100 3.05744700

C -1.86528000 -6.93970700 4.19321000

H -2.29903000 -5.58126500 5.82563500

H -2.68067000 -6.66809800 2.22003000

C 20.46287100 5.06695600 -0.87946800

C 20.01689700 5.96565200 -1.79525900

C 20.72937300 7.27636500 -2.07219700

C 21.73457600 7.68775700 -0.99798200

C 22.57449800 6.48662200 -0.58732400

C 21.76939500 5.26462700 -0.08219300

H 21.25128700 7.20464500 -3.03901800

H 22.39474800 8.47200400 -1.39938600

H 23.15956600 6.19175300 -1.46982500

C 18.80607600 5.77156400 -2.68324300

H 18.49199200 4.72816300 -2.74383900

H 19.03091800 6.12510500 -3.69878400

H 17.94810500 6.36019200 -2.32850200

C 21.43316800 5.42875000 1.42013000

H 22.35738400 5.47606700 2.01098400

H 20.84206700 4.57943300 1.78144600

H 20.87367300 6.35117800 1.58614800

C 22.69303900 4.02967000 -0.24415700

H 22.82614800 3.76288800 -1.29990800

H 22.30958400 3.15389900 0.28937700

H 23.68128100 4.25362000 0.17651100

C 0.18522400 -4.26266600 6.16641400

H -0.60984800 -3.74480400 6.71645900

H 0.91768400 -3.52582300 5.83335400

H 0.67886100 -4.95423800 6.85864300

C -0.55945700 -5.54322600 1.32987900

H -1.23240700 -5.74445300 0.48852200

H 0.20524000 -4.83529500 0.99020600

H -0.06628600 -6.48065500 1.59855700

C -2.20351800 -3.77525000 2.01853600

H -2.69817800 -3.24884100 2.84503500

H -2.98476900 -4.14117200 1.34245000

H -1.59932800 -3.05228800 1.46104700

H 23.28740600 6.78943700 0.18833900

H -0.99684300 -6.73266200 6.15833500

H -2.68655200 -7.59404400 4.50050900

H -3.26017000 -5.52562700 3.43152000

O 21.07479900 8.20757300 0.19757900

H 20.49526300 8.96136100 -0.02820300

O -0.81252000 -7.85479200 3.78668900

H 0.02343000 -7.34018700 3.71218000

H 19.97684900 8.06990800 -2.20379500

O 0.62293900 -5.55578900 4.02771700

Chlab

C 2.54369800 -3.77651700 -1.69711100

C 3.95564900 -3.52402200 -1.93280200

C 4.18354600 -2.19995800 -1.60239400

C 2.91581000 -1.66824700 -1.13857000

N 1.93516900 -2.61936500 -1.20895500

C -1.59858500 -4.76911200 -1.40691400

C -1.54387800 -6.13409400 -1.81160800

C -0.21025600 -6.44071500 -2.07997200

C 0.50911000 -5.21256000 -1.81392500

N -0.38126800 -4.20996300 -1.37902900

C -0.60688200 0.43794000 0.32497000

C 0.06398200 1.67703200 0.73926000

C 1.40139700 1.51218600 0.45411700

C 1.55225100 0.18052000 -0.14894300

N 0.30402400 -0.45059100 -0.17018900

C -2.69361700 -0.91876400 -0.00581700

C -4.21580900 -0.94953500 -0.07691800

C -4.52836900 -2.45768700 -0.20987100

C -3.18462800 -3.03431800 -0.66319300

N -2.15753200 -2.10180100 -0.43535900

C 1.88036900 -4.98172900 -1.95121900

C -2.91166900 -4.30016900 -1.11998000

C -1.99627700 0.21867900 0.38814600

H -2.59736900 1.04588600 0.74821000

C 2.72662200 -0.35813100 -0.62278000

H 3.61826800 0.25694500 -0.55847400

H 2.48518400 -5.81152800 -2.30791900

Mg -0.05110500 -2.37023600 -0.66971400

C -0.63351600 2.87120500 1.32085500

H -1.60437000 3.03401400 0.84125700

H -0.81414200 2.74606300 2.39591400

H -0.03485100 3.77675700 1.18478200

C 5.45732100 -1.41385600 -1.73272700

H 6.30811200 -2.06822600 -1.94679200

H 5.38885600 -0.68641400 -2.55078600

H 5.68897600 -0.85857500 -0.81735500

C 0.37425900 -7.74526800 -2.53185900

H 0.87949500 -7.63810400 -3.49853800

H 1.11278900 -8.11646900 -1.81138300

H -0.41000000 -8.49746200 -2.63705000

C -4.97649700 -0.26384600 1.06416300

H -4.78873200 0.81452700 1.09002500

H -6.05380500 -0.40678700 0.92158900

H -4.69555700 -0.68844800 2.03276500

C 4.90676100 -4.52034500 -4.05670500

C 4.93349000 -4.51145400 -2.51071000

H 5.62261900 -5.24666300 -4.45706700

H 3.90811500 -4.77790500 -4.42586300

H 5.16132000 -3.53019100 -4.44914500

H 4.71956800 -5.52048600 -2.13781600

H 5.94715800 -4.26996800 -2.17067800

C 2.51758400 2.43857000 0.67503500

C 2.63666700 3.29180800 1.71380400

H 3.31570100 2.41510500 -0.06568400

H 3.49274400 3.95412300 1.79768800

H 1.90042800 3.33560500 2.51056800

C -2.88441600 -6.68757100 -1.77860700

C -3.84681700 -5.47876800 -1.40703800

O -3.28918600 -7.84275800 -1.98576600

C -4.74228400 -5.30034200 -2.61882700

O -4.43320100 -4.73481700 -3.67315200

O -5.95681300 -5.90090400 -2.42744700

C -6.87652000 -5.91014300 -3.56388800

H -7.12190000 -4.88595400 -3.85828800

H -6.42005900 -6.42987800 -4.40890700

H -7.75835000 -6.44001700 -3.20966500

C -7.29952100 -3.09072400 0.93634800

O -6.99387800 -3.44299600 2.08466200

O -8.13546600 -2.03269800 0.68848500

C -6.78828100 -3.70084900 -0.34774000

C -5.80041500 -2.73386200 -1.04527700

H -6.32905500 -1.78810500 -1.21005800

H -5.54439000 -3.11089600 -2.03714600

H -7.63551700 -3.89619100 -1.01057900

H -6.31356800 -4.65272200 -0.10725700

C -8.65728400 -1.27255400 1.84819300

H -7.83837800 -1.15453600 2.56647600

H -9.43958700 -1.86990600 2.32091700

C -9.13614100 0.04396600 1.31913500

C -10.38257300 0.55384600 1.40029700

C -11.55852400 -0.12934800 2.06702800

H -11.79802500 0.37288900 3.01272400

H -12.44863400 -0.06029100 1.43154300

H -11.38392200 -1.18608900 2.28172500

H -8.36611500 0.63140600 0.82003100

H -4.46328300 -5.78320300 -0.55764400

C -10.69183300 1.90817400 0.78409700

C -11.41230700 1.77662600 -0.57899700

C -11.65069600 3.13119200 -1.27433000

C -12.44968600 4.17585900 -0.45982900

C -13.80615100 3.61909100 0.01179700

H -11.32115700 2.48606500 1.47425300

H -9.76607600 2.47804500 0.63742900

H -12.36789800 1.25347000 -0.44772000

H -10.79980700 1.14365600 -1.23338500

H -10.67679500 3.56922200 -1.53843000

H -12.17867200 2.95036400 -2.22221900

H -11.86468200 4.44018400 0.43472200

H -14.42888900 3.33631200 -0.84693700

H -13.67688900 2.73211700 0.64269700

H -14.35661600 4.35934300 0.60185500

C -12.60239500 5.46935000 -1.29580900

C -13.31113900 6.63392300 -0.56225700

C -14.80054500 6.78525100 -0.93342400

C -15.56849400 7.80406200 -0.06149600

C -15.81739100 7.24482600 1.35475100

C -16.89594000 8.20070300 -0.74594100

C -17.70981300 9.25245900 0.03108900

C -18.82964900 9.95562800 -0.76662300

C -20.00656800 9.08278500 -1.28223300

C -20.52429300 8.10398200 -0.21020600

H -11.59766400 5.78611000 -1.60698200

H -13.15039500 5.23285200 -2.22078500

H -13.20312900 6.49118800 0.52178600

H -12.80743800 7.58150400 -0.79429900

H -14.86095500 7.09173900 -1.98776100

H -15.30572400 5.81152000 -0.86762900

H -14.94993200 8.71171100 0.02807300

H -16.52791400 6.40831700 1.31097400

H -14.89182300 6.87878300 1.81261900

H -16.23356900 8.00653500 2.02203300

H -17.49465700 7.28946800 -0.88730200

H -16.67576400 8.59017700 -1.75047400

H -17.01659700 10.03039800 0.38480800

H -18.14022500 8.79698700 0.93170500

H -18.38210200 10.47942100 -1.62401000

H -19.25182900 10.73355700 -0.11413900

H -20.82399700 9.78409700 -1.50636300

H -19.78287200 7.32437700 0.00366900

H -20.74776600 8.62453800 0.72885900

H -21.44063000 7.60773400 -0.55050500

C -19.69455800 8.34244700 -2.60029100

H -19.32860600 9.03992200 -3.36315800

H -20.59875200 7.85917400 -2.99017700

H -18.93584100 7.56625400 -2.46182500

H -4.69700200 -2.85104800 0.80252900

H -4.48848500 -0.45473200 -1.02177800

C 18.82490300 4.11501000 -0.66986500

C 17.57007200 3.88269600 -0.21583700

H 19.25975700 3.41887700 -1.38499000

C 16.72804300 2.72647900 -0.55860800

H 17.12541200 4.61002700 0.46331900

C 15.47812700 2.65253900 -0.01492900

C 14.49172400 1.59862400 -0.20025100

H 15.16797500 3.46855600 0.63993000

C 13.25263800 1.64297000 0.36052600

H 14.76713700 0.74204400 -0.81010300

C 12.21263300 0.61909100 0.23033000

H 12.99104400 2.51327700 0.96440800

C 11.00444300 0.83845200 0.83173700

C 9.84360200 -0.03404700 0.83045800

H 10.87963200 1.77569500 1.37662900

C 8.66582800 0.28879400 1.43654600

H 9.91370800 -0.99338800 0.32049200

C 7.50420400 -0.58184000 1.43144200

H 8.59445300 1.24900700 1.94487600

H 7.64493600 -1.54084100 0.92946100

C 6.27259200 -0.33419600 1.97241600

C 5.23192900 -1.35504700 1.83072400

C 3.94549500 -1.24223300 2.25753800

H 5.52404100 -2.27320000 1.31893300

C 2.95106000 -2.27778600 2.04034600

H 3.63219200 -0.33261000 2.76375000

H 3.29913700 -3.15649500 1.49540900

C 1.64393700 -2.24909400 2.42839400

C 0.79977300 -3.41493200 2.11244400

C -0.42513700 -3.62751000 2.67267100

H 1.24059200 -4.12694800 1.42195100

H -0.75749300 -2.84653800 3.35194900

C 17.30415900 1.68759100 -1.49632000

H 16.60562100 0.87576100 -1.70462800

H 18.21580700 1.24909000 -1.07309000

H 17.57588700 2.14731300 -2.45439300

C 12.54202000 -0.62405500 -0.56877800

H 11.72276400 -1.34423900 -0.58212300

H 13.42228300 -1.12636000 -0.15041400

H 12.77426000 -0.36289600 -1.60844300

C 5.91275800 0.94519000 2.69715500

H 6.73665300 1.66016200 2.72235000

H 5.05649800 1.43513300 2.21691600

H 5.62349800 0.72850900 3.73245700

C 1.02035000 -1.09064000 3.17762900

H 0.84801200 -1.34690800 4.23070000

H 0.05284000 -0.82720500 2.73443500

H 1.64433700 -0.19538600 3.14628500

C 20.64121500 6.90140300 1.38030900

C 19.85622500 5.65792900 0.99185600

C 19.65449500 5.28983100 -0.30185200

C 20.25466100 6.06580100 -1.49552100

C 21.31239400 7.09019200 -1.01781700

C 20.85509700 7.87546100 0.21625600

H 21.61488800 6.58766600 1.78777700

H 21.54545300 7.76974200 -1.84785100

H 21.60021000 8.63194000 0.48763700

C -1.40087500 -4.71993700 2.52672600

C -1.12961300 -5.94592900 1.99178200

C -2.17835200 -7.03051800 1.80290900

C -3.58062500 -6.73771500 2.36291700

C -3.87269600 -5.23519200 2.38746900

C -2.79123000 -4.43156500 3.14643400

H -2.23656400 -7.22241000 0.72117300

H -4.33355600 -7.25880000 1.75975300

H -3.91104000 -4.85143800 1.35984800

C 0.24044200 -6.37569000 1.51180000

H 1.02025400 -6.11779800 2.23798900

H 0.50328400 -5.90412000 0.55907200

H 0.26616200 -7.45866700 1.35225400

C -2.75655100 -4.83615000 4.64377600

H -3.72777500 -4.63947300 5.11580200

H -1.99283500 -4.25743600 5.17817000

H -2.51397700 -5.89542600 4.77184900

C -3.18446800 -2.93430600 3.07073100

H -2.90108700 -2.49850100 2.10871400

H -2.70995800 -2.33724200 3.85798800

H -4.26927400 -2.83691400 3.19976200

C 19.37391400 4.86917700 2.19427200

H 20.16062200 4.85047900 2.95963800

H 19.10685500 3.84018300 1.94493000

H 18.49594000 5.34373400 2.65295200

C 19.11141300 6.79036200 -2.25116000

H 19.50335700 7.29333100 -3.14433900

H 18.34358500 6.07553400 -2.56771400

H 18.62633000 7.53925700 -1.61586200

C 20.95842600 5.09811100 -2.48161000

H 21.66085000 4.44127500 -1.95486600

H 21.52370200 5.67746600 -3.22194200

H 20.24417200 4.47564700 -3.03139900

H -4.85450500 -5.04599300 2.83671400

H -3.66986000 -7.14024600 3.37816000

H 20.11731900 7.40097800 2.20698000

H 19.92048200 8.40755500 0.00058000

H 22.24048100 6.55502600 -0.76961100

H -1.79413800 -7.96487300 2.23476800

Chlan

C 11.82974000 -4.49941000 0.02193900

C 13.08859600 -5.22785900 0.03587700

C 14.09614800 -4.28205800 0.04902900

C 13.44687400 -2.98375600 0.04638500

N 12.08648500 -3.12444100 0.02540200

C 7.96264000 -2.68201900 -0.05197700

C 7.15098100 -3.85233900 0.00308000

C 8.01058500 -4.96165900 0.03163700

C 9.33655600 -4.39316200 -0.00145400

N 9.26730300 -2.98313900 -0.04526500

C 12.03177600 1.14188300 0.10199700

C 13.34505800 1.79827300 0.13133300

C 14.28808400 0.79516900 0.12720900

C 13.55772500 -0.48205900 0.08198200

N 12.18357500 -0.21827700 0.07092700

C 9.52294900 1.26376200 0.07402000

C 8.27760200 2.13596000 -0.00471200

C 7.12275700 1.13674500 0.24298600

C 7.80328000 -0.21976700 0.03385400

N 9.20345700 -0.06327300 0.04023000

C 10.55939600 -5.07583900 0.01974600

C 7.22569700 -1.46223900 -0.04022000

C 10.80106500 1.81717300 0.10139700

H 10.84833000 2.89941900 0.12284100

C 14.12753400 -1.73597200 0.07228200

H 15.21083800 -1.78805400 0.09940300

H 10.51482000 -6.16086600 0.04091600

Mg 10.70339500 -1.58951500 0.00680800

C 13.55732500 3.28199300 0.12242100

H 12.75834400 3.79639300 -0.42055500

H 13.58208700 3.69653900 1.13891700

H 14.50694800 3.53562100 -0.35919200

C 15.58130200 -4.50519800 0.05757000

H 15.82066800 -5.56974100 0.13545800

H 16.05148500 -4.13117100 -0.86084000

H 16.06236100 -3.99724200 0.90246600

C 7.65729200 -6.41702400 0.06040400

H 7.50969000 -6.79494800 -0.96031300

H 8.44783700 -7.01685100 0.52235400

H 6.72265700 -6.59905800 0.59907500

C 8.24869800 3.34742000 0.93467700

H 9.05325400 4.05644300 0.71312300

H 7.29929600 3.88572800 0.82788400

H 8.34164100 3.03515600 1.98021600

C 13.21937400 -7.28220300 -1.44753300

C 13.23114200 -6.72455500 -0.00679300

H 13.32261600 -8.37352300 -1.44555000

H 12.28500800 -7.02738100 -1.95990100

H 14.04396500 -6.86106100 -2.03336800

H 12.42681300 -7.19868200 0.56874600

H 14.16691400 -7.01974400 0.48204100

C 15.74986900 0.89232800 0.12654200

C 16.48842100 1.89179900 0.64961700

H 16.27936900 0.06535600 -0.34489600

H 17.57240700 1.87501400 0.58534600

H 16.04495000 2.73065000 1.17591400

C 5.77020300 -3.44866200 0.05808500

C 5.75040300 -1.87405800 -0.04374300

O 4.72134000 -4.12218800 0.15640300

C 4.99838100 -1.55836600 -1.32509700

O 5.47167600 -1.57289400 -2.46604400

O 3.67984000 -1.29922900 -1.07422600

C 2.79778800 -1.15032000 -2.23218500

H 3.12886000 -0.31686700 -2.85802300

H 2.80412100 -2.06955800 -2.82317300

H 1.80949900 -0.96206900 -1.81496900

C 4.56945600 2.56102600 1.27758400

O 4.99086300 2.41247300 2.43475500

O 4.11382700 3.76735300 0.81524600

C 4.53719900 1.49512600 0.21323200

C 5.86254900 1.48160900 -0.58916600

H 5.99119100 2.48127600 -1.02216400

H 5.76469200 0.80199000 -1.43936500

H 3.70538500 1.69752800 -0.46580900

H 4.35917700 0.53447700 0.69754700

C 4.14361100 4.90885100 1.75887600

H 5.19097000 5.06822300 2.04416100

H 3.59069000 4.62647000 2.65677100

C 3.56556800 6.08559700 1.03633000

C 2.61565300 6.92921100 1.48747600

C 1.94355200 6.82483000 2.83921300

H 2.03055900 7.77667600 3.37907500

H 0.87239900 6.62084100 2.72357400

H 2.36461100 6.04345300 3.47526600

H 3.99018200 6.25418700 0.04770600

H 5.17366900 -1.49010600 0.80156200

C 2.15514200 8.08989300 0.62419300

C 0.65859700 8.02042800 0.24867000

C 0.24624400 9.07333000 -0.79602900

C 0.48175400 10.54757500 -0.40049000

C -0.25303500 10.91288200 0.90047500

H 2.34773600 9.03227900 1.15698000

H 2.74857700 8.11975600 -0.29875100

H 0.04014600 8.12906700 1.14801200

H 0.44578400 7.02176100 -0.15650300

H 0.79014600 8.87218000 -1.73142500

H -0.82220900 8.94248900 -1.02557700

H 1.56031000 10.69568400 -0.23539900

H -1.33631200 10.77303700 0.78591500

H 0.07822600 10.29193500 1.74060900

H -0.07605600 11.95725400 1.17808900

C 0.07259500 11.46645300 -1.57320300

C 0.37252700 12.97055900 -1.36558600

C -0.86604400 13.81072200 -1.00176400

C -0.59121100 15.31994800 -0.83445100

C 0.28372600 15.60371400 0.40074400

C -1.92172300 16.10002800 -0.77235600

C -1.75065000 17.63024700 -0.74697000

C -3.00272700 18.45510700 -1.11118500

C -4.20593500 18.43273800 -0.13292100

C -3.77732600 18.66195600 1.32706000

H 0.59277200 11.11021300 -2.47339400

H -1.00220300 11.33163400 -1.76994300

H 1.14466300 13.07910300 -0.59207800

H 0.80410000 13.38999500 -2.28520600

H -1.61448500 13.67500500 -1.79682400

H -1.32403400 13.42451600 -0.07974700

H -0.04417800 15.66338700 -1.72861800

H -0.23957400 15.30006000 1.31800900

H 1.23312400 15.05919600 0.35917100

H 0.52251700 16.66873100 0.48819000

H -2.48130600 15.76492300 0.11243800

H -2.53114000 15.82733600 -1.64678400

H -0.96383600 17.89474500 -1.47022100

H -1.37792700 17.95114000 0.23354700

H -3.35605400 18.14431600 -2.10605200

H -2.68226800 19.50287200 -1.21261300

H -4.84020700 19.28475900 -0.42359100

H -3.20273900 17.81056900 1.71174600

H -3.15429200 19.55989200 1.42462100

H -4.65425500 18.78787100 1.97387700

C -5.08482700 17.17312900 -0.25644700

H -5.39243200 17.00701700 -1.29632000

H -5.99344100 17.27887500 0.34977500

H -4.56142800 16.27519500 0.08611900

H 6.85680900 1.18764200 1.30930400

H 8.21135300 2.49925500 -1.04193900

C -19.22706800 -1.60909900 -0.21445100

C -18.02996900 -2.21648900 -0.08092100

H -19.30306800 -0.63341200 -0.68552900

C -16.73781500 -1.67757700 -0.52370900

H -18.01308000 -3.19125800 0.40477300

C -15.61930500 -2.42578700 -0.30206600

C -14.24792700 -2.09643500 -0.64872800

H -15.75426800 -3.38894100 0.19326700

C -13.20367500 -2.92564900 -0.37829700

H -14.05674300 -1.14801300 -1.14287600

C -11.79916100 -2.67239100 -0.68915900

H -13.41651100 -3.87403900 0.11809300

C -10.87579000 -3.61998100 -0.34405200

C -9.44299700 -3.57652100 -0.55098400

H -11.24630700 -4.52153400 0.14741900

C -8.60204200 -4.57969200 -0.16963200

H -9.01280100 -2.70070800 -1.03355100

C -7.16846200 -4.53868500 -0.37017700

H -9.03433100 -5.45536700 0.31203400

H -6.79244200 -3.63524500 -0.85334600

C -6.25004500 -5.49124500 -0.02452500

C -4.84045700 -5.24018900 -0.31716300

C -3.80316500 -6.07554700 -0.04028700

H -4.61867100 -4.28768300 -0.80117300

C -2.42206700 -5.75587200 -0.35519900

H -4.00552500 -7.02771400 0.44306600

H -2.26391000 -4.78917500 -0.83654700

C -1.32318000 -6.52441900 -0.11154000

C 0.00162900 -6.00495700 -0.49995300

C 1.14873700 -6.65013200 -0.33693100

H 0.01505800 -5.01154100 -0.95133800

C -16.72749200 -0.32535200 -1.19771900

H -15.72452700 -0.01072400 -1.49046700

H -17.13775500 0.44481900 -0.53199600

H -17.35012700 -0.33749600 -2.10151500

C -11.44802400 -1.37265100 -1.37660500

H -10.37749700 -1.27473200 -1.56243100

H -11.76131200 -0.51474300 -0.76852000

H -11.96285300 -1.29344700 -2.34232800

C -6.61279200 -6.79560900 0.64848300

H -7.68542000 -6.89416900 0.82142100

H -6.29341200 -7.64807900 0.03642800

H -6.10798600 -6.88380200 1.61846300

C -1.35778400 -7.88113000 0.55058800

H -0.77166800 -8.60470600 -0.02611300

H -2.37192200 -8.26932400 0.66462100

H -0.89783200 -7.82972200 1.54622600

C -22.36773600 -2.25736100 2.10669500

C -20.91379800 -2.09244900 1.70231100

C -20.51457100 -2.21051000 0.24734000

C -21.57256300 -2.46217500 -0.85837300

C -23.01772200 -2.58512700 -0.29646300

C -23.16004800 -3.13434700 1.12814900

H -22.83238000 -1.26532300 2.17643900

H -23.60219700 -3.21436200 -0.97734600

C 2.26991800 -7.31493200 -0.16410200

C 3.08178100 -7.18757300 1.13574500

C 3.26173100 -8.57495800 1.78668400

C 3.25618800 -9.75388900 0.79751200

C 3.73625400 -9.32994900 -0.58757200

C 2.84922000 -8.24262500 -1.25529200

H 4.22128900 -8.53947100 2.31396500

H 3.91123000 -10.54878900 1.18296300

H 4.75366300 -8.94283100 -0.46836200

C 2.47729400 -6.19821000 2.13496900

H 2.44812100 -5.18801400 1.71318400

H 3.09609600 -6.17696000 3.03895400

H 1.45663800 -6.48124100 2.41184600

C 1.71822300 -8.90613700 -2.06878700

H 2.14930800 -9.49505300 -2.88849700

H 1.05714300 -8.15087600 -2.50854300

H 1.13164200 -9.57410900 -1.43476400

C 3.72107400 -7.41024600 -2.22530300

H 4.51048100 -6.88081100 -1.68567300

H 3.10755900 -6.67413600 -2.75748200

H 4.18611700 -8.06891100 -2.97101800

C -20.05670400 -1.36130300 2.70517800

H -20.45691500 -0.35636300 2.88635000

H -19.02495000 -1.27009700 2.36314700

H -20.06003900 -1.90248500 3.65816100

C -21.17448000 -3.75371400 -1.60862400

H -21.81541800 -3.88254800 -2.48898500

H -20.13372400 -3.70539500 -1.94771700

H -21.29189000 -4.63668700 -0.97594800

C -21.57587200 -1.29211200 -1.86903900

H -21.70592700 -0.32236200 -1.37140000

H -22.41207800 -1.42139500 -2.56587100

H -20.65465000 -1.25979000 -2.45918800

H 3.77838400 -10.20897500 -1.24120500

H -22.40624700 -2.70981800 3.10509800

H -24.21901700 -3.11744300 1.40311300

H -23.48534400 -1.58998300 -0.29874800

O 1.90859800 -10.31379400 0.64126000

H 1.54994600 -10.58225400 1.50986800

O -22.78208400 -4.53186900 1.24662200

H -21.79895800 -4.58268200 1.23837200

H 2.47601900 -8.73118700 2.53717100

O -20.31442000 -3.37189700 1.17805400

O 4.43994300 -6.74988500 0.80701900

H 4.45804000 -5.79903000 0.52308000

Chlap

C 0.23822000 7.17855800 1.48834500

C 1.52950600 7.36771600 2.12698000

C 1.81669300 6.19415300 2.80016400

C 0.70158900 5.29693800 2.56491200

N -0.24430100 5.90144700 1.77848000

C -3.54412000 6.97785400 -0.49493300

C -3.53741700 8.24842000 -1.13920100

C -2.35314900 8.89407800 -0.78790000

C -1.66507600 7.95803500 0.07813800

N -2.43104400 6.78262100 0.22709500

C -2.34462900 2.20051800 2.14260800

C -1.68300500 1.16498000 2.94283100

C -0.49284200 1.70567700 3.38078100

C -0.42768200 3.08001600 2.86253200

N -1.57843300 3.33524000 2.10957200

C -4.27375600 2.98163500 0.73310700

C -5.66019300 2.69171400 0.17420600

C -5.91074000 3.86972700 -0.79695900

C -4.83353900 4.87826300 -0.38455500

N -3.87495100 4.25446600 0.43359300

C -0.42121300 8.12053600 0.69131700

C -4.68258000 6.18010100 -0.79837700

C -3.58802900 2.04907500 1.50538700

H -4.07602300 1.08974800 1.63051800

C 0.60369100 3.97284800 3.06648200

H 1.44077500 3.63201500 3.66678200

H 0.08679400 9.06722200 0.52882300

Mg -2.03022000 5.06119800 1.16448500

C -2.25219200 -0.19107800 3.23135500

H -3.34517200 -0.18466500 3.18421500

H -1.89443900 -0.94568100 2.51928500

H -1.96102400 -0.53269000 4.22999700

C 3.02693800 5.87075800 3.62904200

H 3.75564900 6.68610500 3.59956500

H 2.76361000 5.70601500 4.68163100

H 3.53184200 4.96457400 3.27192600

C -1.87746100 10.25178700 -1.20805600

H -1.77479700 10.92240500 -0.34598000

H -0.89982500 10.20031300 -1.70215700

H -2.58724800 10.70405500 -1.90465100

C -5.84783400 1.31597700 -0.47585800

H -5.70163000 0.50039800 0.24009500

H -6.86587400 1.22426300 -0.87328700

H -5.14506600 1.17556700 -1.30434500

C 1.94669200 9.63713500 3.18397600

C 2.34825900 8.62891500 2.08445600

H 2.55648300 10.54627200 3.12402400

H 0.89348500 9.92361300 3.08796200

H 2.08099900 9.19939500 4.17943700

H 2.25208600 9.11005900 1.10342300

H 3.41076100 8.38325900 2.19744800

C 0.54233000 1.10860700 4.22673200

C 0.80064900 -0.20754300 4.37450400

H 1.15935300 1.81133500 4.78560400

H 1.58971800 -0.53916400 5.04537400

H 0.26282900 -0.97861100 3.83244500

C -4.73607800 8.37187200 -1.94701300

C -5.57319100 7.04658000 -1.69491700

O -5.11413200 9.28651800 -2.69645800

C -6.86952800 7.50625800 -1.05665000

O -7.03666500 7.77491200 0.13823200

O -7.85742900 7.65365200 -1.99107500

C -9.10833400 8.26744100 -1.54667400

H -9.57132700 7.66290500 -0.76161000

H -8.91213700 9.27067100 -1.16022700

H -9.73685700 8.30895100 -2.43526500

C -8.02905500 3.14197200 -2.93760500

O -7.21219400 2.77733000 -3.79550700

O -9.04983100 2.33163900 -2.50951000

C -8.01697700 4.46374100 -2.21367800

C -7.39410400 4.31627700 -0.80418800

H -7.98830500 3.56953500 -0.26444500

H -7.51680900 5.25099300 -0.25195900

H -9.04627000 4.81986700 -2.11476000

H -7.46442200 5.17933900 -2.82277500

C -9.15026600 0.98745400 -3.12352900

H -8.17671900 0.49722400 -3.00250300

H -9.33096100 1.11346900 -4.19283000

C -10.24392700 0.25969400 -2.40676200

C -11.30343400 -0.36757800 -2.95630100

C -11.58939800 -0.43750500 -4.44033500

H -11.68926500 -1.48369200 -4.75764500

H -12.53884000 0.05850700 -4.67437500

H -10.81359600 0.02324200 -5.05519200

H -10.13289400 0.24541200 -1.32322100

H -5.80031600 6.60303600 -2.66766700

C -12.30988400 -1.07925100 -2.07008700

C -13.72501300 -0.46290000 -2.12825200

C -14.68925700 -1.06089700 -1.08770300

C -14.92503800 -2.58472900 -1.17851000

C -15.45777500 -2.99774500 -2.56096200

H -12.36672300 -2.13606700 -2.36807100

H -11.96160000 -1.06070600 -1.02928500

H -14.14529600 -0.58087500 -3.13441200

H -13.64240000 0.61795400 -1.95069800

H -14.30968000 -0.82642100 -0.08162300

H -15.66235100 -0.55437900 -1.17653700

H -13.96319000 -3.09601900 -1.01786800

H -16.41382100 -2.50037000 -2.77267600

H -14.75714600 -2.73152500 -3.36052300

H -15.62099800 -4.07896400 -2.61817500

C -15.86876400 -3.02692400 -0.03767900

C -16.10392200 -4.55302000 0.06761300

C -17.46132300 -5.01473000 -0.49478400

C -17.72612300 -6.53044600 -0.37725500

C -16.78248700 -7.34297100 -1.28344100

C -19.20671600 -6.83495900 -0.69138100

C -19.60334800 -8.30784800 -0.48361000

C -21.11715200 -8.59180800 -0.38939900

C -21.98354600 -8.37055100 -1.65707300

C -21.34845000 -8.98752200 -2.91571300

H -15.44713300 -2.65517400 0.90675800

H -16.83606300 -2.51575600 -0.16047100

H -15.28411200 -5.07863300 -0.44022800

H -16.05425100 -4.86131400 1.12136700

H -18.25403100 -4.47567700 0.04536700

H -17.55164400 -4.71633600 -1.54920000

H -17.53747500 -6.82633500 0.66845700

H -16.97156600 -7.10876800 -2.34023800

H -15.73022700 -7.12363300 -1.07316900

H -16.92372200 -8.42041600 -1.14893100

H -19.41107100 -6.52940800 -1.72729600

H -19.84025800 -6.20398100 -0.05045300

H -19.14607000 -8.65347400 0.45635500

H -19.16820000 -8.92872600 -1.27630500

H -21.54155400 -7.99416000 0.43151300

H -21.23098500 -9.64528200 -0.09284900

H -22.92575600 -8.90923200 -1.47069100

H -20.43983800 -8.44754800 -3.20868500

H -21.07749200 -10.03821700 -2.75276400

H -22.04508900 -8.94577900 -3.76193500

C -22.36520200 -6.89736200 -1.89885400

H -22.83036300 -6.45935200 -1.00702200

H -23.08282300 -6.81781700 -2.72529700

H -21.49432800 -6.28717900 -2.15667700

H -5.64487000 3.53451200 -1.81118000

H -6.36492700 2.77664000 1.01559900

C 2.01367700 -4.46354000 2.71761000

C 2.58296500 -3.24823100 2.57982700

H 2.61685100 -5.35032700 2.55470500

C 3.97404300 -3.01928200 2.21878000

C 4.61584900 -1.82380100 2.06850400

C 5.99952400 -2.04986800 1.70290200

H 4.18230900 -0.84103600 2.19627300

C 6.97787500 -1.13979600 1.46570300

C 8.36999000 -1.35117400 1.10518000

H 6.66083900 -0.10256200 1.56517800

C 9.14141000 -0.22926300 0.93778900

C 10.54245500 -0.17047900 0.58787500

H 8.66052300 0.73993900 1.08359300

C 11.21563400 1.00941500 0.46176800

H 11.07245800 -1.10549000 0.42945500

C 14.88137100 0.47694200 -0.58466800

H 10.65734000 1.92779000 0.64732100

H 15.19378900 1.51830200 -0.49235600

C 15.81714100 -0.43161900 -0.98256000

C 17.18400000 0.04710100 -1.26105100

C 18.19175300 -0.72369900 -1.64314400

H 17.35724200 1.11683000 -1.13508000

C 8.89284600 -2.75915600 0.93763800

H 9.94899900 -2.77343200 0.66359800

H 8.32837400 -3.29589900 0.16881400

H 8.76676100 -3.33455900 1.86007500

C 15.55626100 -1.90594400 -1.17372400

H 14.57621500 -2.21385800 -0.80505100

H 15.61697000 -2.16928800 -2.23748700

H 16.32021100 -2.50277400 -0.66276300

C -1.03136800 -5.89611800 4.32792400

C 0.28542500 -5.90866600 3.96539200

C 0.57079900 -4.67460500 3.09890900

C -0.39399400 -4.99287600 1.80593200

C -1.84029200 -5.49313600 2.20341200

C -1.98684900 -6.56705300 3.37601200

H -2.30372400 -5.89709200 1.29136600

C 19.18980100 -1.49522500 -2.01932300

C 20.08009100 -2.16172200 -0.96039800

C 21.56580800 -2.06746400 -1.35022100

C 21.89198000 -2.42487000 -2.80142100

C 20.98123000 -1.70836600 -3.79138800

C 19.45857100 -1.79571800 -3.50927200

H 22.93885000 -2.16113000 -2.99269200

H 21.27621700 -0.64940000 -3.78262300

C 19.88455000 -1.57301500 0.44100700

H 18.85414600 -1.71995900 0.77485200

H 20.10380100 -0.50115800 0.46206400

H 20.55495600 -2.07125000 1.15345200

C 18.89855700 -3.19578900 -3.86281000

H 19.12142100 -3.43152100 -4.91142100

H 17.80992700 -3.20274200 -3.73126000

H 19.32634100 -3.96959500 -3.22810700

C 18.76471500 -0.75762500 -4.41212600

H 19.09440700 0.26246100 -4.18184300

H 18.99629600 -0.96571300 -5.46382700

H 17.67700500 -0.79335200 -4.29416300

C 0.25424300 -6.05692900 0.88829100

H -0.31644100 -6.11175500 -0.04681800

H 1.28328000 -5.79329100 0.62436800

H 0.24364700 -7.05024900 1.33947400

C 1.11566100 -7.15914600 3.84923500

H 1.67683700 -7.25250700 2.91609800

H 0.48139300 -8.04001300 3.97026200

H 1.85496600 -7.15957800 4.66324800

H 21.18047700 -2.09045900 -4.79947200

H -3.03269000 -6.57816200 3.71475700

H -2.43359500 -4.62228700 2.50735100

O 21.77254900 -3.87438100 -3.07079300

O -1.58173300 -7.89642500 2.97307900

H -2.23595500 -8.28514500 2.36089500

H -1.43078000 -4.89899400 4.51431300

H 0.40972100 -3.32770300 4.57794800

C -0.54839700 -3.68983700 0.99403100

H -1.20067700 -3.85910200 0.12882900

H -0.97520600 -2.89258000 1.60671500

H 0.42252000 -3.35137500 0.61725600

O 0.02527100 -3.44811300 3.68577800

H 22.16872000 -2.68283000 -0.67373300

H 21.89174800 -1.02936800 -1.20323300

C 22.44392100 -6.14414000 -2.94523400

C 22.71196600 -4.71903700 -2.54916700

H 21.42889900 -6.42657000 -2.65045300

H 22.50812700 -6.24442000 -4.03313600

H 23.17180200 -6.80356900 -2.47168800

O 23.65426500 -4.32571200 -1.84635900

O 19.65990600 -3.57515800 -0.92785400

H 20.16539300 -4.05367900 -0.23867800

C 4.96672900 -4.08980600 1.93845600

O 4.86606000 -5.31358300 1.93950100

O 6.19160900 -3.44937300 1.62962600

C 12.61807500 1.18795500 0.11233900

C 13.49234000 0.21311400 -0.26403100

H 12.98930200 2.21179600 0.15529100

H 13.14024200 -0.81322400 -0.33739000

H 1.97750100 -2.35905100 2.73809800

Chlav

C 12.19338300 -4.35123000 -0.45118000

C 12.84624500 -5.57169700 -0.00783800

C 12.17345500 -5.99554300 1.12357400

C 11.11388100 -5.03368600 1.36164500

N 11.13754700 -4.04695600 0.41233300

C 10.52468500 -0.73895400 -2.01850000

C 11.33629000 -0.59195400 -3.17880900

C 12.24562200 -1.65040400 -3.19485100

C 11.93418900 -2.42238100 -2.01153300

N 10.85942600 -1.83043500 -1.31473100

C 7.76249700 -2.48740000 2.51183700

C 7.32684300 -3.25527800 3.68498100

C 8.17608000 -4.33476300 3.78392000

C 9.14789400 -4.22345000 2.68562500

N 8.85141700 -3.08173200 1.93098900

C 7.49557100 -0.53898500 0.94751500

C 6.77192900 0.76056600 0.62136900

C 7.30744000 1.12833400 -0.78258700

C 8.54399300 0.23330100 -0.91454800

N 8.52602800 -0.76419500 0.07885700

C 12.54843600 -3.59986600 -1.57450600

C 9.51090500 0.25315400 -1.88965000

C 7.14489100 -1.31332900 2.05048200

H 6.29511400 -0.96468100 2.62534000

C 10.17281300 -5.10647100 2.42385500

H 10.27222000 -5.95664200 3.09097300

H 13.37798400 -3.97073500 -2.17040000

Mg 9.85436100 -2.42929800 0.30825700

C 6.18818100 -2.88445700 4.58408200

H 6.02486000 -1.80229300 4.60375500

H 5.26268000 -3.37741600 4.26270600

H 6.38600900 -3.21754700 5.60875100

C 12.45370000 -7.20097900 1.97489200

H 13.25182500 -7.81267800 1.54398700

H 12.76967400 -6.91626600 2.98659300

H 11.56775400 -7.83976300 2.07694300

C 13.31297700 -1.93945700 -4.20694500

H 14.31368600 -1.84514600 -3.76705100

H 13.22508100 -2.95633700 -4.60754300

H 13.24564400 -1.23690000 -5.04098600

C 5.24132200 0.71378500 0.69743300

H 4.88755100 0.47939000 1.70690600

H 4.82024700 1.68791400 0.42047300

H 4.83749200 -0.03610900 0.00810700

C 15.38083100 -5.61222000 -0.12508500

C 14.05232400 -6.20000200 -0.65074100

H 16.24012100 -6.08611300 -0.61404700

H 15.43266100 -4.53346700 -0.30993600

H 15.47274300 -5.76756200 0.95563800

H 14.00998400 -6.07431600 -1.73961800

H 14.04514500 -7.28148800 -0.47039600

C 8.18384500 -5.40925800 4.78170200

C 7.09294900 -5.92763500 5.38339000

H 9.16230200 -5.81110500 5.04469600

H 7.19700900 -6.71252900 6.12843000

H 6.08318700 -5.61806800 5.12667500

C 10.88506500 0.56649900 -3.92463200

C 9.71503300 1.21223100 -3.06714900

O 11.27661900 1.03482200 -5.00619500

C 10.20940000 2.59488600 -2.68993300

O 10.97117100 2.86593800 -1.75508000

O 9.75025800 3.54152800 -3.56473300

C 10.29620200 4.89160200 -3.43086600

H 10.06499300 5.29998300 -2.44298700

H 11.38045200 4.86822600 -3.56586400

H 9.81869400 5.47179900 -4.21940900

C 5.35334600 3.01561900 -2.28468700

O 4.75429000 2.07467700 -2.82670000

O 4.69717000 3.99035300 -1.57865100

C 6.84658200 3.21784800 -2.27095000

C 7.46544000 2.65800600 -0.96662200

H 6.97211100 3.16596800 -0.12923300

H 8.51811100 2.94539600 -0.91005800

H 7.05785000 4.28833700 -2.33840700

H 7.26497600 2.72921700 -3.15116700

C 3.21929300 3.89132400 -1.49553300

H 2.97975200 2.91207400 -1.06512500

H 2.81822600 3.91697700 -2.51059700

C 2.74870200 5.01718900 -0.63134000

C 1.93178400 6.02906700 -0.98857700

C 1.32906700 6.21040400 -2.36382300

H 0.23909800 6.08611500 -2.31950200

H 1.51958000 7.22465900 -2.73373100

H 1.71575400 5.50888000 -3.10604200

H 3.12350100 4.98885100 0.39117500

H 8.84302400 1.31868800 -3.71740900

C 1.56186700 7.10186300 0.02026600

C 2.32510100 8.42582400 -0.21108000

C 2.03629800 9.49322600 0.85957300

C 0.57125000 9.97020400 0.97051000

C 0.06223800 10.55471300 -0.35796100

H 0.48091500 7.29175500 -0.03149200

H 1.77693500 6.74918800 1.03705000

H 2.09378300 8.82356300 -1.20680100

H 3.40094700 8.20624700 -0.20938200

H 2.35194400 9.10251900 1.83893200

H 2.66903800 10.37127700 0.65938000

H -0.06098300 9.10577700 1.22639200

H 0.67386700 11.41440500 -0.66313600

H 0.09780500 9.81446700 -1.16528200

H -0.97572900 10.89297900 -0.27348000

C 0.45105200 10.97984500 2.13447800

C -0.98115500 11.49208700 2.42011600

C -1.24990300 12.90893400 1.87964900

C -2.66435800 13.45634000 2.16458300

C -3.73979900 12.68415000 1.37809000

C -2.71558200 14.96753000 1.85399700

C -4.05468900 15.63934800 2.20801000

C -4.03077300 17.17921300 2.30668600

C -3.77138200 17.99140300 1.01080500

C -4.62033000 17.49357400 -0.17221000

H 0.85525400 10.49831600 3.03591000

H 1.10947700 11.83757400 1.92724700

H -1.70805500 10.78165000 2.00398200

H -1.15810600 11.50599800 3.50471900

H -0.51182000 13.58909700 2.33068400

H -1.06872400 12.93596700 0.79554700

H -2.86734400 13.32770800 3.24108200

H -3.59831800 12.82489500 0.29753200

H -3.69953900 11.60944000 1.58558200

H -4.74837600 13.02715500 1.63127100

H -2.49084800 15.10980200 0.78745100

H -1.90971600 15.46872800 2.41042600

H -4.38147200 15.25388800 3.18627200

H -4.82726900 15.33536300 1.49103400

H -3.28506600 17.47667500 3.05940400

H -5.00830700 17.49347900 2.70215300

H -4.09642900 19.01957500 1.23423500

H -4.29927600 16.49792200 -0.50182500

H -5.68260800 17.43261900 0.09573900

H -4.52692800 18.17159500 -1.02938600

C -2.28306400 18.08084300 0.62158700

H -1.67815700 18.44901900 1.45952400

H -2.14786800 18.77247800 -0.21964600

H -1.88086300 17.10920300 0.31944800

H 6.58228400 0.76429100 -1.52630600

H 7.13146100 1.51168300 1.34147800

C 1.44042100 -5.64756000 -0.31137500

C 0.15590000 -5.48568800 0.06708200

H 1.70382000 -5.70793700 -1.36334100

C -0.99761900 -5.37537200 -0.83516800

H -0.05139900 -5.43622300 1.13519200

C -2.23421000 -5.21964000 -0.28156300

C -3.50397000 -5.08509800 -0.97368300

H -2.29316800 -5.18781800 0.80776700

C -4.68588800 -4.93236500 -0.31767300

H -3.50075800 -5.10587900 -2.05980900

C -6.00053200 -4.78516400 -0.93781200

H -4.67008100 -4.91365000 0.77334800

C -7.09019000 -4.63815800 -0.12509700

C -8.47269800 -4.47209000 -0.52355400

H -6.91426900 -4.64187800 0.95212500

C -9.49644300 -4.32908900 0.36517800

H -8.70941600 -4.45747600 -1.58599800

C -10.87752800 -4.15684200 -0.03560700

H -9.26066600 -4.34327700 1.42796600

H -11.05055900 -4.15048700 -1.11319500

C -11.96845200 -4.00062300 0.77366000

C -13.27856500 -3.83452000 0.14835600

C -14.46093300 -3.65897000 0.79768500

H -13.28956600 -3.85245300 -0.94264200

C -15.72273800 -3.49487100 0.09723700

H -14.47031900 -3.63691600 1.88396600

H -15.65691700 -3.52787000 -0.99155800

C -16.95855900 -3.30240900 0.64090700

C -18.10097600 -3.15427900 -0.27032600

C -19.38311300 -2.95074300 0.09500700

H -17.88825200 -3.21768200 -1.33680400

H -19.65834600 -2.87970600 1.14357900

C -0.74168600 -5.43926200 -2.32283400

H -1.65647400 -5.34642800 -2.91013900

H -0.26589000 -6.38994100 -2.59539000

H -0.06226200 -4.63584700 -2.63453400

C -6.07806400 -4.80120600 -2.44746100

H -7.09941100 -4.69205700 -2.81469500

H -5.67922600 -5.74221200 -2.84630100

H -5.48062500 -3.98645600 -2.87520400

C -11.89579100 -3.98704400 2.28353300

H -10.87819100 -4.11735100 2.65451800

H -12.27738200 -3.03853100 2.68139700

H -12.51141100 -4.78979300 2.70798400

C -17.22342100 -3.22662900 2.12646400

H -17.93416800 -4.00396000 2.43412000

H -17.66842300 -2.25901900 2.39078400

H -16.31719200 -3.34954700 2.72175800

C 4.26418400 -7.30623000 1.91409000

C 2.88830100 -7.05831700 1.32119100

C 2.58088400 -5.73770900 0.64971600

C 3.66376900 -4.63594200 0.51123900

C 5.02348700 -5.04109800 1.14821400

C 4.96992000 -6.01115200 2.33279900

H 4.87912400 -7.83930700 1.17789600

H 5.55169000 -4.13129500 1.45267900

C -20.50102900 -2.78811100 -0.88168900

C -21.13177800 -4.01120600 -1.50644000

C -22.53431800 -3.96490700 -2.09274000

C -23.34763100 -2.72133200 -1.72290800

C -22.49201800 -1.46027000 -1.74745600

C -21.27363300 -1.45607100 -0.79229600

H -22.42621900 -4.01263300 -3.18382900

H -24.16661900 -2.60823600 -2.44881000

H -22.13203200 -1.33657400 -2.77624000

C -20.64520300 -5.40270900 -1.17620400

H -19.59663000 -5.39987800 -0.87658000

H -20.75360400 -6.05321000 -2.05258500

H -21.24024600 -5.83026500 -0.36006100

C -21.71855100 -1.18408400 0.66288800

H -22.35021800 -0.28876800 0.68932100

H -20.85595400 -0.99654300 1.31169600

H -22.31071200 -2.00934500 1.06315800

C -20.32563300 -0.32588500 -1.24848600

H -19.94719800 -0.52204000 -2.25749500

H -19.46751700 -0.23157300 -0.57469900

H -20.85768100 0.63352400 -1.25629600

C 2.08967300 -8.30387400 1.03030900

H 2.62957200 -8.94378200 0.32193800

H 1.11121100 -8.06676700 0.61065700

H 1.94275000 -8.87372000 1.95484000

C 3.11873600 -3.35023700 1.17385500

H 3.80816700 -2.51824700 0.98558700

H 2.13828800 -3.08287100 0.76434500

H 3.01997400 -3.46827000 2.25592300

C 3.93319600 -4.33767100 -0.98161400

H 4.18007900 -5.24833800 -1.54242300

H 4.78914800 -3.65796000 -1.06299600

H 3.07857500 -3.85278200 -1.46348700

H -23.12405300 -0.59679100 -1.51090600

H 4.15855800 -7.96024900 2.78830200

H 5.99219500 -6.23671000 2.64752800

H 5.64516300 -5.52338800 0.38033300

O -23.92735100 -2.85197500 -0.38503800

H -24.57314300 -3.58540400 -0.36201900

O 4.34158700 -5.43834100 3.51900900

H 3.36641400 -5.44172700 3.37079500

H -23.08667400 -4.86271000 -1.78674100

O 2.13192600 -5.98404200 2.06287100

O -20.15155200 -3.17984200 -2.28085300

Chlax

C -2.26711400 -4.87123200 1.67976300

C -3.68811400 -5.11621400 1.87531700

C -4.30172000 -3.87711000 1.93228700

C -3.25465400 -2.89013400 1.74219000

N -2.03941200 -3.49568000 1.59547200

C 1.97243300 -4.47616900 1.39901900

C 2.34577900 -5.85096700 1.41876500

C 1.17042200 -6.60544800 1.48450300

C 0.10849800 -5.62290000 1.52104900

N 0.64592100 -4.32155200 1.45586100

C -0.57836600 0.45054900 0.91160400

C -1.60102100 1.50505900 0.85540500

C -2.80613900 0.89732100 1.12466100

C -2.52638000 -0.52757600 1.36285600

N -1.15585900 -0.75365200 1.19882800

C 1.81453800 -0.29198500 0.73691400

C 3.28171600 0.10242600 0.61892800

C 4.00863700 -1.25907200 0.48301000

C 2.93613600 -2.25144300 0.94428700

N 1.67353500 -1.62961800 0.95602400

C -1.27064200 -5.85048300 1.61780800

C 3.06864600 -3.58706200 1.20829300

C 0.79499800 0.65835800 0.69231600

H 1.10159200 1.67918600 0.49390200

C -3.46938500 -1.48740500 1.64768300

H -4.49886900 -1.15778500 1.73966300

H -1.59408300 -6.88680200 1.67467700

Mg -0.24200900 -2.55070800 1.19607400

C -1.31483000 2.95255300 0.58362700

H -0.39163700 3.27082800 1.07926500

H -1.19557300 3.14451800 -0.49028200

H -2.12853400 3.58575400 0.94904400

C -5.75152900 -3.56478100 2.17719000

H -6.37321400 -4.46002100 2.07620400

H -5.90498400 -3.16918800 3.18863700

H -6.12799000 -2.81411100 1.47259500

C 1.03215300 -8.09930900 1.51097400

H 1.98484400 -8.57736300 1.27066600

H 0.72591400 -8.44597700 2.50504400

H 0.27986100 -8.44538600 0.79367400

C 3.61654400 1.08257100 -0.51210200

H 3.15132200 2.06190900 -0.35651700

H 4.69975600 1.22905600 -0.57457100

H 3.27067900 0.68914000 -1.47578000

C -4.30735900 -6.91529500 3.54330200

C -4.32583000 -6.46628500 2.06376700

H -4.77386000 -7.89954100 3.66029300

H -3.27989500 -6.97221500 3.91856500

H -4.85120900 -6.19823700 4.16728700

H -3.81559800 -7.21789900 1.44959000

H -5.36330500 -6.43637600 1.71071000

C -4.15260500 1.47963600 1.17101200

C -4.60388100 2.47869700 0.38425400

H -4.84096600 1.04584300 1.89465700

H -5.61576300 2.85747200 0.48958700

H -3.98889700 2.92625400 -0.39082400

C 3.78105900 -5.94519000 1.28697700

C 4.31867800 -4.45852100 1.23896500

O 4.52913000 -6.93607500 1.22343300

C 5.16448000 -4.26694100 2.48691200

O 4.76124800 -3.87626000 3.58696200

O 6.46241700 -4.63420100 2.26176000

C 7.36422500 -4.66006000 3.41140400

H 7.45114800 -3.66177100 3.84807100

H 6.98575700 -5.35470400 4.16438700

H 8.31741500 -5.00056600 3.01226800

C 6.66555700 -1.55608300 -1.02582000

O 6.67531500 -2.33286600 -1.99301500

O 6.77168800 -0.20004700 -1.17043400

C 6.53718100 -1.96971500 0.42159200

C 5.39702500 -1.23987800 1.17053000

H 5.69968400 -0.19119600 1.26519400

H 5.32064700 -1.63884700 2.18729500

H 7.48338600 -1.73533000 0.92337900

H 6.43488800 -3.05264000 0.43414700

C 6.91628100 0.34840400 -2.54534600

H 6.30009200 -0.25104100 -3.21899900

H 7.96562300 0.21266700 -2.82795300

C 6.48976400 1.78849800 -2.52532100

C 7.10850100 2.76367100 -1.82683400

C 8.33959500 2.52070500 -0.98046700

H 9.01427000 3.38353700 -1.02402900

H 8.05655200 2.37264800 0.06843300

H 8.89068800 1.62891600 -1.29006600

H 5.58705900 2.02992900 -3.08134600

H 4.94242000 -4.37573000 0.34686900

C 6.54977700 4.17206800 -1.79492200

C 6.04537400 4.56697200 -0.38729200

C 5.34694900 5.94012300 -0.35443600

C 6.18474200 7.13116300 -0.87650200

C 7.52524500 7.25822800 -0.12864500

H 7.33143300 4.87747700 -2.10897100

H 5.72239300 4.26749900 -2.50935300

H 6.88216700 4.55815000 0.32198300

H 5.33746600 3.80143000 -0.04251900

H 4.42098600 5.88123300 -0.94553700

H 5.04364700 6.15507900 0.68090600

H 6.40615600 6.95881000 -1.94094700

H 7.35592000 7.43196900 0.94197700

H 8.12867700 6.34911000 -0.23228600

H 8.12168700 8.09002300 -0.51809100

C 5.34049200 8.42568200 -0.79593700

C 6.03547100 9.68800100 -1.36209900

C 6.64434800 10.59506600 -0.27343300

C 7.51221700 11.75148400 -0.81864600

C 8.85473800 11.22688700 -1.36866900

C 7.73453400 12.81273400 0.28204600

C 8.54560400 14.03789500 -0.18020200

C 8.47154000 15.27391200 0.74236800

C 9.05999800 15.14082400 2.17409900

C 10.42639100 14.42792500 2.18168500

H 4.40118200 8.24650600 -1.33639700

H 5.06213400 8.60336500 0.25421200

H 6.81015400 9.38031400 -2.07748100

H 5.31432500 10.28488300 -1.93570100

H 5.81977200 11.01182400 0.32294200

H 7.25064800 9.99434700 0.41890500

H 6.96371700 12.23121000 -1.64551400

H 9.46903600 10.82813800 -0.55000100

H 8.70549600 10.42572200 -2.10099800

H 9.42457800 12.01981800 -1.86374900

H 8.23694100 12.32741400 1.13117300

H 6.75561000 13.15236400 0.65059400

H 8.17565200 14.34140100 -1.17110400

H 9.59677700 13.75686500 -0.32080500

H 7.42341500 15.59723100 0.82402700

H 9.00832800 16.08913800 0.23592900

H 9.22670100 16.16946200 2.52673500

H 10.32031300 13.36778500 1.92096100

H 11.11779200 14.88533500 1.46367400

H 10.88491500 14.48161700 3.17600000

C 8.09097600 14.48740600 3.18246700

H 7.11918900 14.99515600 3.17699400

H 8.49958300 14.54993500 4.19851200

H 7.92006300 13.43024700 2.95763500

H 4.15196300 -1.44905800 -0.59171700

H 3.56942400 0.56051300 1.57692500

C 4.59265900 -5.88619400 -2.18951700

C 3.30131300 -5.60576300 -1.88140100

H 5.21973600 -5.09388700 -2.59686000

C 2.60739400 -4.33130100 -2.09490400

H 2.70041700 -6.38695300 -1.42003200

C 1.28489300 -4.26801200 -1.76640000

C 0.39596200 -3.12778200 -1.89902500

H 0.82427600 -5.16909800 -1.35827100

C -0.93887400 -3.21259300 -1.63245100

H 0.81018700 -2.18488300 -2.24899000

C -1.92033000 -2.13518400 -1.77034500

H -1.33346000 -4.17449900 -1.30043700

C -3.22511400 -2.42720300 -1.49437200

C -4.34474900 -1.50841200 -1.46856400

H -3.45877300 -3.45284000 -1.20499100

C -5.60837400 -1.89925200 -1.14260600

H -4.16118800 -0.45185800 -1.65722400

C -6.71399600 -0.96930200 -1.00763200

H -5.78302600 -2.95718900 -0.95240500

H -6.46155900 0.07769800 -1.18412200

C -8.00807200 -1.25857400 -0.67321900

C -8.96718600 -0.15537300 -0.58189100

C -10.28948200 -0.26790800 -0.28202400

H -8.56675400 0.83928700 -0.78445300

C -11.18462000 0.87880800 -0.23086300

H -10.70583000 -1.25184800 -0.08202600

H -10.71753900 1.84211600 -0.44295500

C -12.52285300 0.87690700 0.03456100

C -13.24174600 2.16028500 0.00581600

C -14.56625200 2.33314800 0.20564200

H -12.63196500 3.03699800 -0.21633500

H -15.19154000 1.46540100 0.40749300

C 3.38562600 -3.17953000 -2.68744000

H 2.86097500 -2.22585800 -2.58660600

H 3.57237100 -3.35247600 -3.75439300

H 4.36697200 -3.08122600 -2.21116200

C -1.43297400 -0.77438100 -2.21265400

H -2.20332000 -0.00680400 -2.11877700

H -1.11157900 -0.80808400 -3.26131900

H -0.57481200 -0.45063300 -1.61285500

C -8.51287700 -2.66039400 -0.40292000

H -7.72788700 -3.41293600 -0.49213100

H -8.93415600 -2.73238400 0.60744300

H -9.30793000 -2.92388000 -1.11076000

C -13.32303400 -0.36943800 0.34442300

H -14.10753400 -0.51801200 -0.40780400

H -13.81704800 -0.27461000 1.31885000

H -12.70795900 -1.27030100 0.36771900

C 5.34585900 -9.68272900 -1.98505400

C 4.61779700 -8.37967700 -2.25897800

C 5.23764600 -7.19754800 -1.98866500

C 6.73618600 -7.19243400 -1.60744500

C 7.12681400 -8.41272700 -0.70337400

C 6.14044100 -9.59661300 -0.67532700

H 6.02371100 -9.93523500 -2.81470100

H 7.24912700 -8.07310100 0.33114300

C -15.30157900 3.65942200 0.10565000

C -14.61214400 4.82970200 0.81884100

C -14.75827000 6.10083200 0.39355700

C -15.57715500 6.52101800 -0.80457800

C -16.40853500 5.37107200 -1.37370700

C -15.68516600 4.00377900 -1.37985100

H -14.29812400 6.90791900 0.96492700

H -16.25632700 7.32880200 -0.49358300

H -17.32544800 5.28267800 -0.77401600

C -13.88152000 4.53122500 2.11209500

H -12.94525500 3.99199600 1.94021500

H -14.49591800 3.88957000 2.75594900

H -13.65515600 5.45321100 2.65614300

C -16.66766000 2.93806900 -1.91192700

H -16.97843800 3.18671000 -2.93374400

H -17.56942700 2.87673200 -1.28850900

H -16.19799600 1.94837200 -1.93747400

C -14.45254100 4.04407800 -2.30909900

H -14.74281100 4.44065300 -3.28780800

H -14.03853300 3.04062900 -2.44994600

H -13.66921100 4.69944400 -1.92057700

C 3.23526000 -8.54894000 -2.84869900

H 3.24638500 -9.37328000 -3.57255700

H 2.87395600 -7.64991900 -3.35367800

H 2.50844100 -8.81989700 -2.07030200

C 7.15873400 -5.91557500 -0.84519300

H 8.22339700 -5.98913400 -0.58634900

H 7.03142000 -5.00037100 -1.43449600

H 6.60019800 -5.82431500 0.09041000

C 7.53873400 -7.24866800 -2.93846700

H 7.34298900 -8.17844500 -3.48430400

H 8.61619900 -7.18551400 -2.73890300

H 7.26558100 -6.40988900 -3.59009300

H -16.25882900 3.52214400 0.63704900

H -16.70760700 5.63866400 -2.39338900

H 4.62257700 -10.50034200 -1.89847600

H 6.69142700 -10.52871000 -0.52056100

H 8.10809900 -8.77586900 -1.03522600

O -14.74132300 7.05039000 -1.89248400

H -14.07839100 7.67002600 -1.53691600

O 5.22894600 -9.53149100 0.45174300

H 4.94954500 -8.61197200 0.64967800

Chlaz

C 1.06307600 4.07164200 1.51424600

C 2.36604700 4.09394300 2.16316600

C 2.36562000 3.06839800 3.09069000

C 1.06542800 2.42575800 2.99778600

N 0.30012800 3.03009200 2.03917100

C -2.74108000 4.46454900 -0.41089200

C -2.44695800 5.55004400 -1.29018600

C -1.12326600 5.92663900 -1.06520600

C -0.65391400 5.02092800 -0.03444700

N -1.68254300 4.13370100 0.33598100

C -2.72339800 0.27622100 3.27314000

C -2.34782700 -0.64782400 4.35331600

C -1.03917600 -0.36392100 4.67697200

C -0.61462900 0.74620600 3.80613500

N -1.67442400 1.09429100 2.96806300

C -4.43578700 1.17554600 1.65811900

C -5.88848500 1.18285700 1.19632900

C -5.85590900 2.08723100 -0.05868600

C -4.52684200 2.83470100 0.10940700

N -3.71824100 2.15319300 1.03559900

C 0.62180000 4.97194300 0.53754000

C -4.06008300 3.93868000 -0.56446300

C -3.98804800 0.31072800 2.65557300

H -4.71825500 -0.40076000 3.02420200

C 0.63173600 1.34005900 3.80677700

H 1.36267600 0.95349600 4.50983700

H 1.33767800 5.71740400 0.20069800

Mg -1.60093600 2.41942500 1.41113000

C -3.26818500 -1.65091500 4.98156400

H -4.31005500 -1.31936100 4.93602400

H -3.21315400 -2.62782400 4.48231800

H -3.01184400 -1.80704600 6.03458200

C 3.45957700 2.67697900 4.04287400

H 4.37254900 3.24970700 3.85363700

H 3.17082000 2.85770500 5.08629700

H 3.71353300 1.61346300 3.95371000

C -0.34688100 7.02443100 -1.72820900

H -0.13219600 7.83846200 -1.02428100

H 0.61358400 6.66514200 -2.11630700

H -0.91763200 7.44455900 -2.55997400

C -6.52075400 -0.18980600 0.94154800

H -6.56947000 -0.79444000 1.85340600

H -7.54754400 -0.07200800 0.57298400

H -5.95230300 -0.74867900 0.18939500

C 3.31941700 6.36626100 2.77181000

C 3.45657000 5.09690000 1.90205400

H 4.12392200 7.07942900 2.55613800

H 2.36065400 6.86419000 2.58912600

H 3.36232100 6.11234400 3.83681500

H 3.46011900 5.38614500 0.84381700

H 4.43396600 4.63787700 2.09431000

C -0.18266300 -0.96509500 5.70131100

C -0.27586400 -2.21457400 6.20106600

H 0.60935100 -0.32570200 6.08937700

H 0.40666900 -2.55713400 6.97334200

H -1.00620000 -2.93268900 5.84325300

C -3.60632600 5.81702600 -2.11561400

C -4.73811500 4.81897800 -1.62111100

O -3.77531700 6.63460100 -3.03639000

C -5.85593700 5.70469100 -1.10779800

O -5.91193700 6.24128300 0.00385300

O -6.81090000 5.90352600 -2.06951000

C -7.85156100 6.88809100 -1.77765800

H -8.42171000 6.58992900 -0.89311300

H -7.39753000 7.86615100 -1.59966600

H -8.48265100 6.90816200 -2.66531900

C -8.10045900 1.47279900 -2.09282700

O -7.38214900 0.70931200 -2.75600500

O -9.32684400 1.08822300 -1.61533300

C -7.74368700 2.87477300 -1.67236200

C -7.16956000 2.88401200 -0.23382500

H -7.93299100 2.44745700 0.42150300

H -7.04212900 3.91516500 0.10350500

H -8.64376100 3.49430600 -1.70504900

H -7.02602800 3.27377700 -2.38927800

C -9.76965800 -0.29166400 -1.91955400

H -8.99998400 -0.97393500 -1.53862700

H -9.81258700 -0.40624100 -3.00438000

C -11.08766800 -0.48699600 -1.23776100

C -12.23189800 -0.93854300 -1.78972600

C -12.39196800 -1.33082800 -3.24228600

H -12.78025100 -2.35491100 -3.31659900

H -13.11772900 -0.67766800 -3.74127600

H -11.46011400 -1.28684300 -3.80967300

H -11.08206700 -0.23556000 -0.17796900

H -5.10377700 4.27312200 -2.49457500

C -13.48020600 -1.08986500 -0.93893800

C -14.63041200 -0.15178700 -1.36663100

C -15.82785300 -0.17916000 -0.39956000

C -16.51232300 -1.55046800 -0.20608200

C -17.00994400 -2.13218500 -1.54017900

H -13.82761900 -2.13168200 -0.99263500

H -13.23645400 -0.88937700 0.11233900

H -14.96626200 -0.40628100 -2.37921200

H -14.24209700 0.87446900 -1.41698300

H -15.49468000 0.18642000 0.58354400

H -16.58414800 0.53704200 -0.75528000

H -15.77622900 -2.25124100 0.21735000

H -17.74215600 -1.45968900 -2.00686600

H -16.18746000 -2.27699900 -2.24968000

H -17.49076700 -3.10523400 -1.39544300

C -17.65322400 -1.41249500 0.82681100

C -18.35503200 -2.73629700 1.21481500

C -19.72744600 -2.93443100 0.54476500

C -20.46652900 -4.22468600 0.95732900

C -19.73252400 -5.48407400 0.46081300

C -21.92348600 -4.18529700 0.44817100

C -22.78695100 -5.36849800 0.92288000

C -24.31384900 -5.18119500 0.80149000

C -24.92975800 -5.09389400 -0.61943900

C -24.40100400 -6.19519000 -1.55517100

H -17.23241900 -0.94413300 1.72759300

H -18.39932800 -0.70341000 0.43612600

H -17.69103000 -3.57784900 0.97592200

H -18.50475300 -2.76797900 2.30315400

H -20.35903600 -2.07068800 0.80112200

H -19.61646400 -2.91933000 -0.54905400

H -20.49823200 -4.25809300 2.05937300

H -19.69942300 -5.49986200 -0.63739300

H -18.70192100 -5.52428000 0.82917500

H -20.23372500 -6.39877100 0.79402700

H -21.90644900 -4.14424500 -0.65014600

H -22.39023600 -3.24844900 0.78678700

H -22.56278400 -5.54712800 1.98594100

H -22.49169600 -6.28394800 0.39541800

H -24.60815000 -4.28279800 1.36496700

H -24.78625600 -6.03310100 1.31316900

H -26.00874500 -5.26985700 -0.48713200

H -23.34129700 -6.04118600 -1.79249000

H -24.50281400 -7.18885300 -1.10098300

H -24.95476000 -6.19872300 -2.50202400

C -24.79232200 -3.70457400 -1.27148500

H -25.17783800 -2.91885500 -0.60999600

H -25.36124800 -3.66334100 -2.20898200

H -23.75080200 -3.46443100 -1.50563700

H -5.72913300 1.43435000 -0.93654000

H -6.46973000 1.69475400 1.97898000

C 1.65543600 -2.28969000 -1.37591300

C 2.93697400 -1.85735000 -1.30587000

H 1.47126100 -3.35807300 -1.28966300

C 4.12480800 -2.70890300 -1.17438900

H 3.13067200 -0.78563400 -1.33223000

C 5.34798500 -2.10651700 -1.11136700

C 6.64615900 -2.74525700 -0.99229900

H 5.37066400 -1.01607100 -1.15646700

C 7.81038700 -2.04259700 -0.94165700

H 6.68168200 -3.83003000 -0.94539800

C 9.15247100 -2.60833200 -0.82981800

H 7.75629500 -0.95367100 -0.98985200

C 10.21875400 -1.75281700 -0.79673600

C 11.62261300 -2.09363000 -0.69526200

H 10.00341200 -0.68431500 -0.85478600

C 12.61924900 -1.16351200 -0.67407600

H 11.89971000 -3.14465000 -0.63453900

C 14.02347400 -1.50403000 -0.57753800

H 12.34201900 -0.11242300 -0.73520700

H 14.23941800 -2.57244500 -0.52218200

C 15.08993500 -0.64841700 -0.55128900

C 16.43341600 -1.21410800 -0.45443700

C 17.59849400 -0.51195100 -0.42069100

H 16.48800900 -2.30298600 -0.40796400

C 18.89880400 -1.15038800 -0.32523600

H 17.56253000 0.57297600 -0.46741800

H 18.87743900 -2.24070100 -0.27913800

C 20.12328600 -0.54818100 -0.29017200

C 21.31478700 -1.39956000 -0.18935700

C 22.59793700 -0.96786600 -0.15813100

H 21.12403500 -2.47150700 -0.15798100

H 22.78059500 0.10022500 -0.25210400

C 3.92300000 -4.20566800 -1.12184800

H 4.86350600 -4.75089600 -1.03008400

H 3.41633900 -4.56217900 -2.02729500

H 3.29304600 -4.48238100 -0.26693500

C 9.28405600 -4.11271600 -0.75959800

H 10.32218000 -4.43839900 -0.68042400

H 8.85471400 -4.58160400 -1.65376600

H 8.74259800 -4.50914600 0.10838500

C 14.95722700 0.85602800 -0.61915900

H 13.91817400 1.18228800 -0.68270900

H 15.40026100 1.32415100 0.26860200

H 15.48614200 1.25232200 -1.49481700

C 20.32307500 0.94868700 -0.34943000

H 20.93069200 1.22369200 -1.22075000

H 20.85452300 1.30527100 0.54153700

H 19.38067500 1.49444900 -0.41657200

C -0.81431400 0.54477800 -2.43039900

C 0.39392900 -0.37674900 -2.36378500

C 0.45380900 -1.43293500 -1.50559800

C -0.74745700 -1.87970400 -0.63648000

C -1.98337900 -0.95585800 -0.81489600

C -1.64824100 0.49136500 -1.15810700

H -1.44498100 0.27725600 -3.28986500

H -2.59330800 -0.98970400 0.09483400

C 23.80074400 -1.82821300 -0.06741900

C 23.89267400 -2.86689300 0.80315200

C 25.09555100 -3.79033500 0.85434000

C 26.00107500 -3.71700600 -0.37360800

C 26.22622200 -2.26362900 -0.76550500

C 24.93816000 -1.45040100 -1.03988800

H 25.69101600 -3.55618700 1.75033500

H 26.96991300 -4.18401200 -0.13874700

H 26.77849100 -1.79391900 0.06055000

C 22.85805300 -3.21292200 1.85284400

H 22.17525900 -2.38672900 2.05884900

H 23.35931900 -3.48928300 2.79052000

H 22.25366900 -4.08063900 1.55235900

C 24.46284100 -1.67411500 -2.49636400

H 25.22848900 -1.32411500 -3.20126500

H 23.53921100 -1.11806900 -2.69411600

H 24.28778800 -2.73560500 -2.68126500

C 25.31685100 0.04419100 -0.87330900

H 25.50047200 0.29718100 0.17825400

H 24.54138100 0.71299700 -1.26062800

H 26.23341500 0.25524400 -1.43819900

C 1.46432300 -0.00889400 -3.36774200

H 0.99107700 0.28430500 -4.31409000

H 2.15430700 -0.83070300 -3.56699700

H 2.05554000 0.85527700 -3.03435600

C -0.34131100 -1.93139000 0.85791900

H -1.15965300 -2.35378700 1.45392300

H 0.54542300 -2.55677300 1.00511000

H -0.12664600 -0.93924900 1.26589100

C -1.18605100 -3.30557400 -1.06255500

H -1.36013600 -3.35997100 -2.14352300

H -2.12072500 -3.56980500 -0.55282900

H -0.44608700 -4.06620100 -0.79329800

H 26.87021300 -2.22179000 -1.65163100

H -0.47876100 1.57516000 -2.60452200

H -2.55844300 1.08584800 -1.25709500

H -2.60739700 -1.33322500 -1.63635400

O 25.42155600 -4.40934500 -1.52220600

H 25.22437700 -5.34047000 -1.30040600

O -0.91225200 1.14953200 -0.06443500

H 0.02785000 0.88546600 -0.05572600

H 24.74439200 -4.82443300 0.99792700

chlba

C -1.42285700 -2.84754600 1.07037200

C -2.71676900 -2.25700300 0.88813500

C -2.52782300 -1.17072000 0.01744900

C -1.11643800 -1.12575400 -0.30664100

N -0.46215700 -2.13569600 0.33635700

C 2.25889600 -4.81775600 1.95696200

C 1.78073300 -5.87963100 2.77840500

C 0.40255200 -5.74404200 2.87376500

C 0.08777700 -4.55942500 2.07993900

N 1.27273400 -4.02678300 1.52268800

C 3.01404000 -0.63239700 -1.65872400

C 2.79025800 0.51916000 -2.54239400

C 1.45109000 0.82597500 -2.46179500

C 0.84523300 -0.13269200 -1.52120200

N 1.84256800 -1.00118600 -1.05665100

C 4.53311700 -2.33286000 -0.62083100

C 5.91317800 -2.98458300 -0.55762000

C 5.82810100 -3.83038000 0.73954100

C 4.32570100 -3.93435600 0.95785700

N 3.64719300 -2.95936400 0.21230500

C -1.15309100 -3.98396200 1.86289500

C 3.66678300 -4.82756700 1.76377300

C 4.25611800 -1.26818800 -1.47048900

H 5.09029400 -0.89168600 -2.05229200

C -0.48570800 -0.18893800 -1.18108700

H -1.13296700 0.54714300 -1.64436000

H -2.00641800 -4.45064200 2.34568000

Mg 1.57055600 -2.50194400 0.25944000

C 3.86527500 1.21405600 -3.32204200

H 4.82643800 1.17255400 -2.80019200

H 4.00928900 0.76082700 -4.31172400

H 3.61305400 2.26767400 -3.47710500

C -3.53450700 -0.25953300 -0.49124900

C -0.57181000 -6.61904900 3.59850100

H -1.26098200 -6.03283000 4.21695800

H -1.17604000 -7.19891900 2.88955600

H -0.04415600 -7.32546700 4.24327400

C 6.19300300 -3.83360700 -1.81333500

H 6.15609600 -3.22237400 -2.72163500

H 7.18609400 -4.28711800 -1.73833400

H 5.45097600 -4.63502700 -1.91963600

C -4.33025100 -1.80233700 2.77614800

C -3.99346300 -2.68536300 1.55369700

H -5.25214900 -2.15232100 3.25337900

H -3.52542000 -1.82665000 3.51992400

H -4.48570000 -0.76529700 2.46396100

H -3.92564700 -3.73312900 1.86678500

H -4.82001200 -2.61438500 0.84295800

C 0.70285200 1.90158500 -3.11766200

C 0.98566900 2.45945500 -4.31181100

H -0.16550100 2.27521200 -2.57655000

H 0.37384800 3.26391700 -4.70892400

H 1.80461100 2.11680300 -4.93588500

C 2.91841800 -6.69936400 3.18204800

C 4.19887400 -6.04561500 2.52694900

O 2.96198800 -7.71167100 3.89555300

C 5.18983700 -5.74127900 3.62951000

O 5.00802800 -4.95978300 4.56944300

O 6.34774500 -6.45769300 3.48338000

C 7.32821700 -6.36237100 4.56680700

H 7.65679000 -5.32728700 4.69463100

H 6.88244700 -6.71472200 5.50020100

H 8.15391100 -7.00368800 4.26123600

C 8.81548900 -3.08269000 0.83879200

O 9.12426200 -3.81638200 -0.11228700

O 9.14596300 -1.75427300 0.87564000

C 8.02624300 -3.50137100 2.05281000

C 6.52675800 -3.14327800 1.94368500

H 6.40948100 -2.05552400 1.86509600

H 6.04746400 -3.43733500 2.88050400

H 8.44896500 -3.00378000 2.93061500

H 8.13300400 -4.58443800 2.16255500

C 9.90266900 -1.20950600 -0.27751400

H 9.32011400 -1.42435700 -1.18158000

H 10.84743400 -1.75123000 -0.35143400

C 10.06330800 0.25918700 -0.04400900

C 11.21738200 0.95586000 -0.00275800

C 12.59504200 0.35651400 -0.17905200

H 13.12907900 0.86719900 -0.99078600

H 13.19197600 0.49271100 0.73041100

H 12.58048700 -0.71058600 -0.40972200

H 9.12583700 0.79483600 0.09932900

H 4.64185200 -6.78721500 1.85534500

C 11.19206200 2.45534200 0.23232600

C 11.85659800 2.87931700 1.56079100

C 11.66461000 4.37231200 1.88364800

C 12.22324200 5.36816400 0.84345600

C 13.73025900 5.16447200 0.61257500

H 10.15383100 2.81151100 0.23069100

H 11.70317400 2.95831600 -0.60087700

H 12.92658400 2.63948000 1.53993800

H 11.42010400 2.28439600 2.37443100

H 10.58947800 4.56862300 2.01269600

H 12.13614400 4.58584100 2.85486900

H 11.70623000 5.19949500 -0.11405800

H 14.28884100 5.31055100 1.54678800

H 13.95086100 4.15611400 0.24414600

H 14.11931000 5.87283700 -0.12611100

C 11.89545000 6.81167600 1.28592200

C 12.28603800 7.91483300 0.27285300

C 13.56774800 8.68207700 0.64780700

C 13.94629800 9.81773200 -0.32689100

C 14.34248800 9.26484000 -1.70884700

C 15.07116700 10.68404500 0.27909200

C 15.37147400 11.98040800 -0.49645800

C 16.43855000 12.87762800 0.17701100

C 17.87796300 12.76308200 -0.38249300

C 17.98260400 13.32351100 -1.81274500

H 10.81572100 6.86273800 1.48438800

H 12.39090300 7.00766800 2.24938100

H 12.39015400 7.46617900 -0.72404300

H 11.46916400 8.64566800 0.19202200

H 13.42532900 9.11213700 1.65039600

H 14.41327200 7.98410000 0.73035900

H 13.05906200 10.46052500 -0.45440800

H 15.23366000 8.62769800 -1.62327200

H 13.54054300 8.66373500 -2.15033200

H 14.57159200 10.06887500 -2.41595900

H 15.98354700 10.07713700 0.36386100

H 14.78650700 10.95886500 1.30583600

H 14.43075600 12.54155600 -0.59020300

H 15.68255600 11.74295600 -1.52099000

H 16.46023500 12.65277300 1.25360200

H 16.13604600 13.93064200 0.09115900

H 18.51241600 13.39136000 0.26175700

H 17.38108300 12.73688000 -2.51788000

H 17.63356500 14.36245500 -1.85759100

H 19.02061300 13.30011600 -2.16656700

C 18.43232400 11.32907200 -0.31910500

H 18.38170000 10.92525200 0.69941600

H 19.48071100 11.30482000 -0.64099000

H 17.86866000 10.65717500 -0.97776600

H 6.27100500 -4.82283000 0.59760100

H 6.68585200 -2.21272000 -0.47906500

H -3.17488200 0.54164400 -1.15886700

O -4.76087900 -0.32320500 -0.23912100

C -17.01832000 6.00010700 0.92893100

C -16.24509600 4.96442700 0.54215300

H -16.57263900 6.89796100 1.34709300

C -14.78097500 4.90167000 0.63167600

H -16.74802900 4.08799300 0.13568100

C -14.16072100 3.76020500 0.21634400

C -12.73805600 3.46829600 0.22294900

H -14.79601500 2.95913800 -0.16576100

C -12.23302800 2.28030000 -0.20733400

H -12.05772500 4.23094900 0.59141100

C -10.82362800 1.89783600 -0.22643300

H -12.93378100 1.52757200 -0.57291600

C -10.49533500 0.65255400 -0.68632900

C -9.17513600 0.06560000 -0.78370400

H -11.31418200 0.01201000 -1.01967300

C -8.95170300 -1.19498900 -1.25337700

H -8.31483300 0.65067500 -0.46470900

C -7.62848700 -1.77696700 -1.33990800

H -9.80913400 -1.78587500 -1.57300700

H -6.81206400 -1.13791600 -1.00214400

C -7.30336300 -3.02963800 -1.78417300

C -5.89295100 -3.40947700 -1.77607700

C -5.37652400 -4.61568700 -2.13791500

H -5.20461000 -2.63221700 -1.44432600

C -3.95293000 -4.89941300 -2.08190200

H -6.04601400 -5.40132700 -2.47788500

H -3.33213500 -4.07481100 -1.72497300

C -3.31599600 -6.05792800 -2.42142100

C -1.85424800 -6.11196200 -2.29619300

C -1.07108000 -7.17107500 -2.61179700

H -1.38459600 -5.21060100 -1.90316300

H -1.55881400 -8.08352100 -2.94660900

C -14.05179400 6.10350800 1.18535100

H -12.96933800 5.96656600 1.19856300

H -14.37396300 6.31577000 2.21298800

H -14.26781000 6.99776700 0.58688900

C -9.80778300 2.90170100 0.26876800

H -8.78650600 2.52238100 0.21460600

H -10.00811200 3.17390000 1.31271400

H -9.85369900 3.82434800 -0.32344600

C -8.31230000 -4.04429400 -2.27159300

H -9.33369600 -3.66068800 -2.25233900

H -8.08893200 -4.34945500 -3.30162500

H -8.28426800 -4.94938100 -1.65145900

C -4.03132500 -7.28661900 -2.93413000

H -3.82966300 -8.15157000 -2.28953100

H -3.68477400 -7.54687900 -3.94222900

H -5.11305100 -7.15069100 -2.97857100

C -20.80049600 5.66433700 2.05134400

C -19.33859100 5.29881300 1.86632300

C -18.50830200 6.00508300 0.81550100

C -19.11323800 7.13261500 -0.06094100

C -20.61094800 7.40873300 0.25611300

C -21.43751000 6.22489200 0.77321500

H -20.88377800 6.40003100 2.86169900

H -21.08903900 7.80560700 -0.64673000

C 0.40572300 -7.23006000 -2.50761800

C 1.20934300 -6.21976800 -2.93173100

C 2.72006200 -6.25807800 -2.79364000

C 3.25058900 -7.31804800 -1.82977300

C 2.49933200 -8.62726400 -2.02601300

C 0.95999000 -8.53243500 -1.89106200

H 3.16410400 -6.42874800 -3.78646200

H 4.32129600 -7.48197100 -2.02481400

H 2.74936500 -8.98994900 -3.03304600

C 0.73075800 -4.96942900 -3.63933200

H -0.26777300 -5.08429400 -4.06462400

H 1.42582900 -4.71197600 -4.44999700

H 0.70473200 -4.10165400 -2.96302300

C 0.54151300 -8.60974100 -0.40199400

H 0.85188600 -9.57045600 0.02901400

H -0.54732300 -8.52737400 -0.30318100

H 1.01540600 -7.81111900 0.17220900

C 0.38015600 -9.77065200 -2.62283800

H 0.49553300 -9.68603400 -3.71053000

H -0.67990900 -9.92536300 -2.39755900

H 0.91265600 -10.67183400 -2.29439100

C -18.70652900 4.60474800 3.04684500

H -18.76810700 5.24015300 3.93868500

H -17.65896400 4.36467000 2.86084500

H -19.24392100 3.67340600 3.25883400

C -18.94228500 6.72238100 -1.54150600

H -19.22462600 7.55809800 -2.19301700

H -17.90114300 6.45761500 -1.75698600

H -19.57604200 5.86962000 -1.79596600

C -18.34793500 8.45611000 0.16992100

H -18.30533300 8.72391100 1.23361400

H -18.86635300 9.26675500 -0.35507900

H -17.32595500 8.41010900 -0.21896700

H 2.87444300 -9.37012000 -1.31236700

H -21.35080700 4.76811000 2.36269700

H -22.44844100 6.57844600 0.99758700

H -20.67210500 8.19470000 1.02266900

O 3.10460800 -6.91211200 -0.43385100

H 3.36154300 -5.97787600 -0.30134200

O -21.63473700 5.18308200 -0.21944900

H -20.79652600 4.67170200 -0.29282500

H 3.07599600 -5.26099300 -2.48512500

O -19.07496900 4.64286400 0.53557100

chlbb

C -1.79062600 -1.78746600 -3.44388700

C -2.73960600 -0.71066500 -3.45929400

C -1.99649500 0.46469800 -3.27853400

C -0.60484400 0.07556700 -3.16097100

N -0.49496900 -1.28023100 -3.25549500

C 0.70799500 -5.24767100 -3.38389500

C -0.26762100 -6.26189000 -3.61445300

C -1.50186400 -5.63269900 -3.73696900

C -1.21487000 -4.21274300 -3.57320000

N 0.17048800 -4.02315200 -3.36562100

C 3.71187900 -0.56860600 -2.90353700

C 4.10900300 0.84070200 -2.77573900

C 2.94945000 1.58102800 -2.78105000

C 1.83117400 0.62887200 -2.90713400

N 2.34903700 -0.67069900 -2.97314400

C 4.28642200 -3.00289800 -3.04066000

C 5.35355000 -4.08559400 -3.18143400

C 4.56527900 -5.37900100 -2.84691700

C 3.12489600 -4.93706300 -3.05944600

N 3.02976400 -3.53892700 -3.05430100

C -2.10863600 -3.15133200 -3.59950700

C 2.02924500 -5.74274600 -3.22610400

C 4.60782400 -1.65126100 -2.95967600

H 5.66540000 -1.41149100 -2.94479500

C 0.50018900 0.96373500 -2.98895800

H 0.26479700 2.02074400 -2.93897600

H -3.15692700 -3.39051300 -3.75311000

Mg 1.26459900 -2.35961100 -3.13857700

C 5.52291400 1.31448600 -2.62545200

H 6.13331500 0.58236900 -2.08745200

H 6.00026100 1.48800900 -3.59892000

H 5.55976500 2.25635700 -2.06931500

C -2.49183800 1.82582800 -3.24497700

C -2.84337000 -6.25328300 -3.97936400

H -3.53520600 -6.03905600 -3.15583900

H -3.30079700 -5.87108300 -4.89984700

H -2.75080500 -7.33784600 -4.07008900

C 5.94816100 -4.09754300 -4.60383500

H 6.42057300 -3.13875700 -4.84371500

H 6.70287000 -4.88625300 -4.68290200

H 5.16723600 -4.28330400 -5.35104500

C -4.94988700 -0.83696000 -2.24095600

C -4.22883800 -0.83151900 -3.60780800

H -6.03183400 -0.93850900 -2.38161900

H -4.60480900 -1.66505800 -1.61123400

H -4.76467100 0.10171800 -1.71035900

H -4.48278600 -1.74494400 -4.15811300

H -4.60111900 0.01824600 -4.18704500

C 2.76953200 3.02979000 -2.65304000

C 3.63610800 3.98073500 -3.05541000

H 1.84044700 3.35480500 -2.18652400

H 3.41616800 5.03334600 -2.90438200

H 4.56355800 3.74422400 -3.56702500

C 0.40754700 -7.55408000 -3.63619700

C 1.93812000 -7.26673700 -3.36163800

O -0.04520200 -8.69342300 -3.82489900

C 2.34518700 -8.04611900 -2.12732100

O 1.86856200 -7.90690800 -0.99647000

O 3.32392000 -8.96246000 -2.40735000

C 3.71405300 -9.86746600 -1.32383500

H 4.10345500 -9.29906400 -0.47476800

H 2.84838900 -10.44972500 -0.99888100

H 4.48056000 -10.51188000 -1.75226600

C 7.30599600 -6.17157600 -1.70511000

O 7.84849900 -6.31766600 -2.81021800

O 7.82555600 -5.35284600 -0.73744700

C 6.01530700 -6.81326800 -1.26506100

C 4.80521300 -5.85986500 -1.39058800

H 4.94765200 -4.99202300 -0.73522500

H 3.92603700 -6.38756800 -1.01247100

H 6.11993000 -7.12293600 -0.22078700

H 5.84928400 -7.69496800 -1.89052100

C 9.07155000 -4.62345500 -1.06177000

H 8.87863200 -4.03103000 -1.96536200

H 9.84588800 -5.35537800 -1.30066300

C 9.39275600 -3.76806300 0.12498700

C 10.60001400 -3.59805100 0.69961800

C 11.87329300 -4.27150100 0.23549900

H 12.65346400 -3.52100600 0.05335900

H 12.25730900 -4.94841400 1.00792000

H 11.74925000 -4.84905200 -0.68313300

H 8.53644000 -3.23052400 0.52949600

H 2.51981900 -7.65117000 -4.20487600

C 10.75403400 -2.66790200 1.88946400

C 11.25744900 -3.37730200 3.16528300

C 11.22137300 -2.47833300 4.41432700

C 12.04275900 -1.17273800 4.33487800

C 13.52386100 -1.44640800 4.02312000

H 9.79081900 -2.18933600 2.10869600

H 11.45619700 -1.86348400 1.62648200

H 12.27605300 -3.75256300 3.00866100

H 10.62656000 -4.25775300 3.34871000

H 10.17428800 -2.21757700 4.63012500

H 11.57871000 -3.06073200 5.27714800

H 11.63300500 -0.55281200 3.52220700

H 13.97741900 -2.06587000 4.80837600

H 13.64725000 -1.97090300 3.06879200

H 14.09483100 -0.51447000 3.95810000

C 11.86505700 -0.37638900 5.64726700

C 12.52217600 1.02467300 5.66571100

C 13.85277800 1.08316600 6.43897800

C 14.45850700 2.49544600 6.58120000

C 14.79538300 3.10976900 5.20951400

C 15.70120500 2.44703700 7.49594600

C 16.19026400 3.82387500 7.98392300

C 17.42551500 3.75195100 8.91420700

C 18.79615100 4.02931700 8.24950200

C 18.93077100 5.49878500 7.80959000

H 10.78624200 -0.27519100 5.83154800

H 12.26067800 -0.97684000 6.48088500

H 12.66885300 1.36512600 4.63204600

H 11.83332400 1.74596100 6.12796600

H 13.67982900 0.67669000 7.44662400

H 14.59316300 0.42273400 5.96487100

H 13.70510100 3.13624600 7.06934300

H 15.50378900 2.46834000 4.66715700

H 13.90276600 3.22716900 4.58604300

H 15.25137100 4.10021800 5.31266400

H 16.51215300 1.92593800 6.96805200

H 15.46616900 1.83607600 8.38043100

H 15.35905300 4.30540700 8.51814900

H 16.40953400 4.47119200 7.12590900

H 17.45588800 2.75741900 9.38330500

H 17.30677900 4.47319800 9.73479900

H 19.55902300 3.84856200 9.02258800

H 18.20728600 5.74974600 7.02417600

H 18.76142900 6.18098000 8.65181500

H 19.93281500 5.69720000 7.40993000

C 19.09470900 3.08108500 7.07489200

H 19.02696200 2.03020500 7.38158200

H 20.10499600 3.25490100 6.68435700

H 18.38951600 3.23875700 6.24974300

H 4.82731200 -6.19193100 -3.53344800

H 6.16145200 -3.91227400 -2.46305800

H -1.73068700 2.61958800 -3.16509600

O -3.69935200 2.16007900 -3.30500600

C -17.67109300 -1.02164900 5.23034000

C -16.59165900 -0.73850400 4.46257200

H -18.00096500 -0.26982700 5.94372700

C -15.77946000 0.48192900 4.53607600

H -16.28563300 -1.46105100 3.70677700

C -14.72412900 0.61126500 3.68004000

C -13.78730200 1.71630000 3.58534700

H -14.54833800 -0.20616200 2.97817600

C -12.76857700 1.74448600 2.68338500

H -13.90855600 2.55182000 4.26916000

C -11.78289100 2.81165200 2.53142100

H -12.66141900 0.89826500 2.00249300

C -10.82284800 2.68071000 1.56637000

C -9.75433000 3.60512900 1.24827300

H -10.85083900 1.77674200 0.95493600

C -8.84541200 3.38549600 0.25568000

H -9.66877600 4.52196700 1.82912900

C -7.77415800 4.30564800 -0.06395200

H -8.93276000 2.47021300 -0.32760500

H -7.73352900 5.20463500 0.55383500

C -6.82519500 4.17139200 -1.04030400

C -5.83586900 5.23632700 -1.19415500

C -4.81002600 5.25033700 -2.08844500

H -5.95014800 6.09039300 -0.52363600

C -3.87747300 6.36035300 -2.17950900

H -4.66906200 4.39936100 -2.74883700

H -4.04948600 7.17359000 -1.47117400

C -2.83039700 6.49767500 -3.04456900

C -2.01167700 7.71355000 -2.96224600

C -0.95593700 8.01454400 -3.75573400

H -2.31592000 8.43181400 -2.20205100

H -0.70630100 7.31708700 -4.55432900

C -16.15820300 1.52542600 5.56183300

H -15.49699200 2.39306800 5.53905000

H -16.12639000 1.10517000 6.57478500

H -17.18193500 1.88213700 5.39139100

C -11.88158400 4.00424000 3.45558700

H -11.10440900 4.74563400 3.26443700

H -11.79507300 3.69143600 4.50375200

H -12.85271400 4.50255900 3.34340100

C -6.75448000 2.99415000 -1.98572100

H -7.46014600 2.20468800 -1.72028200

H -6.97734600 3.31478100 -3.01155800

H -5.74964700 2.56048800 -2.00991400

C -2.47305600 5.46616000 -4.09018200

H -1.42720000 5.14907900 -3.97943700

H -2.57226200 5.88803400 -5.09880100

H -3.10677000 4.58007000 -4.03305100

C -18.76694400 -4.75905100 4.92223800

C -17.93592700 -3.49736100 5.08261400

C -18.48793700 -2.25598100 5.15737500

C -20.01638100 -2.02654900 5.17689700

C -20.78080700 -3.35846400 5.35774300

C -20.21061400 -4.48540900 4.49431600

H -18.75849600 -5.31207500 5.87506300

H -21.84170500 -3.18914400 5.13031400

H -20.81608600 -5.39452300 4.59715300

C -0.13082000 9.24423200 -3.70304500

C 0.35428300 9.77053900 -2.54571700

C 1.20077800 11.03409900 -2.50914800

C 1.79661500 11.41877900 -3.86684900

C 0.72300000 11.29692600 -4.94952400

C 0.17723400 9.85752200 -5.08850600

H 0.57990200 11.86175900 -2.12978600

H 2.18946000 12.44264500 -3.83032300

H -0.10923700 11.97017700 -4.69789000

C 0.12323800 9.17339900 -1.17262700

H -0.18810100 8.12805300 -1.21646100

H -0.64926900 9.72679600 -0.61961600

H 1.04245300 9.23767400 -0.57642200

C 1.21431300 8.97625600 -5.82904900

H 1.40581400 9.37732200 -6.83254900

H 0.85786000 7.94692200 -5.94891300

H 2.16533200 8.93654700 -5.28762200

C -1.10989000 9.93071400 -5.94165600

H -1.89455000 10.48885300 -5.41784800

H -1.50838300 8.93808500 -6.17493200

H -0.90095800 10.44184000 -6.89040900

C -16.45358600 -3.78880500 5.18870800

H -16.29983800 -4.68585800 5.80351200

H -15.89169000 -2.96467700 5.63205900

H -16.01405900 -4.00443100 4.20470000

C -20.44897100 -1.34574900 3.85604700

H -21.51609100 -1.09030800 3.88817700

H -19.88219700 -0.42335800 3.68756600

H -20.27863500 -1.99919100 2.99353700

C -20.41868600 -1.10477600 6.35451400

H -19.99396800 -1.46096000 7.30080300

H -21.51108600 -1.09630700 6.45639600

H -20.10104800 -0.06812000 6.20100300

H 1.11291600 11.62283600 -5.92328500

H 2.64406900 10.76185100 -4.09968500

H -18.26790100 -5.41462800 4.19410300

H -20.23806800 -4.20159400 3.43474600

H -20.72471800 -3.66361200 6.41306800

H 2.00236500 10.90171800 -1.76974600

Chlbn

C -9.34563300 -4.49967000 0.26093200

C -10.64424600 -4.65534500 -0.33819900

C -10.76147000 -3.62675600 -1.28238500

C -9.52260100 -2.86857800 -1.24178800

N -8.67904000 -3.41327800 -0.32184100

C -5.72705000 -4.15127200 2.49870000

C -5.87181600 -5.26594100 3.38300400

C -7.08168400 -5.88129100 3.08157400

C -7.63455400 -5.09405900 1.98323400

N -6.75606100 -4.04111300 1.65660100

C -6.05841700 -0.20459200 -1.42472600

C -6.56636700 0.70558400 -2.46590700

C -7.81965700 0.25305800 -2.80787600

C -8.08937200 -0.93342000 -1.97011600

N -6.98830400 -1.16130000 -1.14390300

C -4.27952300 -0.88244000 0.21165300

C -2.86310400 -0.70489700 0.75572900

C -2.93967500 -1.44038200 2.12064100

C -4.15726500 -2.33770500 1.92927200

N -4.92469300 -1.90065500 0.84913000

C -8.83741400 -5.28831900 1.31346100

C -4.52559200 -3.41646600 2.69503400

C -4.78669200 -0.10351900 -0.82554100

H -4.13168200 0.67619700 -1.20013200

C -9.22559200 -1.71028000 -2.02441200

H -9.98490500 -1.40664300 -2.73673600

H -9.46277100 -6.11143800 1.64826400

Mg -6.70855500 -2.79874700 0.04624100

C -5.83355100 1.90967300 -2.97608100

H -5.19172400 2.34127500 -2.20172000

H -5.19299700 1.66583800 -3.83426400

H -6.53628000 2.68368200 -3.30098500

C -11.89309600 -3.35508000 -2.15011400

C -7.70927600 -7.07229000 3.73942800

H -8.59195200 -6.77879600 4.32141000

H -8.03556500 -7.81846500 3.00546500

H -7.00227400 -7.54656200 4.42432300

C -1.82508400 -1.32254500 -0.20230800

H -1.85306200 -0.83254400 -1.18183200

H -0.81902900 -1.20802000 0.21471900

H -2.02354300 -2.39005000 -0.35917300

C -12.73763200 -5.12649300 0.99929800

C -11.67879500 -5.68321400 0.02120100

H -13.46153100 -5.90601800 1.26267900

H -12.27320500 -4.75991800 1.92201000

H -13.28397700 -4.30255200 0.53137200

H -11.19992400 -6.56475200 0.46395900

H -12.19454600 -6.00568300 -0.88819100

C -8.77915600 0.80596800 -3.76657800

C -8.48922900 1.51657100 -4.87558400

H -9.82948700 0.61601200 -3.54840400

H -9.28001700 1.89687700 -5.51549200

H -7.46979300 1.71551900 -5.18978900

C -4.69365200 -5.33671900 4.23474800

C -3.77435200 -4.11553700 3.83555300

O -4.38289600 -6.15933900 5.11220800

C -3.54818700 -3.27748600 5.07744300

O -4.42557200 -2.74087400 5.76075400

O -2.21530100 -3.18269500 5.38787700

C -1.87527300 -2.50172200 6.63819200

H -2.23221300 -1.46834700 6.61909200

H -2.33554200 -3.02347100 7.48092000

H -0.78837200 -2.54223000 6.69936100

C -0.99814000 0.84802600 2.85918500

O -0.00013500 0.43173200 2.25295200

O -1.51309500 2.10110500 2.64829800

C -1.80330200 0.07618900 3.87282600

C -3.13740600 -0.45620000 3.30339500

H -3.76331300 0.38417800 2.97985200

H -3.67711800 -0.94212700 4.11974300

H -2.01649300 0.73858000 4.71799400

H -1.19119900 -0.76260300 4.21596600

C -0.83874200 2.95569500 1.64446600

H -0.72983100 2.36350800 0.72782100

H 0.16179700 3.18962100 2.01357100

C -1.70488300 4.15862400 1.44049800

C -1.34440600 5.45330300 1.54983300

C 0.03970900 5.93491400 1.92583700

H 0.41647300 6.63428000 1.16822600

H 0.01209300 6.47974900 2.87708300

H 0.76867400 5.12815800 2.02639900

H -2.73287700 3.92455100 1.16671400

H -2.80114900 -4.50414100 3.51959100

C -2.35905900 6.54991000 1.28126100

C -2.65767700 7.43021500 2.51481200

C -3.82719200 8.40639500 2.29180400

C -3.66106800 9.40794600 1.12804300

C -2.40131200 10.27438800 1.29658600

H -3.29948900 6.10358400 0.93326300

H -1.98884800 7.18843600 0.46622200

H -1.75906000 7.98788000 2.80563600

H -2.90425300 6.77415400 3.36053800

H -4.74396900 7.82224800 2.12085600

H -3.99246700 8.97649100 3.21858800

H -3.56036200 8.83918100 0.19065800

H -2.45137300 10.85342200 2.22846000

H -1.49157300 9.66414300 1.32985300

H -2.28905500 10.98078100 0.46722800

C -4.93988200 10.26578400 1.00119100

C -4.96759700 11.22844700 -0.21020800

C -4.69242100 12.69888600 0.15636700

C -4.74284000 13.68156800 -1.03318500

C -3.58510500 13.43310700 -2.01799000

C -4.74560100 15.13596700 -0.51619000

C -4.99178500 16.20351400 -1.59874500

C -5.09050900 17.64495800 -1.04180200

C -3.80816300 18.50592000 -1.15206800

C -3.47952400 18.85640000 -2.61496100

H -5.79722300 9.58066900 0.94450800

H -5.07263900 10.84241900 1.92961900

H -4.24421700 10.88195900 -0.96037800

H -5.95258600 11.18189400 -0.69549600

H -5.44131400 13.00860700 0.90048400

H -3.71437600 12.78559300 0.65145900

H -5.69237100 13.51324700 -1.56860200

H -2.61988200 13.62411900 -1.52887300

H -3.57862700 12.40036900 -2.38248000

H -3.65181300 14.08554800 -2.89492800

H -3.79393800 15.33442700 -0.00324000

H -5.53327100 15.23380500 0.24585300

H -5.92697400 15.94804800 -2.11722600

H -4.20364700 16.15546700 -2.35976900

H -5.39230000 17.59534900 0.01480200

H -5.89364200 18.18373700 -1.56385200

H -4.02180200 19.45113700 -0.62941800

H -3.24632900 17.95778300 -3.19935000

H -4.32362400 19.36203400 -3.09997500

H -2.60906200 19.52148200 -2.67172200

C -2.59358500 17.86012800 -0.46223700

H -2.80702600 17.62730300 0.58808200

H -1.72844900 18.53400000 -0.49078000

H -2.30408000 16.92838200 -0.96300500

H -2.03932000 -2.03782600 2.30481000

H -2.63680200 0.35808500 0.88555000

H -11.77317300 -2.51408300 -2.85526600

O -12.96908000 -3.99653800 -2.15653600

C -2.03805900 -6.74902700 -2.55841500

C -0.88270000 -6.08098700 -2.36136500

H -2.03261600 -7.78413800 -2.88729100

C 0.46905600 -6.62037700 -2.55176200

H -0.95099200 -5.04345300 -2.03657600

C 1.52708000 -5.80034400 -2.29065000

C 2.94139500 -6.10932400 -2.40421900

H 1.30100100 -4.78769100 -1.95170900

C 3.91160400 -5.20680400 -2.09536400

H 3.22686900 -7.09972500 -2.74744600

C 5.35303500 -5.42675900 -2.17792700

H 3.60179300 -4.21949700 -1.74841100

C 6.18530100 -4.40507100 -1.81333900

C 7.63356000 -4.39892100 -1.81309200

H 5.71832900 -3.47734200 -1.47748300

C 8.37283400 -3.32449300 -1.41592200

H 8.15930900 -5.29308300 -2.14318600

C 9.82087700 -3.30901100 -1.41006000

H 7.84287700 -2.43315200 -1.08408200

H 10.29758300 -4.22753000 -1.75654200

C 10.64078800 -2.28603100 -1.02133100

C 12.08689600 -2.48537300 -1.09331200

C 13.03731000 -1.57877500 -0.74031000

H 12.41475500 -3.45757700 -1.46477600

C 14.46171500 -1.84692100 -0.83486100

H 12.72747700 -0.60540800 -0.36947700

H 14.72743900 -2.83505800 -1.21442800

C 15.47998800 -1.00534200 -0.50137100

C 16.86774300 -1.47478800 -0.67776000

C 17.95116700 -0.77893100 -0.36370200

H 16.98820800 -2.47573400 -1.09516700

C 0.60351700 -8.05025800 -3.01979800

H 1.64448700 -8.35640800 -3.13452400

H 0.10517300 -8.19322400 -3.98725400

H 0.13125700 -8.73827000 -2.30681500

C 5.83983500 -6.77418500 -2.65967000

H 6.92845800 -6.83726500 -2.69272000

H 5.46213900 -6.98565900 -3.66782200

H 5.47942800 -7.57476300 -2.00156900

C 10.13390600 -0.95455500 -0.51515800

H 9.04457900 -0.89932900 -0.49860500

H 10.49313600 -0.76670200 0.50417200

H 10.50071300 -0.13553700 -1.14616200

C 15.29354800 0.39539700 0.03157700

H 15.93037900 0.56268000 0.90677600

H 14.25918100 0.60467000 0.31149200

H 15.59723700 1.13400900 -0.72253400

C -5.51984400 -5.07562800 -3.42348400

C -4.01838200 -5.31018700 -3.42462200

C -3.38629900 -6.14559100 -2.33430300

C -4.24877600 -6.81612700 -1.23246300

C -5.76363000 -6.47145200 -1.34618700

C -6.11797100 -5.14264400 -2.01628400

H -6.00239100 -5.82630400 -4.06133300

H -6.20596500 -6.49519700 -0.34461000

C 19.00135400 -0.06734400 -0.01801800

C 19.64676400 0.91129200 -0.99846700

C 19.58980700 2.34601700 -0.41872200

C 19.64593900 2.40933500 1.12172500

C 20.33612400 1.18587300 1.72064800

C 19.63816400 -0.16190400 1.38509700

H 20.43134300 2.91138500 -0.84083900

H 20.18910300 3.31570900 1.42597000

H 21.35969600 1.16662200 1.33212000

C 19.04236100 0.86949300 -2.40185000

H 19.18254800 -0.12015700 -2.84605300

H 19.52546200 1.61644300 -3.04690300

H 17.97019200 1.08457900 -2.38091800

C 18.57293600 -0.48866800 2.45207000

H 19.05951800 -0.63351700 3.42480300

H 18.03986900 -1.41281500 2.20272400

H 17.85429300 0.32835100 2.54402200

C 20.69539200 -1.29192800 1.38560400

H 21.42276000 -1.14304900 0.58453900

H 20.21121100 -2.26511000 1.24310000

H 21.22226000 -1.31250200 2.34894500

C -3.34117100 -5.08401000 -4.75150600

H -3.76459300 -5.75208300 -5.51109900

H -2.26677000 -5.26153000 -4.69009000

H -3.50565400 -4.05196200 -5.08064900

C -3.69490500 -6.37070000 0.14039100

H -4.20510100 -6.91819200 0.94147200

H -2.62191400 -6.57785100 0.21422000

H -3.85221000 -5.30346600 0.31616200

C -4.12437100 -8.35415600 -1.33216200

H -4.36530300 -8.72011300 -2.33859400

H -4.82899400 -8.81798000 -0.63274500

H -3.12079600 -8.69959400 -1.06698400

H 20.38684800 1.30294900 2.80889000

H -5.72745300 -4.09190400 -3.86089900

H -7.20395800 -5.03437100 -2.06088400

H -6.26482700 -7.25319100 -1.93345500

O 18.29706200 2.47019600 1.69285900

H 17.83767100 3.28378300 1.40593100

O -5.65431100 -3.99245100 -1.23597200

H -4.67303200 -3.91881700 -1.36662200

H 18.66908400 2.83576200 -0.75997700

O -3.34013400 -4.64166500 -2.24795700

O 21.06092700 0.49411400 -1.08233300

H 21.50444300 0.96418600 -1.81714900

Chlbp

C 1.37526300 6.50823000 0.39136700

C 1.37862600 7.61177200 -0.52954300

C 2.18404300 7.21747600 -1.60555000

C 2.65075300 5.87560700 -1.31999600

N 2.16410200 5.46238100 -0.11408700

C 1.19101000 3.48694200 3.40122400

C 0.31637100 4.11832400 4.33276300

C -0.02414100 5.36131300 3.80778100

C 0.67909700 5.43385400 2.53427600

N 1.41300700 4.24507100 2.32146900

C 4.20144500 1.79940900 -0.98625100

C 4.91223000 1.75759800 -2.27187500

C 4.73174900 2.98776000 -2.86100100

C 3.91588400 3.79599400 -1.93814800

N 3.61743600 3.02567700 -0.80559800

C 3.49570400 0.72395100 1.16363200

C 3.40379100 -0.53416800 2.02394600

C 3.00392000 0.03694300 3.40927400

C 2.43940700 1.40050000 3.03907900

N 2.86284700 1.77859100 1.75689400

C 0.68379600 6.47724900 1.61706100

C 1.63974900 2.20287200 3.80890500

C 4.11828300 0.72646800 -0.08175600

H 4.59019000 -0.20229900 -0.38302300

C 3.48030100 5.08037400 -2.16789900

H 3.78667600 5.53269800 -3.10452300

H 0.10147400 7.35912600 1.86817900

Mg 2.54880600 3.64122700 0.78519100

C 5.69228200 0.58615200 -2.78304500

H 6.08525800 -0.02376700 -1.96391500

H 5.07317900 -0.04953500 -3.42617600

H 6.54017600 0.92480400 -3.38830100

C 2.49234500 7.98575200 -2.80044000

C -0.90727800 6.41325900 4.40567100

H -0.34083800 7.32555400 4.62932700

H -1.72028300 6.69101900 3.72445200

H -1.34915800 6.05482100 5.33811400

C 2.36172100 -1.51952600 1.45870400

H 2.63128000 -1.84110000 0.44647100

H 2.30362200 -2.40539900 2.09894900

H 1.36917400 -1.05452500 1.41461200

C 1.63777500 10.02093400 0.18958500

C 0.68386300 8.93076600 -0.34891300

H 1.09638200 10.96246700 0.33418200

H 2.07959200 9.72609300 1.14847100

H 2.44397300 10.20219500 -0.52694300

H -0.16843900 8.82140100 0.33169100

H 0.29625500 9.26692000 -1.31502900

C 5.24659800 3.47984000 -4.14201600

C 5.46789600 2.72769400 -5.23978500

H 5.46604900 4.54597700 -4.19310000

H 5.87251800 3.17432300 -6.14390300

H 5.22360300 1.67047100 -5.27438300

C 0.11956800 3.21865500 5.46304300

C 0.99367000 1.93350100 5.17245100

O -0.57333400 3.35085800 6.48351600

C 1.96683800 1.75820300 6.32062500

O 2.83882900 2.56540200 6.65758300

O 1.76510300 0.57339100 6.97708700

C 2.55916100 0.34053500 8.18555300

H 3.62624900 0.34766200 7.94704300

H 2.35068200 1.12136800 8.92097600

H 2.24295400 -0.63560100 8.55095100

C 4.75516700 -2.31827100 4.32150400

O 3.93940300 -3.20634600 4.03305000

O 6.03835300 -2.31733900 3.84203900

C 4.47666500 -1.11635400 5.18781100

C 4.21952400 0.16670500 4.36614100

H 5.11602900 0.42166000 3.78794600

H 4.06550200 0.98751400 5.07125200

H 5.33844400 -0.95387400 5.84240900

H 3.59651200 -1.34222600 5.79655600

C 6.43335900 -3.43549600 2.95130400

H 5.67077500 -3.51533300 2.16742100

H 6.41077900 -4.35734100 3.53568200

C 7.78151600 -3.10450700 2.39467100

C 8.90412000 -3.84716400 2.47913100

C 9.00807600 -5.17785800 3.19088900

H 9.38228000 -5.94717000 2.50307300

H 9.72397600 -5.11481000 4.01883000

H 8.05855200 -5.52939900 3.59954700

H 7.82334800 -2.15721000 1.85859000

H 0.33313200 1.06123700 5.16061000

C 10.18503800 -3.36455400 1.82292400

C 11.32063200 -3.06923600 2.82750800

C 12.54585900 -2.40392300 2.17458300

C 13.22614000 -3.20531200 1.04292900

C 13.69590200 -4.58761600 1.52735400

H 9.98238600 -2.45318300 1.24569200

H 10.52850600 -4.12502300 1.10706900

H 11.62645100 -3.99352300 3.33250900

H 10.93160000 -2.40180700 3.60832600

H 12.24310100 -1.42405600 1.77558500

H 13.29546700 -2.20178500 2.95459700

H 12.49323700 -3.35873700 0.23559900

H 14.42332700 -4.48611900 2.34389600

H 12.86011200 -5.19274600 1.89670700

H 14.17354800 -5.15180900 0.71953400

C 14.38580300 -2.37599600 0.44744100

C 15.08401900 -3.01084900 -0.77946300

C 16.44698300 -3.65175600 -0.45791200

C 17.17725400 -4.26315000 -1.67275900

C 16.42113200 -5.48398800 -2.22961300

C 18.62862500 -4.62249500 -1.28952200

C 19.52031700 -5.04490700 -2.47247300

C 20.99614300 -5.30248200 -2.08216500

C 21.39183400 -6.78005000 -1.84087000

C 21.36218900 -7.60048000 -3.14362000

H 13.98433300 -1.39089700 0.17142400

H 15.13058900 -2.19003500 1.23667000

H 14.41220800 -3.75330900 -1.23028800

H 15.24639200 -2.24291400 -1.54872500

H 17.09028500 -2.87684200 -0.01505900

H 16.32470100 -4.42606200 0.31330500

H 17.21860300 -3.49432200 -2.46261000

H 16.38057500 -6.28397200 -1.47740300

H 15.39234500 -5.23121700 -2.50734100

H 16.90779300 -5.88856400 -3.12321400

H 18.61061600 -5.41656900 -0.53005500

H 19.09038800 -3.74652900 -0.80974200

H 19.47823500 -4.24606500 -3.22664400

H 19.10573700 -5.93730300 -2.95675600

H 21.22777600 -4.72318700 -1.17609400

H 21.65432600 -4.91189700 -2.87091800

H 22.43407000 -6.76795500 -1.48605600

H 20.34609200 -7.66788200 -3.55127100

H 22.00244500 -7.14852900 -3.91118100

H 21.71529700 -8.62419200 -2.96877300

C 20.54143200 -7.45905600 -0.75299900

H 20.56539300 -6.89213500 0.18555800

H 20.91232800 -8.47070100 -0.54712100

H 19.49452800 -7.54833900 -1.06763300

H 2.24062700 -0.58453400 3.89094400

H 4.37686100 -1.03395100 2.06279700

H 3.10163000 7.47363700 -3.56521900

O 2.11900500 9.16058200 -3.01475500

C -17.85807300 -3.72443100 0.15457400

C -17.27634100 -2.64558400 0.71868400

H -17.29006200 -4.32100800 -0.55115400

C -15.91710600 -2.20193300 0.44528400

C -15.25238400 -1.13860900 0.98492900

C -13.91848800 -1.05348800 0.42503200

H -15.63627500 -0.44482200 1.72051600

C -12.93978000 -0.14993800 0.68456900

C -11.60235800 -0.04981100 0.12374700

H -13.20897800 0.60697400 1.41999800

C -10.81849300 0.98142400 0.57526300

C -9.46796100 1.29945700 0.17102400

H -11.24687400 1.65057200 1.32395300

C -8.77802400 2.35584000 0.68985700

H -8.99445800 0.67004200 -0.57718500

C -5.24977100 2.65840300 -0.81960500

H -9.28440700 2.96703200 1.43779100

H -4.95023800 3.55079300 -0.26785200

C -4.35026900 2.12311600 -1.69348600

C -3.04132400 2.78529900 -1.84492300

C -2.06720800 2.36384200 -2.63793100

H -2.88367200 3.68808900 -1.25300000

C -11.14745600 -1.05765900 -0.90647100

H -10.12609500 -0.86878000 -1.24104000

H -11.80594400 -1.04658100 -1.78063100

H -11.19750700 -2.07468100 -0.50533900

C -4.59977900 0.89889100 -2.54054800

H -5.51549500 0.37273700 -2.26500200

H -4.67240800 1.17629300 -3.60000400

H -3.76198400 0.19692800 -2.46035200

C -20.77523400 -5.93693100 0.86270800

C -19.49496500 -5.68761500 0.45774400

C -19.26627900 -4.16935500 0.45457200

C -20.34127600 -3.71197900 -0.70306900

C -21.75622100 -4.40168900 -0.55702700

C -21.81993700 -5.96144500 -0.22223900

H -22.30371900 -4.21625500 -1.49294100

C -1.09752100 1.94209700 -3.42198100

C -0.05627000 0.95160100 -2.88052500

C 1.36384600 1.34594200 -3.32160700

C 1.51820400 1.72592100 -4.79332800

C 0.46273700 2.72533700 -5.24915400

C -1.00744900 2.37798900 -4.89972200

H 2.52019200 2.14266300 -4.94178800

H 0.71526900 3.68435200 -4.77503400

C -0.09405900 0.82394200 -1.35318800

H -1.06173100 0.43339700 -1.02738100

H 0.06443700 1.79224700 -0.86794300

H 0.69500400 0.13995200 -1.01338300

C -1.55093300 1.24484300 -5.80479200

H -1.44846000 1.52961100 -6.85990400

H -2.61431000 1.08088300 -5.59348400

H -1.01972200 0.30957800 -5.63757900

C -1.85001200 3.64312200 -5.15100200

H -1.53358300 4.47291900 -4.50815800

H -1.74569200 3.95819400 -6.19639900

H -2.91185000 3.45679500 -4.96075400

C -19.79285100 -4.02157700 -2.11618700

H -20.44193300 -3.54247300 -2.85942700

H -18.78508100 -3.61877400 -2.25967000

H -19.78369500 -5.09208000 -2.32715800

C -18.69766500 -6.60413400 -0.43173700

H -18.21854100 -6.11417800 -1.28319300

H -19.33294700 -7.41264300 -0.80024500

H -17.89255700 -7.05275800 0.16814700

H 0.54985200 2.86865800 -6.33258600

H -22.83308700 -6.20527200 0.12810000

H -22.30534700 -3.89209300 0.24364100

O 1.41882300 0.54532800 -5.68767800

O -21.47452300 -6.79296300 -1.35593200

H -22.18074200 -6.76438000 -2.03002000

H -21.13863200 -5.24965300 1.62704500

H -19.25030800 -3.89193600 2.44216000

C -20.54354400 -2.18621300 -0.58660500

H -21.27701600 -1.84597100 -1.32774300

H -20.89309100 -1.91103300 0.41083000

H -19.60454700 -1.65673200 -0.78084400

O -19.74335000 -3.53032600 1.67814400

H 2.06665800 0.54418900 -3.06776200

H 1.67168900 2.22259900 -2.73854300

C 2.17941500 -1.44473800 -6.71985900

C 2.46511600 -0.31736400 -5.76723300

H 1.25049200 -1.94562300 -6.43200100

H 2.03585600 -1.04805900 -7.72970400

H 3.00803600 -2.15342500 -6.71729100

O 3.51948800 -0.16725800 -5.12669100

O -0.41729800 -0.35734900 -3.45897100

H 0.15773100 -1.05350000 -3.07947400

C -14.98972900 -2.85600100 -0.51662200

O -15.12799700 -3.83604500 -1.24318300

O -13.78060500 -2.11933500 -0.49427500

C -7.42321500 2.77127900 0.35383600

C -6.58601100 2.17157000 -0.53821100

H -7.05663200 3.65449400 0.87665300

H -6.92747700 1.28855700 -1.07328900

H -17.84920500 -2.04523700 1.42206400

chlbv

C -5.87909500 -7.74331700 0.67443400

C -6.51231400 -8.67510600 -0.21876600

C -6.90094300 -7.93380900 -1.34144200

C -6.48855400 -6.56385000 -1.11152300

N -5.88227700 -6.46078400 0.10785000

C -4.02964700 -5.27508900 3.62435000

C -3.71865500 -6.25732200 4.61041200

C -4.16059300 -7.48480500 4.12974300

C -4.74005300 -7.18777500 2.82457400

N -4.62718200 -5.80620700 2.55183400

C -5.33692100 -2.34678600 -0.94152400

C -5.83478500 -1.95911800 -2.26720200

C -6.39790600 -3.08611500 -2.82264500

C -6.25752000 -4.17088800 -1.83703300

N -5.60363400 -3.66916300 -0.70639100

C -4.19478200 -1.79164400 1.21747500

C -3.40082400 -0.79133700 2.05317100

C -3.45040600 -1.42459400 3.46829000

C -3.79420400 -2.87344600 3.15536600

N -4.32318600 -2.98834200 1.86292300

C -5.33418400 -8.06923600 1.93234200

C -3.64500600 -3.95669200 3.98160700

C -4.66428900 -1.48602700 -0.05675800

H -4.48169500 -0.47452600 -0.40284500

C -6.66041700 -5.47546000 -2.01681900

H -7.14755500 -5.70696000 -2.95774200

H -5.38946600 -9.11245900 2.22922500

Mg -5.13466600 -4.72496200 0.94444700

C -5.75182000 -0.57496800 -2.83577200

H -5.82041100 0.18230600 -2.04869300

H -4.80593300 -0.40882100 -3.36837900

H -6.56473800 -0.39532700 -3.54596700

C -7.57714300 -8.42857400 -2.53006900

C -4.07593500 -8.82800200 4.78676400

H -5.07251100 -9.25910600 4.94059700

H -3.50286800 -9.53618600 4.17633200

H -3.58828900 -8.74608100 5.76069300

C -1.96355500 -0.63574700 1.51752300

H -1.96212300 -0.27717500 0.48235100

H -1.41492700 0.08343600 2.13372400

H -1.43220000 -1.59480300 1.54172900

C -8.15937400 -10.43030700 0.55306600

C -6.73598700 -10.13956300 0.02647500

H -8.28240400 -11.50212700 0.74403700

H -8.35899700 -9.88881100 1.48509800

H -8.90359800 -10.13300900 -0.19128600

H -5.99606900 -10.52319800 0.73845400

H -6.60458300 -10.68379700 -0.91319400

C -7.04119900 -3.25488700 -4.12880400

C -6.74937200 -2.57211300 -5.25599900

H -7.82394700 -4.01032000 -4.18152600

H -7.29273200 -2.76325400 -6.17612500

H -5.96394400 -1.82421200 -5.30121100

C -3.05876400 -5.59370900 5.72918800

C -2.99988000 -4.05558100 5.36860000

O -2.60449600 -6.06074400 6.78441300

C -3.71321900 -3.28913500 6.46431800

O -4.90021500 -3.41888100 6.77994200

O -2.87257800 -2.41334900 7.09781000

C -3.40912100 -1.70059500 8.25945100

H -4.26856300 -1.09059400 7.96799600

H -3.72064700 -2.41841300 9.02211900

H -2.58850100 -1.08041300 8.61757900

C -3.53002900 1.56470100 4.23152000

O -2.35248500 1.81081000 3.93268600

O -4.58111500 2.28006700 3.72124600

C -3.99632000 0.46020000 5.14537900

C -4.52666900 -0.76937000 4.37416600

H -5.39241400 -0.47974600 3.76617400

H -4.89586400 -1.48876700 5.10933600

H -4.79513900 0.85308800 5.78189300

H -3.14924700 0.16206500 5.76975800

C -4.26573700 3.37739800 2.77655200

H -3.65815200 2.95021100 1.96927200

H -3.65516100 4.11498400 3.30085800

C -5.57246700 3.91354900 2.28270700

C -5.98251600 5.19808000 2.29043300

C -5.17130700 6.35947100 2.82081700

H -5.06056200 7.12870000 2.04559900

H -5.68283100 6.83226400 3.66763800

H -4.17022200 6.07657700 3.15286100

H -6.23885800 3.15499600 1.87410800

H -1.95245200 -3.73913000 5.36865200

C -7.34833000 5.56272100 1.73732000

C -8.31510700 6.11326300 2.80918700

C -9.75673400 6.27784800 2.29571500

C -9.94645200 7.23115300 1.09536500

C -9.44585600 8.65091500 1.41096100

H -7.80592700 4.67881500 1.27458200

H -7.22730700 6.31242700 0.94227900

H -7.94537200 7.07166600 3.19367000

H -8.32297100 5.41900000 3.66038900

H -10.14519900 5.28607000 2.01876100

H -10.38729500 6.63609300 3.12354600

H -9.36172800 6.84500400 0.24592600

H -9.99286900 9.07404300 2.26409400

H -8.37865100 8.65751600 1.66026500

H -9.58511200 9.32122300 0.55633300

C -11.43090700 7.22233600 0.66661600

C -11.75778700 8.05371500 -0.59731400

C -12.43543800 9.40321600 -0.29526100

C -12.84969200 10.21418300 -1.54126800

C -11.62518800 10.64253900 -2.37155300

C -13.69879700 11.43275800 -1.11993000

C -14.41274600 12.14891800 -2.28198300

C -15.30010900 13.33471800 -1.83152500

C -14.67655400 14.74476300 -1.97325900

C -14.49866200 15.14496000 -3.44943800

H -11.72470200 6.17602200 0.50314400

H -12.04274200 7.58137500 1.50851100

H -10.83569900 8.21275400 -1.17192600

H -12.42814700 7.47946400 -1.25223800

H -13.33489200 9.20507300 0.30649000

H -11.77679100 10.02134800 0.33201600

H -13.48198600 9.56476800 -2.16984800

H -10.95352800 11.26722500 -1.76656100

H -11.05235300 9.77941700 -2.72688700

H -11.91813800 11.22202400 -3.25348300

H -13.05924400 12.14293400 -0.57709700

H -14.46457900 11.09764700 -0.40438500

H -15.03373600 11.40651600 -2.80311600

H -13.67778000 12.49510100 -3.01884800

H -15.58811700 13.18046200 -0.78116600

H -16.23547700 13.33007500 -2.40844200

H -15.39610600 15.44933000 -1.52818300

H -13.77902100 14.49151100 -3.95773100

H -15.44951000 15.08347400 -3.99304400

H -14.12658100 16.17332200 -3.53377100

C -13.35044200 14.89242100 -1.20665300

H -13.47025600 14.63736900 -0.14665200

H -12.98148000 15.92378400 -1.26672900

H -12.57568800 14.23901600 -1.62623500

H -2.47828300 -1.35459200 3.96930900

H -3.89262200 0.18644100 2.03394900

H -7.73693900 -7.69651300 -3.34082600

O -7.97830600 -9.60229000 -2.69052600

C 19.68866500 2.74639400 1.30373500

C 18.52262800 2.58490100 0.64509000

H 19.89408600 2.18416500 2.21006500

C 17.43240900 1.68264000 1.03762300

H 18.36756500 3.17877300 -0.25456000

C 16.31533600 1.64542300 0.25628700

C 15.12430700 0.83711000 0.45002600

H 16.29917500 2.29264500 -0.62230500

C 14.06151000 0.88086700 -0.39803100

H 15.08787100 0.17328500 1.30922700

C 12.83077600 0.10322600 -0.27481600

H 14.11541700 1.55338300 -1.25575900

C 11.85764200 0.27182800 -1.22027600

C 10.57135900 -0.39002700 -1.29188500

H 12.05811000 0.98512600 -2.02176100

C 9.66098200 -0.15157500 -2.27821000

H 10.31538400 -1.11489800 -0.52107200

C 8.37532200 -0.81464700 -2.34956000

H 9.91652700 0.57341600 -3.04923400

H 8.17844000 -1.53221500 -1.55113600

C 7.39884900 -0.64343500 -3.29111800

C 6.17054600 -1.42534600 -3.16838600

C 5.10217900 -1.37696700 -4.00906000

H 6.12409300 -2.10681500 -2.31743200

C 3.91611800 -2.19317600 -3.81723800

H 5.13045500 -0.70416000 -4.86181200

H 3.94476900 -2.85699300 -2.95155900

C 2.79129400 -2.22283000 -4.58780800

C 1.70959900 -3.13860400 -4.20201200

C 0.53101100 -3.28336600 -4.84148100

H 1.87851300 -3.75227600 -3.31803200

H 0.30645700 -2.69872600 -5.72917000

C 17.61651400 0.85017700 2.28506400

H 16.75876300 0.20794200 2.49063300

H 17.77078300 1.49176200 3.16204700

H 18.50095300 0.20673400 2.19489600

C 12.70601400 -0.84575600 0.89534900

H 11.75520600 -1.38044100 0.90044200

H 12.78871800 -0.30312400 1.84531300

H 13.50900400 -1.59307100 0.87559300

C 7.51801500 0.31208900 -4.45655700

H 8.46792700 0.84848600 -4.46245200

H 7.43222800 -0.22599100 -5.40872200

H 6.71364200 1.05767900 -4.42984600

C 2.59135500 -1.36996300 -5.81901200

H 1.70561100 -0.73146600 -5.70942300

H 2.42812400 -1.99957700 -6.70274000

H 3.44516000 -0.72223500 -6.02449600

C 21.92705000 6.00912600 1.17086800

C 20.67798300 5.14639300 1.18740200

C 20.77059000 3.67313900 0.85297000

C 22.13263800 3.00678400 0.52655800

C 23.32174300 4.00785900 0.57606600

C 23.00890100 5.46803700 0.22689500

H 22.32533200 6.07876000 2.19134200

H 24.10866200 3.64482300 -0.09472200

C -0.52734500 -4.24577200 -4.41300300

C -1.44841800 -3.88751500 -3.26956300

C -2.80567500 -4.55131300 -3.10611600

C -3.28121800 -5.36777200 -4.31213000

C -2.13561500 -6.13848400 -4.95889100

C -0.95335500 -5.28153200 -5.47319900

H -2.73569700 -5.21110000 -2.23095100

H -4.03919600 -6.08676100 -3.96838900

H -1.75634500 -6.84263300 -4.20782300

C -1.31375000 -2.57437000 -2.53413200

H -0.28884600 -2.20230400 -2.55902000

H -1.61490800 -2.70082700 -1.48673100

H -1.97155300 -1.82029200 -2.98427500

C -1.32377900 -4.57682900 -6.79800100

H -1.72098200 -5.31499500 -7.50402600

H -0.44420400 -4.11778900 -7.26287700

H -2.10088500 -3.82490000 -6.64698000

C 0.23955000 -6.22959800 -5.72009400

H 0.56410300 -6.69492300 -4.78302000

H 1.09308000 -5.69254300 -6.14745700

H -0.04751700 -7.02296900 -6.42121900

C 19.53038100 5.70935600 1.98804100

H 19.83017800 5.85264200 3.03326600

H 18.65964300 5.05292200 1.96145500

H 19.24158400 6.68598800 1.58331400

C 22.02296500 2.36906900 -0.87739000

H 22.91350600 1.76178000 -1.07912500

H 21.14374100 1.71878000 -0.94561500

H 21.95237500 3.13008400 -1.65803300

C 22.43702100 1.88736200 1.54854100

H 22.38163400 2.25103100 2.58279400

H 23.45501300 1.51658400 1.38237000

H 21.75354100 1.03944000 1.44018600

H -2.53060600 -6.72600100 -5.79562200

H 21.65064400 7.02270000 0.85574400

H 23.92253700 6.05782000 0.34788700

H 23.74226300 4.00910000 1.59204000

O -3.88554500 -4.50247100 -5.32512900

H -4.75637800 -4.16932900 -5.02745600

O 22.63999300 5.66135200 -1.16471700

H 21.71204700 5.35308800 -1.27929400

H -3.55691200 -3.78902100 -2.87370800

O 20.30354300 4.65142500 -0.18590700

O -0.30319100 -4.83451100 -3.05809400

chlbx

C -0.77519200 -1.04833900 3.12763000

C -1.48567300 0.10615700 3.60355800

C -0.84814500 1.21652100 3.03468800

C 0.23827700 0.71264300 2.21773800

N 0.27692900 -0.64847400 2.28788500

C 1.13854700 -4.69430000 2.00394200

C 0.29826000 -5.63174400 2.67292500

C -0.69096100 -4.90797800 3.33202500

C -0.40168700 -3.51407000 3.02365400

N 0.73639000 -3.43214600 2.18938800

C 3.54766700 -0.29827200 -0.45552200

C 3.89686400 1.07187500 -0.85481100

C 3.03983900 1.91263600 -0.18236100

C 2.16088800 1.06007000 0.64008800

N 2.51519500 -0.28071800 0.44266400

C 3.91366100 -2.77444700 -0.56171600

C 4.60811600 -3.96416600 -1.22077500

C 4.30077600 -5.12030900 -0.23324500

C 3.10223800 -4.57550400 0.52943600

N 3.00692700 -3.18621600 0.37445500

C -1.08545500 -2.38304600 3.45104300

C 2.21288400 -5.28802600 1.29009600

C 4.17616900 -1.46116700 -0.93829400

H 4.94573400 -1.32452100 -1.69018500

C 1.13632100 1.50615700 1.44100000

H 0.97855200 2.57807600 1.47103600

H -1.94189400 -2.53845600 4.10081700

Mg 1.64805200 -1.86551100 1.33300900

C 5.01391000 1.43007200 -1.78761800

H 5.82302200 0.69442100 -1.74405800

H 4.67365200 1.48377400 -2.83026800

H 5.43397000 2.40746400 -1.52956400

C -1.19982800 2.61519500 3.19671900

C -1.81279300 -5.42490800 4.17959700

H -1.75412800 -5.03136200 5.20155700

H -2.78869600 -5.13475600 3.77200800

H -1.77753600 -6.51531600 4.23398900

C 4.04846200 -4.22051500 -2.63406800

H 4.20151100 -3.35197900 -3.28381800

H 4.55264800 -5.08299000 -3.08128900

H 2.97177300 -4.42506700 -2.59511000

C -2.22716800 0.38529300 6.00372600

C -2.65616000 0.11451500 4.54407900

H -3.09850300 0.36123200 6.66783100

H -1.50676000 -0.36349300 6.35291000

H -1.76906900 1.37540600 6.08288800

H -3.19370300 -0.83933600 4.49101000

H -3.35090300 0.90385500 4.24219800

C 2.95882500 3.37465600 -0.18552700

C 3.34041400 4.21346500 -1.17052700

H 2.53218500 3.82422900 0.70820500

H 3.21862900 5.28662300 -1.04688600

H 3.73965300 3.85727400 -2.11554600

C 0.79949500 -6.97363800 2.40445200

C 2.08666900 -6.80082200 1.50166400

O 0.36961500 -8.07985900 2.76675700

C 3.25211400 -7.44906600 2.22097100

O 3.69809800 -7.11429600 3.32306700

O 3.77280000 -8.49257900 1.50197000

C 4.82244000 -9.28370300 2.14744700

H 5.68748500 -8.65483300 2.37537800

H 4.44094300 -9.71845600 3.07454900

H 5.07813800 -10.05999600 1.42742700

C 7.08566200 -6.02175400 -1.16874300

O 6.71013800 -6.36717700 -2.29875400

O 8.11954500 -5.14325300 -0.97818800

C 6.47416100 -6.46320600 0.13612500

C 5.49173600 -5.42050900 0.71602500

H 6.02767700 -4.48982200 0.93861300

H 5.13006500 -5.80229600 1.67413200

H 7.27961800 -6.63483900 0.85659500

H 5.94242100 -7.40176000 -0.04491700

C 8.78074400 -4.60090500 -2.18767300

H 8.00705800 -4.10752300 -2.78914700

H 9.17290900 -5.43793800 -2.76846500

C 9.83116500 -3.64369400 -1.71864500

C 11.12878700 -3.61657000 -2.08376600

C 11.77229100 -4.58468000 -3.05229900

H 12.26778900 -4.03510800 -3.86313000

H 12.54627500 -5.17621800 -2.54903700

H 11.06482000 -5.28085800 -3.50759500

H 9.46992200 -2.89538200 -1.01453400

H 1.92860500 -7.34622900 0.56635400

C 12.05617800 -2.55502500 -1.52021100

C 13.20930900 -3.13409500 -0.67154900

C 14.02451400 -2.05353300 0.06133900

C 14.70207400 -0.99067000 -0.83151300

C 15.64469600 -1.63058800 -1.86476300

H 11.48070800 -1.85516500 -0.90066300

H 12.47942200 -1.97173200 -2.35061500

H 13.87493800 -3.73615800 -1.30207500

H 12.78394500 -3.81895200 0.07461000

H 13.36413100 -1.53929100 0.77578000

H 14.80371600 -2.54770500 0.66149300

H 13.91901000 -0.44324300 -1.37908700

H 16.44231000 -2.19662600 -1.36516400

H 15.10931400 -2.31970100 -2.52778300

H 16.11658100 -0.87115700 -2.49686800

C 15.43274500 0.03584100 0.06256200

C 16.06389400 1.23411100 -0.68655800

C 17.58983800 1.12572800 -0.86489800

C 18.25502200 2.35986500 -1.50992700

C 17.75186200 2.58677600 -2.94785100

C 19.79160000 2.21724300 -1.46581200

C 20.56302900 3.51117000 -1.78747900

C 22.10126500 3.35759000 -1.70348700

C 22.83859500 3.14515200 -3.04843900

C 22.78572600 4.40344800 -3.93426600

H 14.70991900 0.40766800 0.80237300

H 16.21221000 -0.48845700 0.63675900

H 15.57468900 1.34501200 -1.66346200

H 15.85517300 2.16223100 -0.13578600

H 18.03784800 0.96676600 0.12734400

H 17.83634500 0.23390700 -1.45924600

H 17.98188800 3.24190900 -0.90648700

H 17.96641900 1.70725600 -3.57051900

H 16.67149500 2.76357200 -2.97685200

H 18.23485700 3.45274800 -3.41284300

H 20.09664600 1.41704200 -2.15460000

H 20.08594400 1.88640800 -0.45854800

H 20.23451300 4.28305600 -1.07717800

H 20.28192900 3.87990800 -2.78146500

H 22.33594600 2.51562100 -1.03561700

H 22.53195500 4.25133200 -1.23073200

H 23.89580000 2.96561100 -2.79869600

H 21.75736000 4.63930100 -4.23430800

H 23.18955600 5.27628800 -3.40664700

H 23.37179200 4.26108600 -4.85047200

C 22.33148200 1.91487200 -3.82146800

H 22.38890600 1.00642800 -3.20955100

H 22.93080500 1.75226500 -4.72573500

H 21.28859700 2.04587800 -4.13504600

H 4.03645500 -6.03940700 -0.76826900

H 5.68439100 -3.77707900 -1.29619700

H -0.62900500 3.33384200 2.58652500

O -2.09167500 3.05163600 3.96206700

C -1.65825300 7.26177600 2.11177400

C -2.72831400 6.78775500 1.42901100

H -1.63600000 7.11087100 3.18822400

C -3.89913900 6.12011100 2.01106100

H -2.73524600 6.87504900 0.34323400

C -4.88582600 5.71011600 1.16036400

C -6.11393400 5.01796500 1.50479500

H -4.74864000 5.91186500 0.09623500

C -7.04848900 4.66575800 0.58051900

H -6.28021800 4.76746700 2.54869500

C -8.30022100 3.96172500 0.84862500

H -6.86263100 4.92337200 -0.46355800

C -9.13622600 3.69432100 -0.20022900

C -10.41210700 3.01042300 -0.16060800

H -8.81874200 4.02908400 -1.18938500

C -11.18740700 2.80151300 -1.26253100

H -10.77422800 2.64183600 0.79780800

C -12.46040000 2.11200700 -1.21799300

H -10.82857100 3.17178900 -2.22138600

H -12.75834400 1.75270500 -0.23122600

C -13.31498600 1.86520200 -2.25639600

C -14.55301800 1.14036400 -1.98033700

C -15.51586200 0.82038100 -2.88667400

H -14.70215000 0.83425200 -0.94347500

C -16.71850600 0.08902600 -2.52802500

H -15.38701100 1.11964700 -3.92323500

H -16.78920100 -0.19692000 -1.47686600

C -17.75071300 -0.27203700 -3.34318800

C -18.87239800 -1.01877200 -2.75754300

C -19.97617600 -1.44006700 -3.41029300

H -18.78147200 -1.23550500 -1.69247500

H -20.08090600 -1.20951600 -4.46905600

C -3.93771900 5.90825100 3.50630300

H -4.91487000 5.56318200 3.84992800

H -3.71276700 6.84122100 4.03686000

H -3.19763600 5.15682400 3.80972100

C -8.59954600 3.56848900 2.27745200

H -9.54848300 3.03935500 2.37625500

H -8.64328400 4.45535400 2.92177600

H -7.81011000 2.91791100 2.67330600

C -13.05089000 2.30190300 -3.67934700

H -12.10379400 2.83321300 -3.78414300

H -13.02510200 1.43601100 -4.35266200

H -13.84763700 2.96742500 -4.03447400

C -17.80963200 0.05188900 -4.81783500

H -18.69344200 0.66107800 -5.04579500

H -17.88685500 -0.86610600 -5.41381900

H -16.93075600 0.60004700 -5.16079600

C 0.68598400 9.38840500 -0.17410400

C -0.54416100 8.80896200 0.50062700

C -0.46636100 7.90905700 1.51569800

C 0.87686700 7.47654000 2.14234800

C 2.07671600 8.26586500 1.56228400

C 1.97152600 8.60545800 0.08277100

H 0.83252300 10.42628500 0.16168500

H 2.99854200 7.70105100 1.74495700

C -21.14545500 -2.18323100 -2.78654000

C -20.75130300 -3.31283700 -1.82776600

C -21.53974000 -3.67376700 -0.79688800

C -22.85881100 -3.02882100 -0.45469500

C -23.32856700 -2.05892300 -1.53586800

C -22.20364400 -1.20452100 -2.16104600

H -21.24574800 -4.52326400 -0.17874700

H -23.61370900 -3.82158700 -0.33683500

H -23.81256300 -2.64364200 -2.33129900

C -19.52118100 -4.12685000 -2.16496800

H -18.59633800 -3.55967400 -2.02669200

H -19.54061200 -4.43078000 -3.21981400

H -19.46895400 -5.02959400 -1.54805900

C -22.82039600 -0.34142900 -3.27938900

H -23.60066700 0.31180600 -2.86998900

H -23.27804000 -0.96215400 -4.06145400

H -22.06512900 0.29842700 -3.74897000

C -21.58335800 -0.26973200 -1.10381200

H -22.37473700 0.30624500 -0.61130500

H -20.88370900 0.43226900 -1.56756400

H -21.06434100 -0.82601100 -0.32038900

C -1.83026700 9.37887800 -0.05867000

H -1.70206700 10.45089700 -0.26163400

H -2.67289700 9.25699600 0.62431900

H -2.10297500 8.90849700 -1.01418600

C 1.07946500 5.95984600 1.91465600

H 1.99558500 5.62167500 2.41781500

H 0.23436500 5.39692700 2.32693100

H 1.16528000 5.74908500 0.84650800

C 0.86806000 7.74620500 3.66959500

H 0.56966200 8.77826700 3.88963700

H 1.87477300 7.58868400 4.07672600

H 0.19535800 7.07082600 4.20737700

H -21.66570100 -2.68585900 -3.62057000

H -24.09295600 -1.40696500 -1.09726700

H 0.49839400 9.45829400 -1.25790200

H 2.83727800 9.21428700 -0.21701700

H 2.17288800 9.22021200 2.09818800

O -22.80720300 -2.28900600 0.81667200

H -22.39269400 -2.83563800 1.51386000

O 2.01475100 7.35513200 -0.67977400

H 1.70309000 7.49882500 -1.59484200

Chlbz

C 6.54212500 -7.00281200 -1.06596000

C 7.15935100 -7.61446900 -2.21050800

C 8.08729700 -6.68358900 -2.69563500

C 8.02135800 -5.52412100 -1.82832200

N 7.08098600 -5.72336300 -0.86077000

C 4.38346200 -5.46491300 2.27696500

C 3.54502100 -6.58123200 2.56781700

C 3.85758100 -7.58523000 1.65619300

C 4.90782300 -7.01680700 0.82117800

N 5.20915700 -5.70614200 1.25367100

C 8.27437000 -1.85985900 0.54472600

C 9.35783500 -1.21570900 -0.21125700

C 9.68513900 -2.06943500 -1.23940800

C 8.80043400 -3.24295000 -1.12326600

N 7.95701000 -3.06483000 -0.02008900

C 6.60425200 -1.85876000 2.41188600

C 6.06621700 -1.20464300 3.68245000

C 4.70608200 -1.92505700 3.87778000

C 4.89357900 -3.18499000 3.04559300

N 5.94896400 -3.02517100 2.13901400

C 5.54425600 -7.59661800 -0.26866800

C 4.16090400 -4.34079800 3.11554500

C 7.66044000 -1.31476800 1.68684800

H 8.05044700 -0.36912200 2.04723000

C 8.82344300 -4.34798500 -1.94193000

H 9.54829300 -4.33702600 -2.74806900

H 5.23959400 -8.60279100 -0.54190200

Mg 6.53214900 -4.35373100 0.58718700

C 9.92317400 0.13706200 0.09952400

H 9.16480400 0.79509000 0.53509700

H 10.75491600 0.07707200 0.81382600

H 10.30322900 0.61705900 -0.80766400

C 8.96921100 -6.83807500 -3.84055000

C 3.25397900 -8.95046600 1.53228300

H 2.76733500 -9.08277400 0.55837300

H 4.01428100 -9.73478000 1.62843700

H 2.50235700 -9.10712700 2.30925900

C 7.03013300 -1.41305800 4.86716500

H 8.01130300 -0.97176000 4.66099900

H 6.61947300 -0.94244500 5.76603000

H 7.17779900 -2.48124100 5.06660500

C 5.84789900 -8.88059100 -3.96137800

C 6.84330100 -8.96641500 -2.78269200

H 5.61885000 -9.88268000 -4.34057600

H 4.90802700 -8.40592900 -3.65428600

H 6.28542800 -8.30195300 -4.78031900

H 6.43453800 -9.62234900 -2.00539900

H 7.76582600 -9.42387500 -3.15153900

C 10.67669500 -1.90328000 -2.30527400

C 11.82589300 -1.20313400 -2.22560500

H 10.44936600 -2.40003700 -3.24765000

H 12.49189100 -1.12748600 -3.07996600

H 12.14849200 -0.71533200 -1.31151300

C 2.68705600 -6.23236500 3.69295900

C 3.04240800 -4.74077000 4.08361700

O 1.82510300 -6.89987500 4.28529500

C 1.77662200 -3.91565500 3.97299600

O 1.12588500 -3.72646500 2.93962300

O 1.40891400 -3.38933300 5.18078200

C 0.13350000 -2.67159200 5.23678300

H 0.14878000 -1.81474300 4.55760400

H -0.68086800 -3.34238200 4.95232600

H 0.03216800 -2.35133800 6.27280700

C 3.95363900 0.85747800 4.95231400

O 4.60004800 0.75851500 6.00568300

O 4.11971600 1.90467400 4.08554700

C 2.93674200 -0.13396900 4.44693800

C 3.51023600 -1.07777400 3.36616200

H 3.82130200 -0.49379600 2.49139400

H 2.69855700 -1.72718100 3.02830000

H 2.09259500 0.42049500 4.02568200

H 2.59322600 -0.72430200 5.30134500

C 5.11863600 2.93808700 4.45007800

H 6.07487500 2.42543000 4.61071100

H 4.81533100 3.38540800 5.39844900

C 5.18172500 3.90940000 3.31425600

C 4.99750200 5.24415000 3.37259900

C 4.67216600 6.01690600 4.63176800

H 5.41234600 6.81197900 4.78951300

H 3.69458900 6.50496300 4.54124800

H 4.65175800 5.39787900 5.53098000

H 5.41016900 3.45649100 2.35040400

H 3.36051800 -4.72320700 5.13030700

C 5.11784800 6.08446000 2.11404000

C 3.78862300 6.74561300 1.68700900

C 3.87748800 7.45656400 0.32417800

C 4.91180700 8.59934400 0.22234100

C 4.65275000 9.69650400 1.26864400

H 5.47955600 5.46074600 1.28651400

H 5.87226500 6.86729100 2.27702900

H 3.45775800 7.45571800 2.45455000

H 3.01552000 5.96765200 1.62881700

H 4.10674900 6.70727100 -0.44853400

H 2.88623400 7.86421400 0.07439800

H 5.91344200 8.18130600 0.40801200

H 3.65502000 10.13458800 1.13182200

H 4.70888900 9.30310300 2.28997000

H 5.38843100 10.50363200 1.19006300

C 4.91696400 9.16141700 -1.21702500

C 5.99453400 10.23639400 -1.49848600

C 5.44399600 11.67434800 -1.53201400

C 6.49498900 12.76428300 -1.83268700

C 7.51692400 12.89278300 -0.68764300

C 5.79094400 14.10818700 -2.11744100

C 6.71947300 15.22920800 -2.62109100

C 5.97590800 16.53614600 -2.98987800

C 5.98024900 17.64972800 -1.91382600

C 7.38624700 18.24463600 -1.71301300

H 5.05412300 8.31570200 -1.90521100

H 3.92122000 9.57533200 -1.43889800

H 6.79083000 10.15150300 -0.74699100

H 6.47077300 10.03576500 -2.46841300

H 4.66052800 11.71975400 -2.30305000

H 4.94925200 11.90859500 -0.57835300

H 7.03704300 12.46748300 -2.74632200

H 7.01748100 13.21878500 0.23525100

H 8.01562500 11.93981000 -0.48137900

H 8.29841500 13.62312900 -0.92215500

H 5.26708400 14.43476700 -1.20811300

H 5.01350700 13.94063600 -2.87794200

H 7.25360600 14.85222800 -3.50497800

H 7.49139000 15.44310900 -1.87214800

H 4.93305500 16.29080600 -3.23936900

H 6.41623900 16.96089200 -3.90293500

H 5.33608300 18.45574200 -2.29802700

H 8.08552500 17.49571900 -1.32126300

H 7.79482800 18.62281800 -2.65822300

H 7.36029000 19.07663500 -0.99867900

C 5.38989200 17.18749400 -0.57006500

H 4.37939700 16.78015600 -0.69583300

H 5.33193700 18.02573200 0.13519400

H 6.01309000 16.41090200 -0.11005800

H 4.53592700 -2.17536400 4.93095300

H 5.93164600 -0.12990300 3.52253300

H 9.69205800 -6.02210800 -4.01313000

O 8.96296200 -7.81384500 -4.62439600

C -19.13081200 1.13665800 -0.86589300

C -17.83983300 0.83642700 -0.58833200

H -19.45191100 1.10560700 -1.90466700

C -16.79822600 0.51851300 -1.57235200

H -17.52496300 0.80470800 0.45397000

C -15.54418500 0.23093300 -1.11651900

C -14.36751400 -0.09943500 -1.90020200

H -15.39031500 0.24857500 -0.03597800

C -13.15906100 -0.36800200 -1.33511600

H -14.46329600 -0.12865000 -2.98216200

C -11.92809900 -0.70040800 -2.04785600

H -13.08277300 -0.33460800 -0.24693200

C -10.79652700 -0.93704000 -1.31743800

C -9.47717800 -1.27295100 -1.81145700

H -10.88043500 -0.86606700 -0.23138900

C -8.40542200 -1.48544400 -0.99583100

H -9.32964800 -1.35803400 -2.88675300

C -7.08287900 -1.81572200 -1.48472200

H -8.55534800 -1.39968400 0.07922400

H -6.99386100 -1.89121100 -2.56990100

C -5.95209500 -2.03604200 -0.74775100

C -4.71104600 -2.35317500 -1.45047400

C -3.50074800 -2.58539900 -0.87348200

H -4.78007400 -2.40138100 -2.53846900

C -2.30682700 -2.89530300 -1.63891200

H -3.41364700 -2.53981900 0.20858200

H -2.44855700 -2.94806600 -2.71993800

C -1.04869300 -3.12086000 -1.15894800

C 0.01593700 -3.42404000 -2.12456200

C 1.31697600 -3.64291500 -1.82267100

H -0.28942400 -3.45530600 -3.17045500

H 1.62228800 -3.55745600 -0.78191500

C -17.17543900 0.53171900 -3.03567800

H -16.33424400 0.28828800 -3.68662100

H -17.55386700 1.51828400 -3.33078600

H -17.97339600 -0.19443300 -3.23600200

C -11.97661000 -0.76050200 -3.55763700

H -11.01263800 -1.02181500 -3.99661500

H -12.28238600 0.20722400 -3.97459500

H -12.70895800 -1.50564900 -3.89242400

C -5.91309300 -1.96751400 0.76219300

H -6.88450300 -1.72515500 1.19558600

H -5.58834500 -2.92660600 1.18428500

H -5.19813200 -1.20548600 1.09662200

C -0.68501800 -3.07759500 0.30611000

H 0.07285700 -2.30884800 0.50049000

H -0.25117300 -4.02487000 0.64255800

H -1.54258300 -2.86318500 0.94600000

C -21.01941800 2.57255300 2.23240400

C -19.96935400 2.29911800 1.17166000

C -20.18056400 1.45186700 0.13111300

C -21.53066300 0.74288700 -0.10762900

C -22.62965600 1.25321500 0.85568400

C -22.15748600 1.55429600 2.27100000

H -21.44462500 3.57492400 2.06867900

H -23.44207200 0.51811000 0.89192300

C 2.39066300 -3.95474900 -2.79697700

C 2.26003500 -4.92150100 -3.74133300

C 3.34396200 -5.23512900 -4.75583800

C 4.43006800 -4.16834800 -4.88507800

C 4.83019400 -3.65732500 -3.50842300

C 3.66706700 -3.09980600 -2.65396600

H 3.81829400 -6.19081900 -4.48329300

H 5.31083700 -4.60828400 -5.37703800

H 5.29512500 -4.50066800 -2.97820100

C 1.06588700 -5.84258000 -3.87364200

H 0.44618000 -5.85897500 -2.97554300

H 1.40612600 -6.86678600 -4.07969600

H 0.42553100 -5.54866100 -4.71730500

C 3.34919800 -1.63817400 -3.05305500

H 4.22246600 -0.99755700 -2.87076800

H 2.51111000 -1.25051200 -2.46251000

H 3.09990300 -1.57976600 -4.11375200

C 4.15720200 -3.10383900 -1.18380700

H 4.21297100 -4.13074500 -0.79784900

H 3.50084700 -2.52826900 -0.52360000

H 5.15089200 -2.63519500 -1.13957000

C -18.70805900 3.10760000 1.39032800

H -18.97060400 4.12103400 1.72350600

H -18.09913600 3.18927600 0.48821500

H -18.08060600 2.67002200 2.17983800

C -21.34354200 -0.78536400 0.05495000

H -22.27408000 -1.30887500 -0.20121100

H -20.55274600 -1.15326100 -0.60881000

H -21.08660700 -1.02970900 1.08733900

C -22.03970100 1.02307700 -1.54538400

H -22.03229100 2.09650000 -1.77169600

H -23.07073700 0.66172400 -1.64672700

H -21.44328000 0.50502900 -2.30342700

H 5.59774600 -2.88164900 -3.61870900

H -20.52720100 2.62059400 3.21664300

H -22.99524500 1.96768900 2.85344200

H -23.05267100 2.18606400 0.45701100

O 3.98246000 -3.02558700 -5.68020900

H 3.69708400 -3.31713100 -6.56830000

O -21.74486300 0.29499100 2.88623900

H -21.39373000 0.44875500 3.78519600

H 2.87565300 -5.41082900 -5.73715900
